# The Human Plastiphere: A Bioparticulate System Challenging Microplastic Risk Assessment and Governance

**DOI:** 10.1021/acs.est.5c05922

**Published:** 2025-10-01

**Authors:** V. C. Shruti, Gurusamy Kutralam-Muniasamy

**Affiliations:** † Lab 49, Contaminantes Emergentes, Department of Biotechnology and Bioengineering, Centro de Investigación y de Estudios Avanzados del Instituto Politécnico Nacional, Av Instituto Politécnico Nacional 2508, San Pedro Zacatenco, Gustavo A. Madero, 07360 Ciudad de México, México; ‡ 42576 CIITEC - IPN, Centro de Investigación e Innovación Tecnológica, Cda. de Cecati s/n, Santa Catarina, Azcapotzalco, 02250 Ciudad de México, México

**Keywords:** microplastics, human health, tissue distribution, exposure assessment, risk characterization

## Abstract

The infiltration of microplastics (MPs) into human tissues represents a paradigm shift in environmental health, transforming external pollution into internal biological integration. Drawing on 90 clinical studies (2016–2025), we define the human plastiphere as a bioparticulate system composed of nonendogenous plastic particles that accumulate, distribute, and interact with host tissues. This system displays key biological features: persistence (decade-scale tissue retention), organized distribution (organotropism across 63 human biological compartments), and active biological engagement (e.g., cardiovascular, reproductive, and metabolic interference). We identify eight unresolved paradoxes—ranging from size-defying barrier penetration to absent toxicity thresholds—that highlight critical gaps in synthetic particle biology. The plastiphere challenges conventional toxicology by showing that MPs: (1) follow selective biological rules (e.g., vascular trafficking) while violating others (e.g., phagocytic clearance), and (2) form a measurable, transgenerational burden with escalating health risks as plastic production continues to rise. To address this emerging bioparticulate phenomenon, we propose three urgent actions: harmonized detection protocols, polymer-specific safety thresholds, and source-targeted policy interventions. The plastiphere, both as a biological system and a conceptual framework, offers a roadmap for advancing science from descriptive detection to health-relevant, mechanistically grounded, and policy-actionable solutions.

## Graphical abstract

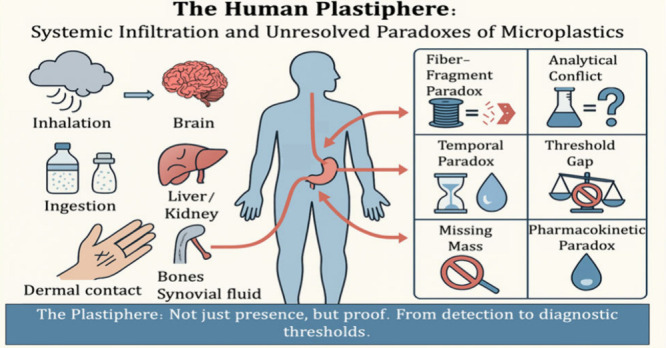

## 1 Introduction

Micro- and nanoplastics (MNPs) are increasingly detectable across a range of human tissues—including blood, lungs, placenta, reproductive fluids, and brain—raising pressing questions about their persistence, biodistribution, and potential biological effects.^1–3^ This internalization of synthetic particles marks a shift in exposure biology: plastics are no longer merely environmental contaminants but have become embedded within human physiology. In this review, we introduce the concept of the human plastiphere, defined as a biologically integrated network of synthetic polymer particles within the body. This system exhibits three defining properties: persistence, MNPs resisting biological clearance and accumulating in human tissues (Section 2 and 5.2); organization, as particles show nonrandom anatomical distribution (Table 1) and signs of organotropism (Text S1); and interaction, through their engagement with immune, endocrine, and metabolic pathways (Sections 4.1–4.5; Figure 1). Although not an organ system in the classical sense, the plastiphere functions as a bioparticulate system—a semipermanent, nonendogenous structure analogous to natural particulate networks such as extracellular vesicles, albeit of industrial origin.^4,5^

**Table 1:** Summary of MP Studies by Organ System, Sample Compartment, and Concentration Units^a^

Organ system	Sampled compartment	Cancerous?	# Studies	Country	Sample range	Polymer Detection method	Concentration (Particles/g)	Concentration (Particles/mL)	Concentration (Particles/sample)	Concentration (Microgram/g)	All References
**Gastrointestinal**	Colon (tumoral)	Y	1	Turkey (*n* = 1)	11–30 (*n* = 1)	ATR-FTIR (*n* = 1); Raman (*n* = 1)	702.68 ± 504.26	-	-	-	^13^
	Colon (nontumoral)	N	2	Malaysia (*n* = 1); Turkey (*n* = 1)	11–30 (*n* = 2)	FTIR (*n* = 1); ATR-FTIR (*n* = 1); Raman (*n* = 1)	207.78 ± 154.12	-	-	-	^13^,^14^
	Stomach content	N	1	Turkey (*n* = 1)	11–30 (*n* = 1)	μ-Raman (*n* = 1)	9.4 ± 10.4 per individual	-	-	-	^15^
	Ileum & mesenteric fat	N	1	China (*n* = 1)	1–10 (*n* = 1)	LDIR (*n* = 1)	1.41–8.59	-	-	-	^16^
	Feces	N	14	Austria (*n* = 2); Canada (*n* = 1); Finland (*n* = 1); Germany (*n* = 1); Indonesia (*n* = 3); Japan (*n* = 1); Netherlands (*n* = 1); Poland (*n* = 1); Russia (*n* = 1); United Kingdom (*n* = 1); China (*n* = 7); Italy (*n* = 1), United Kingdom (*n* = 1)	31–50 (*n* = 1); 11–30 (*n* = 7); 1–10 (*n* = 2); 51–100 (*n* = 3); >100 (*n* = 1)	FTIR (*n* = 5); μ-Raman (*n* = 2); LDIR (*n* = 2); TD-GC/MS (*n* = 1); Py-GC/MS (*n* = 3);	1–36	-	-	3.33–345.58	^17–30^
	Gastric tumor	Y	1	China (*n* = 1)	1–10 (*n* = 1)	Py-GC/MS (*n* = 1)	-	-	-	0.008–0.109	^31^
**Respiratory**	Lung tumors	Y	1	China (*n* = 1)	11–30 (*n* = 1)	Py-GC/MS (*n* = 1)	-	-	-	0.0071–0.545	^17^
	Lung (healthy)	N	4	Brazil (*n* = 1); United Kingdom (*n* = 1); China (*n* = 2)	11–30 (*n* = 3); 51–100 (*n* = 1)	μ-FTIR (*n* = 2); LDIR (*n* = 2); Raman (*n* = 2)	0.69–2.19	-	-	-	^32–35^
	Sputum	N	4	Iran (*n* = 2); China (*n* = 2)	11–30 (*n* = 4)	LDIR (*n* = 2), FTIR (*n* = 1); μ-Raman (*n* = 2)	-	1.25–12.0	-	-	^9^,^36–38^
	Bronchoalveolar Lavage Fluid	N	8	Iran (*n* = 2); Lithunia (*n* = 1); Spain (*n* = 1); Turkey (*n* = 1); China (*n* = 3)	1–10 (*n* = 1); 11–30 (*n* = 4); 31–50 (*n* = 2); 51–100 (*n* = 1)	LDIR (*n* = 2); μ-Raman (*n* = 4); μ-FTIR (*n* = 1)	-	0.0014–0.128	-	-	^9^,^38–44^
	Nasal cavity	N	3	Republic of Korea (*n* = 1); China (*n* = 2)	31–50 (*n* = 1); 11–30 (*n* = 1); >100 (*n* = 1)	μ-FTIR (*n* = 2); LDIR (*n* = 1)	-	-	39.0	-	^37^,^45^,^46^
	Pleural fliud	N	1	Iran (*n* = 1)	1–10 (*n* = 1)	μ-Raman (*n* = 1)	-	2.1–21	-	-	^38^
	Nasopharyngeal fluid	N	1	Republic of Korea (*n* = 1)	>100 (*n* = 1)	μ-FTIR (*n* = 1)	-	-	12.9	-	^46^
**Reproductive**	Cervical (cancerous tissue)	Y	2	China (*n* = 2)	11–30 (*n* = 2)	Raman (*n* = 1); Py-GC/MS (*n* = 2)	1.87 ± 1.02	-	-	-	^31^,^47^
	Cervical (paracancerous tissue)		1	China (*n* = 1)	11–30 (*n* = 1)	Raman (*n* = 1); Py-GC/MS (*n* = 1)	-	-	-	0.0071–0.545	^47^
	Prostate tumors	Y	2	Turkey (*n* = 1); China (*n* = 1)	11–30 (*n* = 2)	ATR-FTIR (*n* = 1); LDIR (*n* = 1)	-	-	21.5 ± 10.13	181–290.3	^48^,^49^
	Penis	N	1	USA (*n* = 1)	1–10 (*n* = 1)	LDIR (*n* = 1)	-	-	-	-	^7^
	Penile tumors	Y	1	China (*n* = 1)	11–30 (*n* = 1)	LDIR (*n* = 1)	6.42	-	-	-	^50^
	Pancreatic tumors	Y	1	China (*n* = 1)	1–10 (*n* = 1)	Py-GC/MS (*n* = 1)		-	-	0.0184–0.427	^31^
	Uterine fibroids	Y	1	China (*n* = 1)	31–50 (*n* = 1)	Raman (*n* = 1)	2.13 ± 1.17	-	-	-	^51^
	Semen	N	5	Italy (*n* = 1); China (*n* = 4)	1–10 (*n* = 2); 11–30 (*n* = 1); 31–50 (*n* = 1); >100 (*n* = 1)	Raman (*n* = 3); Py-GC/MS (*n* = 2); LDIR	-	0.23 ± 0.45 to 3.57 ± 0.32	-	3.57 ± 0.32	^51–56^
	Testicular tissue	N	2	USA (*n* = 1); China (*n* = 1)	1–10 (*n* = 2)	Py-GC/MS (*n* = 2); LDIR (*n* = 1)	11.60 ± 15.52	-	-	328.44	^56^,^57^
	Placenta	N	8	Austria (*n* = 1); Canada (*n* = 1); Czech Republic (*n* = 1); Germany (*n* = 1); Iran (*n* = 1); Italy (*n* = 1); USA (*n* = 1); China (*n* = 3)	1–10 (*n* = 5); 11–30 (*n* = 1); 31–50 (*n* = 1); 51–100 (*n* = 2)	Raman (*n* = 3); FTIR (*n* = 2); LDIR (*n* = 2); Py-GC/MS (*n* = 1)	4.675–18	-	-	6.5–685	^6^,^58–64^
	Myometrium		1	China (*n* = 1)	31–50 (*n* = 1)	Raman (*n* = 1)	1.5 ± 1.17	-	-	-	^51^
	Amniotic fluid	N	3	Czech Republic (*n* = 1); China (*n* = 2)	1–10 (*n* = 2); 31–50 (*n* = 1)	Py-GC/MS (*n* = 1); LDIR (*n* = 1); FTIR (*n* = 1)	2.01 ± 4.19–4.795	-	-	-	^6^,^65^,^66^
	Ovarian follicular fluid	N	2	Italy (*n* = 1); China (*n* = 1)	1–10 (*n* = 2)	Py-GC/MS (*n* = 1)	-	2191 (range: 0–7181)	-	12.88 ± 15.54	^67^,^68^
	Meconium	N	3	Austria (*n* = 1); Germany (*n* = 1); China (*n* = 2)	1–10 (*n* = 1); 11–30 (*n* = 2)	LDIR (*n* = 2); FTIR (*n* = 2);	0–51.4	-	-	-	^60^,^63^,^69^
	Cervicovaginal lavage fluids	N	1	Republic of Korea (*n* = 1)	1–10 (*n* = 1)	Raman (*n* = 1)	0.910 ± 1.496	-	-	-	^70^
	Fetal Appendages	N	1	China (*n* = 1)	11–30 (*n* = 1)	LDIR (*n* = 1)	6.561	-	-	-	^65^
**Urinary**	Kidney	N	4	Germany (*n* = 1); Italy (*n* = 2); USA (*n* = 1)	1–10 (*n* = 3); 11–30 (*n* = 1)	Raman (*n* = 2); Py-GC/MS (*n* = 1), ATR-FTIR (*n* = 1); μ-Raman (*n* = 1)	1.2–26	-	1.7 ± 2.11	404	^8^,^71–73^
	Urine	N	8	Iran (*n* = 1); Italy (*n* = 3); United Kingdom (*n* = 1); China (*n* = 3)	1–10 (*n* = 3); 11–30 (*n* = 2); 31–50 (*n* = 1); 51–100 (*n* = 1); >100 (*n* = 1)	Raman (*n* = 3); TD-GC/MS (*n* = 1); Py-GC/MS (*n* = 1); LDIR (*n* = 1); μ-Raman (*n* = 2); μ-FTIR (*n* = 1)	-	0–4.724	-	1.5–6.49	^9^,^20^,^52^,^71^,^74–77^
**Cardiovascular**	Blood (systemic)	N	7	Netherlands (*n* = 2); Republic of Korea (*n* = 1); Turkey (*n* = 1); United Kingdom (*n* = 1); China (*n* = 2)	11–30 (*n* = 4); 31–50 (*n* = 2); 51–100 (*n* = 1)	Py-GC/MS (*n* = 4); μ-FTIR (*n* = 2); μ-Raman (*n* = 3)	-	2.4–4.2	-	0–96.2	^78–84^
	Maternal blood	N	1	China (*n* = 1)	11–30 (*n* = 1)	LDIR (*n* = 1)	-	8.176	-	-	^65^
	Umblical vein blood	N	1	China (*n* = 1)	11–30 (*n* = 1)	LDIR (*n* = 1)	2.726	-	-	-	^65^
	Arterial plaques	N	2	Italy (*n* = 1); China (*n* = 1)	1–10 (*n* = 1); >100 (*n* = 1)	Py-GC/MS (*n* = 2)	-	-	-	5.2 ± 2.4 to 156.50 ± 42.14	^85^,^86^
	Thrombi	N	2	China (*n* = 2)	11–30 (*n* = 2)	Py-GC/MS (*n* = 1); LDIR (*n* = 1); Raman (*n* = 1)	-	-	1–15	-	^10^,^12^
	Aorta, Coronary Artery, and carotid artery	N	1	China (*n* = 1)	1–10 (*n* = 1)	Py-GC/MS (*n* = 1)	-	-	-	118.66 ± 53.87	^86^
	Pericardi, Epicardial adipose tissue, Pericardial adipose tissue, Myocardia, Left atrial appendage Pre/postoperative venous blood	N	1	China (*n* = 1)	1–10 (*n* = 1)	LDIR (*n* = 1)	3–13043	-	-	-	^87^
**Nervous**	Brain	N	2	Brazil (*n* = 1); USA (*n* = 1)	11–30 (*n* = 2)	Py-GC/MS (*n* = 1); ATR-FTIR (*n* = 1); μ-FTIR (*n* = 1)	-	-	-	3345–4917	^73^,^88^
	Cerebrospinal fluid	N	1	China (*n* = 1)	11–30 (*n* = 1)	Py-GC/MS (*n* = 1)	-	-	-	0.0961–5.0243	^89^
**Hepatic**	Liver (cirrhotic)	Y	1	Germany (*n* = 1)	11–30 (*n* = 1)	Raman (*n* = 1)	1.2	-	-	-	^72^
	Liver (healthy)	N	2	Canada (*n* = 1); USA (*n* = 1)	11–30 (*n* = 1)	FTIR (*n* = 1); Py-GC/MS (*n* = 1)	-	-	-	433	^72^,^73^
	Spleen	N	1	Germany (*n* = 1)	1–10 (*n* = 1)	Raman (*n* = 1)	-	-	-	-	^72^
**Hepatobiliary**	Gallstone	N	1	China (*n* = 1)	11–30 (*n* = 1)	LDIR (*n* = 1); Py-GC/MS (*n* = 1)	5.25–10.69	-	-	0.04–10.69	^90^
**Musculoskeletal**	Bone	N	1	China (*n* = 1)	1–10 (*n* = 1)	Raman (*n* = 1)	22.9 ± 15.7	-	-	-	^91^
	Bone Marrow	N	1	China (*n* = 1)	11–30 (*n* = 1)	Py-GC/MS (*n* = 1)	-	-	-	1.75–30.02	^92^
	Cartilage	N	1	China (*n* = 1)	1–10 (*n* = 1)	Raman (*n* = 1)	26.4 ± 17.6	-	-	-	^91^
	Intervertebral disc	N	1	China (*n* = 1)	1–10 (*n* = 1)	Raman (*n* = 1)	61.1 ± 44.2	-	-	-	^91^
	Synovial fluid	N	1	Netherlands (*n* = 1)	1–10 (*n* = 1)	Raman (*n* = 1)	-	-	-	-	^93^
	Synovial tissue	N	1	China (*n* = 1)	31–50 (*n* = 1)	μ-FTIR (*n* = 1)	1.16–10.77	-	-	-	^94^
	Saphenous vein tissue	N	1	United Kingdom (*n* = 1)	1–10 (*n* = 1)	μ-FTIR (*n* = 1)	29.28 ± 34.88	-	-	-	^95^
**Integumentary**	Skin	N	2	Iran (*n* = 2)	>100 (*n* = 2)	μ-Raman (*n* = 2)	76.7 ± 15.7–165.77 ± 35.9	-	-	-	^96^,^97^
	Hair (head)	N	2	Iran (*n* = 2)	>100 (*n* = 2)	μ-Raman (*n* = 2)	-	-	3.5 per day	-	^96^,^97^
	Nasal Hair	N	1	Republic of Korea (*n* = 1)	51–100 (*n* = 1)	μ-FTIR (*n* = 1)	-	-	8.6	-	^46^
**Ocular**	Aqueous humor	N	1	China (*n* = 1)	51–100 (*n* = 1)	Py-GC/MS (*n* = 1)	-	-	-	3.7–22.3	^98^
	Vitreous humor	N	1	China (*n* = 1)	31–50 (*n* = 1)	Py-GC/MS (*n* = 1); LDIR (*n* = 1)	-	-	-	0.24–14.76	^99^
	Tear fluid/meibum	N	1	China (*n* = 1)	51–100 (*n* = 1)	Py-GC/MS (*n* = 1); LDIR (*n* = 1)	-	-	-	-	^100^
**Other**	Saliva	N	2	Iran (*n* = 2)	>100 (*n* = 2)	μ-Raman (*n* = 2)	-	0.33 per day	-	-	^96^,^97^
	Breast milk	N	2	Thailand (*n* = 1); China (*n* = 1)	1–10 (*n* = 1); 51–100 (*n* = 1)	LDIR (*n* = 1); Raman (*n* = 1)	20.2	-	1–4	-	^11^,^25^
	Human cumulus granulosa cells	N	1	China (*n* = 1)	11–30 (*n* = 1)	Py-GC/MS (*n* = 1)	-	-	-	313.14 ± 246.69	^68^
	Endometrium	N	1	China (*n* = 1)	11–30 (*n* = 1)	LDIR (*n* = 1)	0–1170	-	-	-	^101^

a *n*: denotes number of studies; FTIR: Fourier Transform Infrared Spectroscopy; ATR-FTIR: Attenuated total reflectance-FTIR; LDIR: Laser direct infrared spectroscopy; Py-GC/MS: Pyrolysis-gas chromatography-mass spectrometry; TD-GC/MS: Thermal Desorption-Gas Chromatography-Mass Spectrometry.

**Figure 1:**
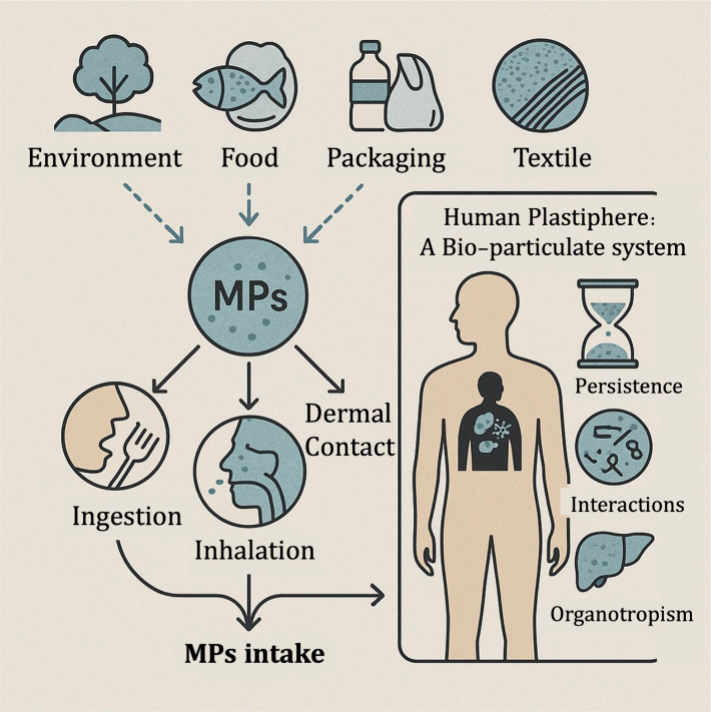
Sources and intake routes of MPs and their role in the human plastiphere. MPs originate from the environment, food, packaging, and textiles, and enter the body through ingestion, inhalation, and dermal contact. Inside the body, MPs contribute to a human plastiphere—a bioparticulate system characterized by persistence, biological interactions, and organ-specific accumulation.

Our synthesis draws on 90 biomonitoring studies published between 2016 and 2025 (Refer to Text S2 for Literature Search), which collectively report microplastics (MPs) in 63 human biological compartments, including locations once considered immunoprivileged (Table 1). Despite this growing body of evidence, major uncertainties persist regarding the pharmacokinetics, toxicological relevance, and health implications of detected particles. Many studies rely on disparate detection methods, lack mechanistic analysis, and do not investigate tissue-specific polymer retention or disease causality.^6–12^

The plastiphere framework addresses four critical gaps in current literature. First, terminological ambiguity: the term “presence” often obscures whether particles are transient or retained. Second, methodological heterogeneity: inconsistent detection platforms produce noncomparable results, frequently with limited sample sizes. Third, poorly characterized organotropism: the mechanisms behind tissue-specific accumulation remain speculative. Fourth, a disconnect between detection and functional impact: most studies lack insight into exposure-to-disease pathways. To advance beyond this impasse, we propose the plastiphere not as a metaphor for contamination, but as a systems-level construct that enables coherent interrogation of exposure, integration, and biological response. It provides a conceptual and empirical scaffold to resolve what we identify as eight core paradoxes—such as discrepancies between environmental input and tissue burden, physiological size constraint violations, unexplained particle retention, and undefined toxicity thresholds. These are not outliers but central challenges for the field.

Previous frameworks have advanced environmental and regulatory models for assessing microplastic risks, including aquatic systems and extrapolated human exposure scenarios.^102,103^ However, these frameworks generally treat microplastics as external toxicants. Here, we take a systems-level biological approach—framing microplastics as persistent, nonendogenous particles integrated into human tissues, which demand a new conceptual scaffold for risk understanding. This review therefore serves a dual purpose: to synthesize current clinical evidence on human MP exposure, and to critically map the conceptual and methodological advances needed for credible risk assessment. By framing MNPs as components of a biologically active system, the plastiphere model bridges environmental science and human physiology—providing a unified foundation for understanding synthetic particle impacts on health. Understanding this system is no longer speculative—it is essential for setting exposure thresholds, guiding policy, and addressing one of the Anthropocene’s defining health challenges: the systemic incorporation of synthetic particles into the human body and the industrialization of human physiology.

## 2 Mapping System-Wide Microplastic Accumulation and Organotropism

Our understanding of MP infiltration in human tissues has evolved from initial detection studies to comprehensive mapping of systemic distribution patterns. The MPs enter the human body through three primary exposure portals: inhalation, ingestion, and dermal contact. Each route contributes to the baseline circulating pool of particles, enabling systemic distribution. The pulmonary gateway captures airborne MPs, with lung tissue retaining 1.42 ± 1.50 particles/g of predominantly <20 μm polypropylene (PP) fibers.^34^ Bronchoalveolar lavage studies revealing 0.14–12.8 particles/100 mL indicate both continuous deposition and the respiratory system’s attempts at clearance.^42^ However, these respiratory burden estimates vary depending on the detection platform used and digestion protocols—some of which degrade synthetic fibers and exclude biologically active <3 μm particles—highlighting the urgent need for analytical harmonization.^103^

Simultaneously, the gastrointestinal tract acts as a sink for ingested MPs, with concentrations increasing along its length—from a mean of 9.4 ± 10.4 particles per person in the stomach to much higher levels in colon tumor-adjacent tissue (207.78 ± 154.12).^14,15^ While fecal excretion removes 1–36 particles/g, significant quantities evade elimination, indicating incomplete digestive clearance.^24^ Methodological discrepancies, including post-mortem redistribution, inconsistent documentation of dietary history, and variation in sample processing, challenge data interpretability.

Dermal contact, though less studied, shows consistent surface contamination. Hand swabs yield 76.7–165.8 particles/sample, and facial skin retains 100–5,000 μm polyester fibers.^96,97^ True transdermal absorption remains unconfirmed, but secondary transfer to mucosal membranes or particle fragmentation presents plausible exposure risks. Together, these routes establish a triportal foundation for plastiphere formation, sustaining a circulating reservoir of 4.2 particles/mL in blood.^83^ This reservoir facilitates systemic transport and accumulation in tissues, including sites previously considered protected.

The human plastiphere manifests through the circulatory network, enabling infiltration into protected anatomical sites and exhibiting emerging organotropism, as suggested by recurrent detection patterns across specific tissues. The reproductive system shows particular vulnerability, with testicular concentrations reaching 328.44 μg/g and semen containing 0.23 ± 0.45 particles/mL.^56,57^ Notably, follicular fluid samples from a single IVF clinic reported 2,191 particles/mL—although environmental controls were unclear, and analytical exclusion of submicron particles may underreport the actual burden.^67^ Transplacental passage is evidenced by placental accumulation of 18.0 particles/g and amniotic fluid concentrations of 2.01 ± 4.19 particles/g (20–100 μm).^66^

Neural tissues are similarly permeable, with brain MP loads increasing from 3,345 μg/g in 2016 to 4,917 μg/g in 2024.^73^ Yet these longitudinal claims are based on unmatched autopsy cohorts, confounded by lack of exposure history or analytical control for post-mortem redistribution during endarterectomy. Cerebrospinal fluid contains <100 μm fragments, but such findings are linked to albumin ratios and may reflect generalized barrier dysfunction rather than direct penetration.^88^ Ocular compartments have also shown MP presence: aqueous humor concentrations range from 3.7 to 22.3 μg/g, and tear/meibum fluid samples demonstrate the presence of PET-dominant MPs.^98^ However, pooled ocular samples, while improving detection, obscure individual variability and exclude smaller nanoplastics below analytical thresholds. These findings reinforce the notion that even sensory organs previously considered protected may not be immune to MP infiltration. Cardiovascular samples—including systemic blood, arterial plaques, thrombi, and heart tissue—consistently reveal synthetic particle presence (Table 1). Blood contains 1.6–96.2 μg/mL MPs, while coronary plaques harbor up to 141.8 μg/g, suggesting that vascular compartments act as both conduits and sinks.^12,78–84^ These findings are supported by detection in maternal and umbilical blood, further confirming systemic distribution.^65^

The hepatobiliary system (e.g., liver, gallstone) and renal compartments (e.g., kidney, urine) offer mixed findings. While cirrhotic livers show MPs, healthy livers often do not—highlighting either clearance capability or analytical limitations.^72^ Kidneys and urine reflect filtration and partial excretion pathways, but quantitative inconsistencies in excretion rates raise concerns over mass balance and potential underestimation of chronic burden. Musculoskeletal and connective tissues—including bone, bone marrow, cartilage, and synovial tissues—have revealed embedded MPs, particularly in individuals with inflammatory or degenerative conditions.^91–94^ Values in synovial tissue (e.g., 5.24 ± 2.07 particles/g) and bone (22.9 ± 15.7 particles/g) suggest long-term retention.^91,94^ However, as Table 1 shows, these results stem from isolated cohorts and may be influenced by surgical contamination, particularly from arthroplasty-derived particles that are seldom accounted for in study protocols.

Integumentary structures (e.g., skin, hair) and exocrine fluids (e.g., saliva, breast milk) also show evidence of MP presence.^11,25,96,97^ Skin retains 76.7–165.8 particles/sample, and head hair yields 3.5 MPs/day, indicating dermal persistence and potential for environmental transfer. Breast milk studies confirm maternal-offspring exposure routes. In oncological settings, a distinct oncotropism emerges: lung tumors contain 7.1–545.9 ng/g,^31^ and prostate tumors show 290.3 particles/g—possibly due to the enhanced permeability and retention (EPR; Text S1) effect.^49^ Together, Table 1 illustrates that MPs have been detected in all major organ systems and tissue types—including gastrointestinal, respiratory, cardiovascular, reproductive, nervous, hepatic, renal, musculoskeletal, integumentary, and ocular compartments—totaling over 60 distinct biological compartments. This comprehensive organotropism affirms the plastiphere as a system-wide phenomenon.

While renal filtration appears to capture 1–29 μm particles (26 particles in 10 samples) and urinary excretion removes 3–13 μm (0–3 particles/mL) MPs,^8^ reported tissue burdens across diverse organ systems suggest that accumulation may outpace elimination (Table 1). This is tentatively observed in neural tissues, where comparative data over an eight-year period show rising MP concentrations.^73^ However, analytical variation, inconsistent analytical control use, and limited sample sizes temper confidence in temporal trends (Table 1 and Table S2). These emerging patterns nonetheless suggest a complex biological phenomenon of synthetic particle retention that spans multiple physiological systems. The plastiphere does not represent a uniform contaminant load—it is a structured and evolving presence. Spatial differences in particle concentration are likely influenced not only by exposure routes but also by tissue-specific retention, biological filtering, and mechanical entrapment. This interplay may involve factors such as particle size, vascular permeability, and microanatomical affinity.

Significant gaps remain in our understanding of excretion pathways (e.g., biliary, mammary), lymphatic transport, endocrine tissue retention, and mucosal clearance. These underexplored systems are likely critical to understanding MNP pharmacokinetics. Until these routes are better characterized, the mechanisms behind tissue-level accumulation will remain uncertain. Current findings suggest that MNPs interact with biological structures in nonrandom ways—behaving less like passive contaminants and more like persistent, bioparticulate agents influenced by internal anatomy.

## 3 Limitations Undermining Plastiphere Research

The expanding body of human plastiphere research is shaped by three foundational limitations that restrict the validity, interpretability, and generalizability of its findings. These constraints not only compromise reproducibility but also hinder the development of robust exposure-risk models and mechanistic insight.

### 3.1 Sampling Frameworks and Comparative Group Deficits

Human plastiphere research is shaped by striking demographic, geographic, and design-level limitations. Geographically, 43.4% of studies are conducted in China, with sparse representation from Africa, South America, and low-income regions (Figure 2a). This skew limits global relevance and neglects populations disproportionately burdened by plastic exposure. Demographically, most studies focus on adults (73.3%), while children and newborns remain largely excluded (Figure 2b). Gender representation is uneven—56% male versus 44% female participants (Figure 2c)—and nearly half of all studies (47.5%) sample only individuals with existing health conditions such as cancer or metabolic disorders, raising concerns about disease-related confounding (Figure 2d). Only 32% explicitly include workers with known exposure risk, while 25.3% omit occupational data altogether (Figure 2e). Most studies are underpowered: 64.7% use cohorts smaller than 30, and only 10.2% exceed 100 participants (Figure 2f). Such limited samples reduce statistical robustness and increase vulnerability to outlier effects, especially in tissue-specific analyses.^78^ Without power calculations and multicenter designs, the field remains blind to the scope and distributional logic of plastiphere accumulation.

**Figure 2:**
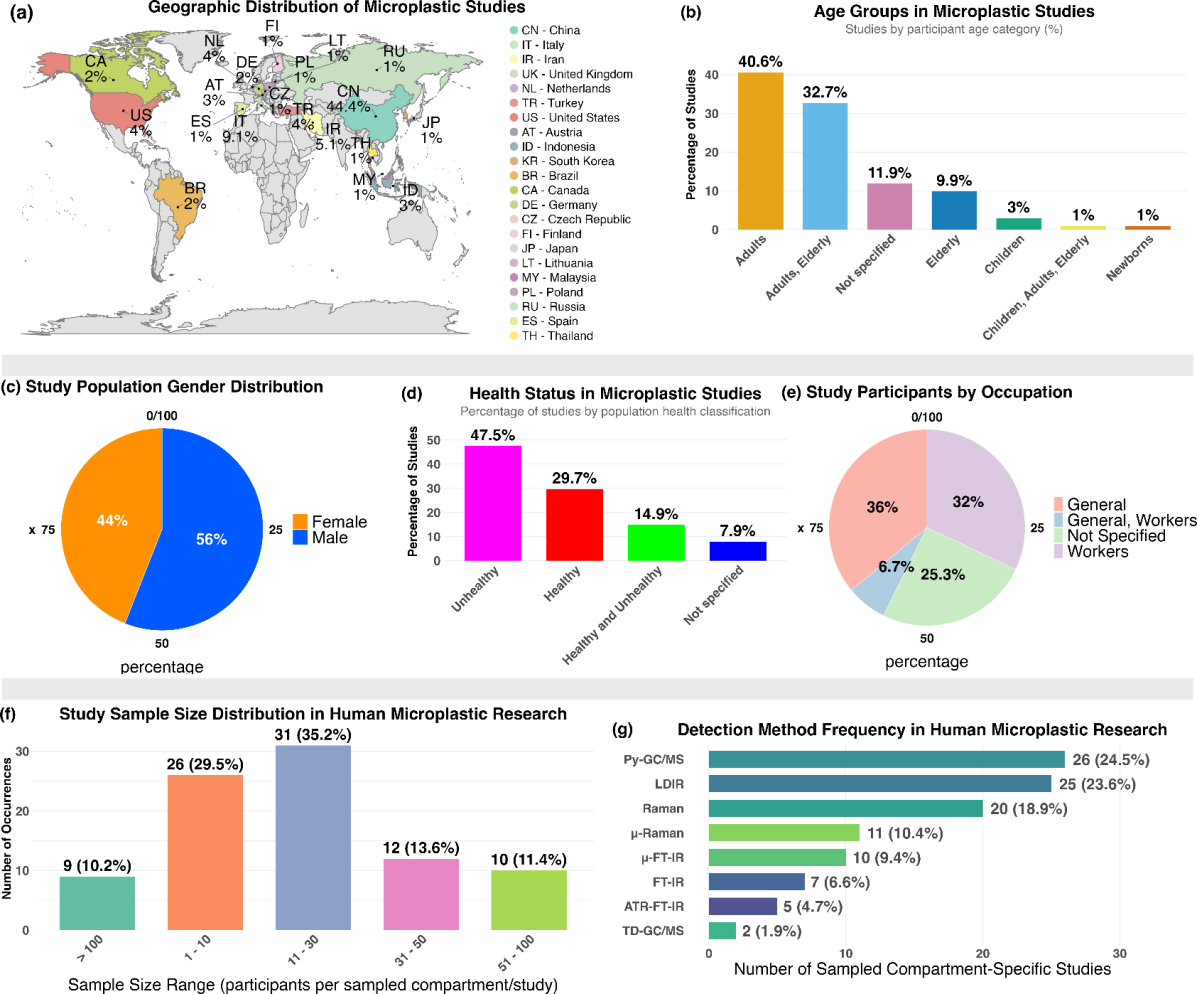
Demographic and methodological characterization of human microplastic exposure studies. (a) Geographic distribution of studies reporting human microplastic detection by country. (b) Age groups assessed in human microplastic exposure studies, highlighting a predominance of adult-only samples. (c) Gender representation across all studies, showing a modest male bias. (d) Health status of studied populations, with nearly half of studies focused on individuals with underlying conditions. (e) Occupational groups included in studies, with a large proportion not reporting occupational background. (f) Distribution of study sample sizes per sampled compartment, with most studies involving fewer than 30 participants. (g) Analytical detection methods used across human tissue microplastic studies, showing frequent use of Py-GCMS and LDIR.

Beyond sampling, studies frequently lack rigorously matched comparative groups. Adjacent or para-tumoral tissues are often used as proxies for “normal,” despite shared exposure histories and inflammatory microenvironments.^49^ Others rely on unmatched cadavers or convenience cohorts with undefined environmental or occupational exposures. Longitudinal autopsy data are sometimes used to infer accumulation trends, but without accounting for life-course exposure variability or post-mortem redistribution.^15,73^ Together, these limitations obscure population-level risks, distort accumulation patterns, and perpetuate environmental health inequities. To enhance interpretability, future plastiphere studies should aim for cohort sizes of at least 50–100 participants, stratified by age, sex, and exposure type. Broadening both demographic and geographic scope is essential to build representative exposure models and credible biomonitoring strategies.

### 3.2 Source–Exposure Attribution Gaps

Efforts to link external MP exposures to internal tissue accumulation remain methodologically underpowered and conceptually overstated. Despite widespread exposure via ingestion, inhalation, and dermal contact, the pathways through which specific sources contribute to tissue-level burdens remain poorly resolved. Bottled water, seafood, and processed foods are frequent MP carriers, often contaminated with hundreds to hundreds of thousands of particles per kilogram or liter.^104–109^ Yet the connections between dietary habits and internal plastiphere burden remain largely correlative. Studies correlating seafood consumption or bottled beverage intake with placental, fecal, or reproductive MP loads often overlook key variables—such as interbatch variability in MP content, particle-specific absorption kinetics, or the differential uptake of polymer types.^19,22,24,25,52,66,110^ Occupational exposure research frequently neglects background sources like household dust or synthetic textiles, while urban–rural comparisons rarely control for ubiquitous indoor environments.^96,97^

Attribution efforts rely heavily on simple polymer matches, lacking the forensic resolution of isotopic tracing or additive profiling. Food preparation methods—like microwaving in plastic containers—are seldom quantified, despite their potential to release high MP loads.^111–113^ Without real-time exposure monitoring, uptake studies, or source-specific burden validation, claims linking behaviors to internal accumulation remain speculative. A robust framework linking exposure intensity, polymer identity, and tissue fate is essential for converting correlation into causation and for accurately modeling the drivers of plastiphere formation.

### 3.3 Analytical Discrepancies and Data Interpretation Limits

Methodological inconsistencies continue to undermine comparability across plastiphere studies. Sampling volumes range from <5 g (most organs) to ∼100 g (placenta),^61,62^ yet concentrations are normalized identically, skewing cross-tissue interpretation. Only 63.7% of studies report a numerical LOD (Limit of Detection)—often tied to filter thresholds or equipment size limits, with few including calibration standard runs—while 31.9% omit it entirely and 4.4% provide only vague descriptors (Table S2). Detection methods vary widely: pyrolysis-GC/MS (24.5%) and LDIR (23.6%) dominate, followed by Raman (18.9%) and μ-Raman (10.4%), each with distinct outputs—mass vs particle count (Figure 2g). Despite this, 80% of studies rely on a single platform, rarely reconciling methodological differences. Units are inconsistently reported (e.g., μg/mL, particles/g, % polymer), complicating synthesis. While 68.1% of studies include blanks, contamination controls are often inadequately described (Table S2). Recent critiques have further highlighted these concerns, noting issues of contamination control and method-specific biases, particularly in Py-GC/MS-based studies.^114,115^ Critical metadata—such as recovery rates, digestion efficiency, or calibration protocols—are frequently missing, further obscuring interpretation. These inconsistencies distort both intra- and interorgan comparisons, risking spurious conclusions about tissue burden and retention. Without harmonized standards for reporting LODs, units, and metadata, the field lacks the rigor needed for reliable mechanistic insight. Analytical pluralism without methodological transparency turns complexity into opacity—standardization is no longer optional, but essential.

## 4 Correlation without Causation? Health Outcomes and the Human Plastiphere

The relationship between plastiphere accumulation and human health outcomes presents a complex interplay of statistical correlation and mechanistic uncertainty. A growing number of studies report significant associations between MP presence and disease states across organ systems. However, these correlations must be interpreted with caution given widespread methodological limitations, unaccounted confounders, and a near-total absence of longitudinal data. This section synthesizes current evidence across key physiological systems—including cardiovascular, reproductive, gastrointestinal, respiratory, and neurological domains—while identifying unresolved challenges that obstruct causal inference.

### 4.1 Cardiovascular Disease and Microplastics

Among the most provocative findings is the reported link between MPs and cardiovascular disease. Marfella et al. observed a 4.5-fold increased risk of myocardial infarction or stroke among patients with polyethylene-containing carotid artery plaques over a 34-month period.^85^ This association persisted after adjustment for conventional risk factors, suggesting MPs may function as an independent contributor to vascular pathology. Corroborating evidence comes from thrombi containing pigment-loaded MPs that strongly correlate with markers of platelet activation.^10^ Yet critical uncertainties remain: do MPs initiate plaque formation, or are they merely deposited into existing lesions? Furthermore, coexposure to airborne MPs—particularly from tire wear or urban dust—is rarely quantified, complicating exposure attribution and raising the potential for misclassification.

### 4.2 Reproductive Health Consequences

Reproductive studies offer some of the clearest dose–response patterns in plastiphere research. In semen, each additional MP polymer type is associated with an 8.3% reduction in progressive sperm motility, with polytetrafluoroethylene showing the strongest negative impact.^52^ Female reproductive findings echo this trend, with follicular fluid MP concentrations correlated with altered FSH levels.^53^ These associations are biologically plausible, supported by in vivo evidence of endocrine disruption and gametogenic toxicity.^116–118^ However, most studies rely on single time point sampling and lack controls for coexposure to phthalates, bisphenols, or other plastic additives—limiting interpretability and obscuring mechanistic resolution.

### 4.3 Metabolic and Gastrointestinal Effects

Associations between MP exposure and metabolic dysfunction are more ambiguous. Inflammatory bowel disease studies report nearly 50% higher fecal MP loads in affected individuals compared to healthy controls,^19^ and creeping fat in Crohn’s disease patients harbors MP levels six times higher than adjacent tissue.^16^ These findings raise the possibility that MPs contribute to inflammatory modulation, but directionality is unclear—chronic inflammation itself may alter MP absorption, distribution, or retention. Diet adds a further layer of confounding: individuals with higher processed food intake tend to show higher MP levels and metabolic risk, but disentangling cause from cocorrelation remains difficult.^18^ Gut microbiome studies provide additional mechanistic clues: MP exposure is associated with depletion of beneficial species like *Faecalibacterium prausnitzii* and enrichment of pro-inflammatory taxa such as *Enterobacteriaceae*.^23,29^ In children, poly­(vinyl chloride) (PVC) and PET levels in stool correlate inversely with microbial diversity,^22^ while MASLD (Metabolic Dysfunction-Associated Steatotic Liver Disease) cohorts show polymer-specific shifts in *Bifidobacterium* and *Lachnospiraceae*.^18^ Whether these microbial shifts represent mediators or consequences of MP exposure remains to be determined.

### 4.4 Respiratory Health Outcomes

Respiratory findings offer some of the most consistent exposure–response gradients, particularly in high-exposure occupational settings. Workers in plastic manufacturing facilities exhibit markedly elevated dermal and inhalational MP burdens,^96^ along with increased respiratory symptoms. Urban populations similarly show higher lung MP loads than rural counterparts, while a pediatric BALF study have detected MPs in children hospitalized with severe pneumonia.^39^ Yet interpretation remains limited by the absence of preinfection baselines and by small sample sizes. Experimental data lend biological plausibility, with animal models showing MP-induced lung inflammation,^119–121^ but human studies remain largely cross-sectional and correlative.

### 4.5 Musculoskeletal and Ocular Systems

Evidence linking MPs to musculoskeletal pathology is preliminary but intriguing. MP concentrations in bone tissue from patients with osteoporosis (22.9 ± 15.7 particles/g) correlate with inflammatory markers such as TNF-α (*r* = 0.71), though industrial exposure was not assessed in this cohort.^91^ Synovial fluid analyses show MP accumulation in arthritis patients, but these studies universally lack baseline measurements, often exclude particles <10 μm, and fail to account for potential contamination from joint prostheses.^94^ Ocular studies report PET-dominant MPs in aqueous humor (3.7–22.3 μg/g) across age groups, with some links to retinal pathologies and dry eye symptoms.^100^ However, these findings are based on small, cross-sectional cohorts and lack longitudinal data necessary to assess progression or causality.

### 4.6 Incorporating Animal Models for Causality

Epidemiological studies consistently link MP exposure to various health outcomes, but establishing definitive causation is challenging due to limitations in human studies, such as uncontrolled environmental exposures, long disease latency, and confounding factors. Animal models are essential for bridging this gap, providing controlled conditions to investigate causal mechanisms. Rodent studies have confirmed that MPs induce oxidative stress, trigger inflammation, and disrupt endothelial function, while also establishing dose–response relationships, particularly for nanoplastics, whose enhanced tissue penetration and surface reactivity may amplify pathological effects and immune responses.^122–128^ However, translating these findings to humans is complicated by interspecies differences in particle metabolism^128,129^ and the absence of validated human biomarkers.^130–133^

Despite these challenges, evidence suggests MPs preferentially disrupt barrier tissues in the lungs, liver, spleen, and gastrointestinal tract, leading to chronic damage.^120,127,133–135^ Reproductive studies in animals show altered hormone levels, reduced fertility, and organ dysfunction, with nanoplastics raising concerns about autoimmune activation and inflammation.^136–139^ MPs’ health implications extend beyond particles themselves. Plastic additives, like phthalates and bisphenols, leach into biological systems, while MP surfaces act as vectors for environmental pollutants.^140,141^ MPs may also disrupt the human microbiome, leading to dysbiosis and pro-inflammatory taxa.^142,143^ Animal and in vitro models indicate that MPs deplete beneficial species like *Faecalibacterium prausnitzii*, exacerbating inflammation and potentially influencing immune function.^144,145^ MNP exposure may also disrupt the gut-liver and gut-brain axes, influencing metabolic and neurological health.^146–150^

While human-specific data is limited, integrating animal models, in vitro studies, and biomonitoring can help bridge this gap. Emerging techniques like mass spectrometry imaging, organ-on-chip systems, and exposome-wide analyses offer promising tools to investigate MP-related disease progression.^151–153^ Until causal pathways are confirmed, the biological plausibility of MP-mediated harm warrants precautionary concern.

## 5 The Eight Paradoxes: Where Our Understanding Breaks Down

Despite mounting evidence of MP presence in human tissues, foundational contradictions remain unresolved. This section outlines eight paradoxes—analytical, biological, and chronological—that fracture our understanding of exposure, accumulation, and risk. As shown in Figure 3, each paradox is paired with a proposed resolution pathway. These contradictions are not anomalies, but signs of a deeper epistemological problem: how fragmented methods and assumptions shape what we know—and what we miss—about the plastiphere. These paradoxes reveal the plastiphere’s dual behavior: it obeys biological distribution laws (e.g., vascular transport) while defying environmental particle fate models (e.g., incomplete excretion).

**Figure 3:**
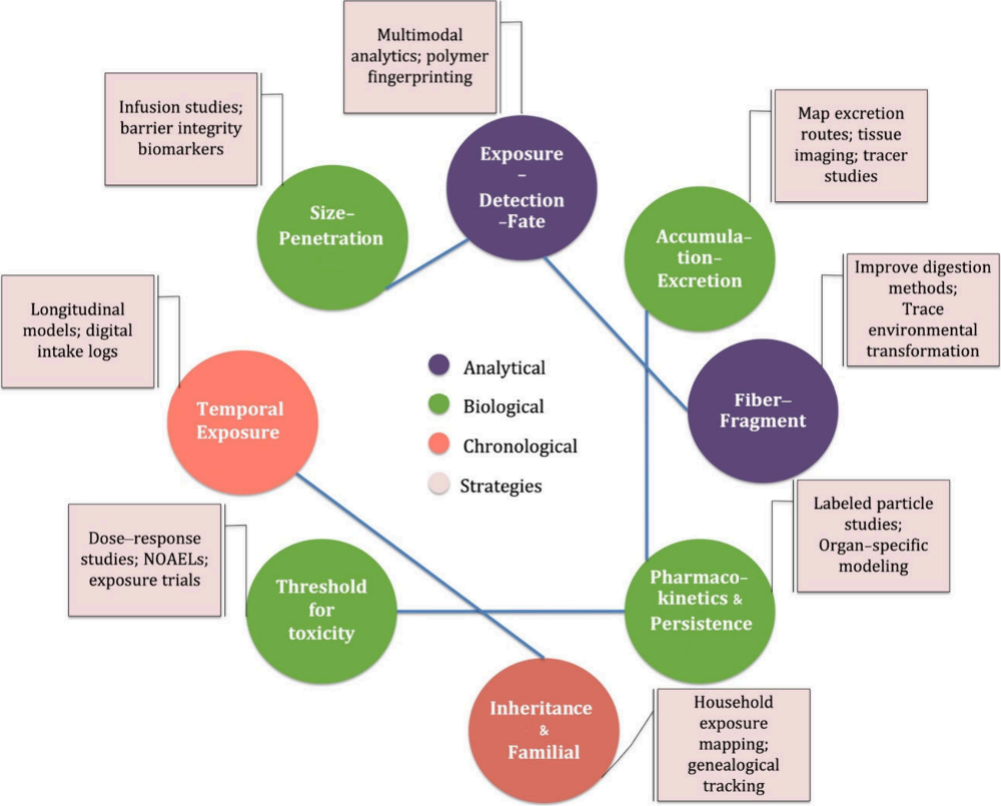
Domains of the eight plastiphere paradoxes. Node colors represent the domain of each paradox—Analytical (blue), Biological (green), and Chronological (red)—while linked boxes propose domain-specific strategies to resolve each paradox.

### 5.1 Threshold for Toxicity Paradox

At what burden does the presence of synthetic particles in human tissues translate into measurable pathology? This unresolved question lies at the heart of the threshold paradox—one of the most critical gaps in plastiphere science. Despite increasing evidence of MP presence in tissues, no concentration thresholds have been established to distinguish benign exposure from biologically significant harm. This absence presents a major obstacle to credible risk assessment.

Three core limitations define this paradox. First, there are no mechanistic toxicity thresholds linking particle concentration to specific biological outcomes. For example, 21.7 μg PE/mg in arterial plaques remains contextless: Is this indicative of passive accumulation, subclinical stress, or active disease? No study has identified the concentration points that trigger oxidative stress, immune dysregulation, or disease severity, clinical correlation curves connecting exposure to disease severity, or time-dependent profiles separating acute from cumulative toxicity.

Second, a pervasive fallacy persists: analytical presence is not pathology. Studies often equate detection with biological relevance, ignoring confounders like comigrating additives or pre-existing tissue damage that may facilitate particle retention. There is no standardized framework to differentiate between: (1) silent accumulation, (2) early subclinical engagement, and (3) active pathological transformation.

Third, this uncertainty leads to regulatory paralysis. Without validated toxicity thresholds, dose–response models, or reliable biomarkers of harm, policymakers cannot assess risk or implement safety standards. Consequently, synthetic particle exposure exists in a scientific and regulatory limbo—ubiquitous but unclassified.

To resolve this paradox, we propose a three-pronged agenda:

1. Toxicodynamic Modeling: Establish polymer- and size-specific dose–response relationships.
2. Clinical Correlation Standards: Enforce strict evidentiary criteria for pathology claims.
3. Threshold-Finding Studies: Conduct longitudinal biospecimen monitoring and retrospective exposure analyses using archived tissues or noninvasive biomarkers.

The establishment of NOAELs for different microplastic types and exposure routes should become a priority research target. Resolving this paradox could shift the field from speculative detection to actionable public health guidance.

### 5.2 Accumulation-Excretion Imbalance Paradox

Plastiphere research faces a key contradiction: daily exposure estimates far exceed the quantities eliminated through known excretory pathways. Daily human intake of microplastics—via ingestion, inhalation, and potentially dermal contact—is estimated to reach millions of particles. Yet excretory outputs, including fecal and urinary elimination, fall far short of this volume, and tissue concentrations remain modest in most anatomical sites (Table 1).^154^ Respiratory clearance appears similarly inefficient: bronchoalveolar lavage fluid removes <2% of estimated inhaled particles, while pulmonary tissue still retains detectable loads.^42,155^ Circulating MPs in blood (∼21,000 particles) reinforce the idea of systemic persistence, despite ongoing exposure.^83^

To reconcile these discrepancies, two models have been proposed. The Dynamic Equilibrium Model suggests a persistent circulating pool, balanced by continuous low-level intake and under-recognized elimination pathways—such as hepatobiliary secretion, dermal shedding, or mucosal excretion. While appealing, this model struggles to explain long-term retention in immune-privileged or poorly perfused tissues. The Progressive Sequestration Model, by contrast, assumes that a small but biologically significant fraction of MPs evade clearance and become permanently incorporated. This aligns with reports of MPs in synovial fluid,^93^ vascular plaques,^85,86^ bone,^91^ and reproductive tissues,^58–64^ but fails to account for the untraced majority of exposure.

These mechanistic models underpin competing health hypotheses. The Cumulative Damage Hypothesis posits that sustained low-level accumulation drives systemic inflammation and tissue remodeling. The Hotspot Pathology Hypothesis argues for localized retention leading to organ-specific dysfunction. Both demand empirical validation, but neither resolves the fate of the “missing mass” of ingested or inhaled particles. Resolving this paradox requires new experimental approaches: tracer-enabled fate mapping, real-time excretion tracking, and quantitative thresholds for long-term retention. Until then, estimates of exposure and harm remain disconnected—limiting the predictive power of current plastiphere risk models.

### 5.3 Exposure-Detection-Fate Paradox

Despite widespread environmental exposure, human tissue studies consistently detect only a limited set of polymers—PE, PP, PS, PET, and PVC—highlighting a disconnect between environmental diversity and biological detectability.^21,22,52,68^ This skew is analytical, not biological: most platforms miss nanoplastics (<1 μm), and digestion protocols degrade labile polymers.^156,157^ Polyolefins dominate not due to biological affinity but because they persist and are more easily detected. Detection methods further distort interpretation. LDIR and Raman identify a wide range of polymers but offer poor quantification, while pyrolysis-GC/MS give precise mass data and consistently yields 3–5× higher concentrations than FTIR for fewer targets.^158,159^ Mixed units (Table 1) and inconsistent calibration compound the issue, obscuring exposure trends and undermining mechanistic insights. Current models of risk and retention—like organotropism or damage accumulation—are built on incomplete polymer and particle profiles, skewing understanding of plastic fate and effects. Frameworks such as the Cumulative Damage hypothesis or Hotspot model assume accurate particle mapping, yet undetected nanoplastics may transit, transform, or excrete unseen. Current analytical methods are thus incompatible, making it difficult to link environmental exposure with tissue burden. Resolving this requires multimodal, nanoparticle-sensitive workflows. Until standard tools evolve, our view of the plastiphere will reflect instrumental limits more than physiological reality.

### 5.4 Size-Penetration Paradox

The physical size of micro- and nanoplastics is widely assumed to dictate their ability to cross biological barriers, yet this relationship remains paradoxical. Conventional toxicological frameworks suggest that only particles <150 nm can traverse cellular membranes, and that those >1 μm are excluded from privileged compartments such as the brain, placenta, or gastrointestinal mucosa (Table S2).^160,161^ However, human studies routinely report MPs sized 5–50 μm in tissues previously considered impermeable, including amniotic fluid, follicular fluid, brain, and placenta (Table S1).^64,67,66,88^

Three overlapping explanations may account for this mismatch. First, physiological barriers are often studied in vitro or under homeostatic conditions,^127,162–164^ overlooking inflammatory states or disease-induced permeability changes. Second, particle shape, agglomeration, and surface properties may enable larger MPs to pass through tight junctions or undergo endocytic transport.^165^ Third, current tools under-detect nanoparticles, potentially masking their biological contribution while overemphasizing visible fragments. Rodent models have confirmed that 5 μm MPs can persist in liver and gut tissues for weeks, suggesting filtration thresholds may be more dynamic than assumed.^124,166^ These findings underscore that size alone cannot explain penetration potential—factors such as protein corona formation, mucosal adhesion, and paracellular leakage must also be considered.^167–169^

Existing mechanisms like transcytosis or paracellular transport remain largely unvalidated at these scales, and experimental models that replicate both particle morphology and physiological barrier conditions are scarce. Current findings suggest MPs may exploit noncanonical transport routes or reflect an underrecognized form of barrier plasticity. Until these gaps are resolved, the size–penetration paradox remains a key obstacle to validating the plastiphere as a functional biological system. The presence of large synthetic particles in protected compartments demands both cautious interpretation and robust empirical follow-up. Resolving this paradox will be central to moving from detection to causation—and from hypothesis to certainty—in the emerging biology of human–plastic interactions.

### 5.5 Temporal Exposure Paradox

The Temporal Exposure Paradox arises from the disconnect between short-term exposure measurements and the persistence of MPs in human tissues. Current exposure studies largely rely on 3–7 day dietary recalls or environmental sampling snapshots,^17,30^ yet post-mortem analyses suggest cumulative exposure over years.^73^ This disparity hinders linking immediate behaviors to long-term biological effects. This paradox manifests in two main areas: (a) Retention vs clearance: Studies show that 85–90% of ingested MPs are excreted within 5 days,^170^ yet brain tissues display rising MP concentrations, with a 47% increase from 2016 to 2024.^73^ (b) Vascular plaque deposition: MP burdens in plaques suggest sustained exposure,^85^ but no environmental or dietary data set has predicted such chronic deposition.

Methodologically, reliance on single-time point biospecimen collection (in 91% of studies) and the absence of longitudinal tracking make it impossible to distinguish between transient passage and true bioaccumulation. To resolve this paradox, we propose: (1) Temporal reconstruction using biological archives like tooth enamel and adipose biopsies. (2) Dual-matrix designs, pairing tissue analysis with real-time excreta sampling and polymer fingerprinting to connect internal loads with exposure sources. (3) Physiologically based pharmacokinetic (PBPK) modeling to link retention patterns to clearance dynamics. Modernizing 7-day dietary logs with digital tracking and large cohort sizes will help capture intake variability and improve retention models. Until then, plastiphere research remains caught between acute exposure assessments and chronic accumulation outcomes.

### 5.6 Fiber-Fragment Paradox

A striking morphological paradox characterizes microplastic research: while environmental studies consistently report fibers as the dominant form—comprising 67–92% of airborne and aquatic MPs^171,172^ —human tissues overwhelmingly contain fragments (Table S1). Lung biopsies report 48% fragments,^34^ while placental and intestinal samples approach or exceed 95–100%.^59,67,71,74,91^ This inversion persists despite well-documented fiber ingestion from food and salt.^173–176^ Three nonexclusive explanations may underlie this disconnect: (1) the biological exclusion of long fibers via mucociliary or gastrointestinal clearance,^177–179^ (2) selective degradation of fibers during tissue digestion,^156,157^ and (3) overlooked fragment sources, such as processed foods and bottled beverages, which can release up to 10^4^ PE/PP fragments per liter.^105^

Importantly, this paradox signals more than analytical inconsistency—it may reflect a transformation from environmental input to biological incorporation. If tissue MP profiles result from selective retention and structural filtering, the plastiphere is not a passive mirror of ambient exposure but a biologically sculpted subset. This has profound implications for exposure modeling and risk assessment, suggesting that ambient monitoring may poorly predict tissue-level burden. Addressing the paradox will require methodologically harmonized studies capable of preserving fiber integrity, and controlled tracer experiments that follow particles from intake to tissue retention. Until then, this paradox remains a key expression of the field’s unresolved translation gap between external exposure and internal reality.

### 5.7 Pharmacokinetics and Tissue Persistence Paradox

A major unresolved question in plastiphere research is the long-term fate of MPs within human tissues. While evidence of MPs in various organs is growing (Table 1), there is currently no data on MP pharmacokinetics in humans. This raises a fundamental issue: does their detection indicate bioaccumulation, ongoing exposure, or slow clearance? Rodent studies provide some insight. For instance, 15% of 5 μm polystyrene particles remained in liver tissue after 28 days, suggesting a human half-life of 3–4 months, with some particles possibly persisting for over a year. However, rodent models have limitations: human metabolism differs, and factors such as particle composition, coating, and size affect retention.

Environmental degradation rates, such as oxidized polyethylene losing 60% of its tensile strength over 35 years,^180^ suggest long-term persistence. However, biological systems differ, engaging particles through immune surveillance and enzymatic breakdown. Additionally, factors like age, health status, and gender may influence MP retention, with chronic low-dose exposure leading to gradual accumulation in tissues over time.

Key questions remain about whether different polymers like PET and PE are metabolized or excreted differently, and how persistence varies between dynamic (e.g., liver, spleen) and static (e.g., bone, brain) tissues. No studies have tracked long-term MP retention in humans, and ethical limitations prevent direct labeling experiments. To resolve this paradox, pharmacokinetic modeling combined with multifaceted tissue analysis and biomarker studies is essential. Until then, bioaccumulation claims must distinguish persistent detection from proven retention.

### 5.8 Inheritance and Familial Exposure Paradox

The inheritance paradox arises from familial clustering of disease, which may reflect shared environmental exposure rather than true germline transmission. Microplastics have been found in placental tissues and neonatal meconium,^58–63^ but no human data confirm inherited MP-related pathologies or direct germline effects. Households often exhibit persistent MP contamination via water, air, food, and consumer products. These exposures can persist across generations without implying genetic transmission, particularly in the absence of biomarkers to differentiate inherited vulnerability from environmental exposure. Familial clustering should therefore be treated as hypothesis-generating rather than evidence of inheritance. Although no human studies confirm heritable MP-related pathologies, in vivo studies in mice and *C. elegans* indicate that parental MP exposure can impair offspring development, reproduction, and neurobehavior—mediated by oxidative stress, inflammation, and epigenetic alterations.^181–186^ These findings suggest plausible intergenerational effects that merit investigation in human cohorts.

## 6 Scientific Bottlenecks and Policy Stalemates

The detection of MPs in human tissues has outpaced our ability to assess risks or implement effective policies. Three interconnected barriers—exposure uncertainty, methodological limitations, and regulatory inertia—persist due to unresolved paradoxes in plastiphere science. Below, we outline targeted strategies to transform these challenges into actionable solutions.

### 6.1 Pragmatic Exposure Reduction Steps Amid Uncertainty

Although full scientific certainty is lacking, immediate action is necessary to reduce MP exposure. The Threshold for Toxicity Paradox (Section 5.6) highlights the absence of safety benchmarks, but precautionary measures can target high-risk pathways. For instance, banning PVC and polystyrene in food packaging addresses the Fiber-Fragment Paradox (Section 5.1) by reducing fragment generation. Mandatory respirators in plastic manufacturing help mitigate inhalation risks from larger MPs, addressing the Size-Penetration Paradox (Section 5.4). Public health advisories should prioritize vulnerable groups—children and pregnant women—who are especially susceptible due to early life accumulation (Temporal Exposure Paradox). Additionally, community-level biomonitoring (e.g., fecal MP testing) can identify hotspots while circumventing the Detection-Dissonance Paradox. Product labeling reforms and a simplified Migration Index can empower consumers, providing information about potential risks while awaiting full analytical standardization.

### 6.2 Longitudinal Human Exposure Studies and Alternative Models for Particle Size Mismatch

Current experimental models often rely on nanoscale or sub-10 μm particles,^187–189^ which do not reflect the larger MPs found in human tissues. This discrepancy creates uncertainties in interpreting toxicity and biological effects. While recent models using particles in the 10–150 μm range show relevant findings,^190–192^ the field needs size-matched, polymer-specific models to better align with real-world exposures. Though longitudinal tissue biopsies may be unfeasible, alternative designs can still provide meaningful data. Repeat-sampling cohorts over 5–10 years could track temporal trends in polymer-specific MP concentrations in biofluids (e.g., blood, urine, stool) and link them to biomarkers of oxidative stress, inflammation, immune dysfunction, or endocrine disruption. Cohorts stratified by high-risk groups (e.g., children, elderly, or occupationally exposed workers) or geographic variability could enhance signal detection and help identify thresholds for biological effects. Pregnancy/birth cohorts could assess maternal–fetal transfer and track neurodevelopmental or immune-related outcomes in children, while also correlating prenatal MP levels with later-life health effects to infer critical exposure windows.

Additionally, biobanked tissues linked with clinical histories could retrospectively model exposure–disease relationships for conditions like cardiovascular disease, neurodegeneration, or infertility, with dose–response analyses stratified by disease severity. Sentinel populations (e.g., wastewater workers, plastic industry employees, or coastal communities) with high exposure gradients could serve as natural contrast groups, circumventing the challenge of finding ‘pure’ unexposed controls, while providing real-world data to validate lab-derived dose–response curves. While these approaches may not resolve all mechanistic gaps, they would significantly improve exposure assessment, quantify exposure-risk gradients, and causal inference strength.

### 6.3 Biomarker Validation in Plastiphere Research

Human plastiphere research faces significant challenges due to the lack of microplastic-specific biomarkers and dose–response thresholds. Existing biomarkers such as S100B (blood–brain barrier), IL-8 (respiratory inflammation), and ROS (reproductive stress) show promise but lack validation for microplastics, hindering their use in risk assessment and regulatory action. A tiered biomarker system is urgently needed to bridge these gaps. This system should include: (1) exposure markers (e.g., urinary polymer metabolites), (2) effect markers (e.g., VCAM-1 for vascular inflammation), and (3) susceptibility indicators (e.g., genetic variants in clearance pathways). In this context, Table 2 presents candidate biomarkers across exposure, effect, and susceptibility domains to provide a foundation for validation. Additionally, transcriptomic profiling could help identify plastic-induced expression patterns that inform both mechanistic insights and biomarker refinement. However, without harmonized protocols and validated biomarkers, current detection data cannot be translated into effective regulatory actions, keeping the field stalled under the Detection-Dissonance Paradox.

**Table 2:** Biomarkers for Assessing MNP-Linked Health Effects

Health Impact Area	Key Biomarkers & Assessment Methods
Metabolic & Endocrine Disruption	Insulin resistance markers (HOMA-IR, HbA1c, C-peptide), adipokines (leptin, adiponectin), thyroid hormone levels (T3/T4 ratio).
Neurological Effects	Blood-brain barrier integrity markers (S100B, occludin, GFAP), neuroinflammatory cytokines (IL-6, TNF-α, IL-10, IL-18), oxidative stress markers (8-OHdG, MDA), neuroplasticity marker (BDNF).
Gastrointestinal & Microbiota Disruption	Gut microbiome profiling (16S rRNA sequencing), fecal inflammatory markers (calprotectin, zonulin, lipopolysaccharide (LPS)), short-chain fatty acids (SCFAs) (microbiome metabolic activity).
Respiratory & Pulmonary Risks	Bronchoalveolar lavage (BAL) analysis, exhaled nitric oxide, lung inflammatory cytokines (IL-8, IL-1β), SP-D (lung epithelial injury marker), MMP-9 (tissue remodeling marker).
Reproductive & Developmental Toxicity	Sperm motility and morphology, oxidative stress markers in sperm (ROS, TAC - Total Antioxidant Capacity), ovarian reserve marker (anti-Müllerian hormone, AMH), reproductive hormone balance (FSH, LH, progesterone/estradiol ratio), placental inflammatory markers.
Carcinogenic Potential	DNA damage markers (γ-H2AX, comet assay, 8-oxo-dG, TP53 mutations), chronic inflammatory markers (CRP, IL-1β), tissue-specific MNP burden.
Cardiovascular Effects	Endothelial dysfunction markers (VCAM-1, ICAM-1, sICAM-1/sVCAM-1), platelet activation (P-selectin), clotting factors (D-dimer, fibrinogen), hs-CRP (chronic inflammation), homocysteine (vascular stress marker).
Urinary Disorders	Renal function markers (creatinine, urea, urinary protein excretion), oxidative stress in kidney tissue, NAG (proximal tubular damage), KIM-1 (early renal injury), urinary microalbumin (glomerular filtration issue).

### 6.4 Actionable Policy Levers

To break the deadlock, policies must address key paradoxes in plastiphere research. First, mandatory industry investment in analytical standardization (e.g., ISO protocols for nanoplastics) would resolve the Exposure-Detection-Fate Paradox, enabling comparable data. Second, national biomonitoring programs (e.g., US NHANES) should track temporal accumulation and tissue-specific burdens (e.g., placental MPs), addressing the Temporal and Size-Penetration Paradoxes. Third, presumptive liability should shift the burden of proof to manufacturers, tackling the Threshold Paradox by requiring safety data for high-production-volume polymers.

Industry claims of “low risk” exploit gaps like the absence of NOAELs, leaving regulators unable to enforce robust risk assessments. Existing initiatives show feasibility: the EU’s ban on intentional microplastics (Regulation 2023/2055), France’s mandatory microfiber filters (2025), California’s Microbead-Free Waters Act (2015), Canada’s CEPA classification of plastics as toxic, and Japan’s microbead ban demonstrate science-guided regulation despite uncertainty.^193–197^ These examples align with various paradoxes identified in this review—from fiber–fragment exposure to uncertain chemical thresholds—and highlight how science-guided regulation can proceed even amid uncertainty.

The UN Global Plastics Treaty should formalize these approaches—potentially mandating particle-size reporting and allocating funds for cohort studies to resolve the Inheritance Paradox. Aligning policies with paradox-driven priorities (Table 3) would shift regulators from reactive detection to proactive prevention.

**Table 3:** Key Research Priorities for Advancing MNP Exposure Assessment and Regulatory Frameworks

Area	Key priorities
Standardized Biomonitoring & Exposure Thresholds	• Development of universal measurement units (e.g., particles per mL of blood, per gram of feces) for baseline exposure.
	• Refinement of analytical techniques to quantify MNP size, polymer composition, and chemical load.
	• Standardized protocols for sampling human biological fluids (blood, placenta, urine, lung tissue).
	• Inclusion of nanoplastics in exposure assessments.
	• Establishment of threshold exposure levels for regulatory action.
Biomarkers for MNP Exposure Assessment	• Integrate human biomonitoring with correlation of biomarker levels and MNP measurements.
	• Control for confounding factors like diet, chemical exposures, and lifestyle.
	• Use autopsy/biopsy studies to analyze MNP accumulation.
	• Develop ex vivo models to assess MNP-biomarker interactions.
	• Utilize epidemiological studies to profile biomarkers and identify population trends.
	• Pair diseased and healthy tissue samples.
	• Validation of biomarkers for human exposure.
Longitudinal Human Studies to Strengthen Risk Evidence	• Implement prospective cohort studies tracking individuals with varying MNP exposures.
	• Conduct case-control studies comparing MNP burden in individuals with/without specific diseases.
	• Develop multicenter human biomonitoring programs.
	• Measure MNP particle burden vs chemical metabolites to distinguish toxicity sources.
	• Strengthen epidemiological basis for regulatory decision-making.
	• Use multiomics approaches to establish MNP-specific biomarkers.
Clarifying Particle vs Chemical Risks for Regulation	• Differentiate between physical risks (particle accumulation) and chemical risks (adsorbed contaminants and additives).
	• Investigate whether toxicity is driven by particle presence or the release of harmful additives.
	• Study how MNP bioactivity varies by particle size, polymer type, and surface modifications.
	• Evaluate whether existing air pollution regulatory frameworks can apply to MNPs.
	• Determine which MNP sizes and polymer types pose greater risks for prioritization in regulations.
Toxicokinetics & Factors	• Dose–response mapping across tissues/polymers
	• Temporal exposure models accounting for:
	— Cumulative burden
	— Critical exposure windows
	— Generational effects
	— Advanced histopathology correlating MP deposition with
	— Subcellular damage
	— Immune activation
	— Organ dysfunction markers
	• No-observed-adverse-effect levels (NOAELs) for key polymers
	• Pathological thresholds for sensitive tissues
	• Susceptibility factors (age, disease status, genetics)

## 7 Implications

The plastiphere represents a novel Earth system compartment, where synthetic particles bypass traditional biogeochemical cycles to integrate directly into human biology—a hallmark of the Anthropocene. As plastic production continues to rise, the plastiphere will likely escalate from a biomarker to a determinant of population health. Addressing this risk requires Earth system models that incorporate human biological sinks—a new frontier for sustainability science. The plastiphere challenges us not only to confront an emerging biological reality, but to rethink the assumptions, tools, and epistemic structures through which we assess environmental risk. Its significance lies not just in the particles themselves, but in what their presence reveals: a field struggling to define thresholds, standardize evidence, and link exposure to consequence with scientific coherence. What emerges is a portrait of modern toxicology at a crossroads—caught between detection-driven reporting and the need for integrative, mechanistically grounded paradigms. The plastiphere offers such a paradigm: a bioparticulate system in which synthetic particles act not as inert residues but as semipersistent agents embedded within human physiology, capable of systemic interaction and biological integration. This duality defines the plastiphere—measurable, yet poorly contextualized; present, yet not fully understood. This review calls for a reframing of priorities. The path forward requires not just methodological innovation, but intellectual recalibration. We must move beyond fragmented data toward a system-level understanding that integrates exposure, fate, and effect. By defining the plastiphere as a bioparticulate system, we provide a scaffold to interrogate how synthetic materials co-opt biological pathways, bridging environmental science and physiology. This systems perspective provides a unified language for researchers, clinicians, and policymakers to quantify risks beyond mere presence.

Though originally developed in response to synthetic plastic particles, the plastiphere framework may also support future mapping of other persistent toxicants—such as particulate matter, pesticides, and metals—whose accumulation pathways and health effects incompletely unresolved. This extension would provide valuable insights for regulatory bodies and health practitioners, offering a comprehensive approach to environmental and public health challenges in the Anthropocene.

## Supplementary Material



## Notes

Text S1. Glossary; Text S2. Literature Search Methodology; Text S3. Scientific Literature Underpinning the Plastiphere Framework; Table S1. Microplastics in Human Tissues: Comprehensive Findings; Table S2. Summary of Microplastic Detection in Human Biological Samples Across Various Countries; Table S3. Particle Size Thresholds for Biological Barrier Penetration (PDF)

## §

Department of Biotechnology and Bioengineering, Centro de Investigación y de Estudios Avanzados del Instituto Politécnico Nacional, Av Instituto Politécnico Nacional 2508, San Pedro Zacatenco, Gustavo A. Madero, 07360, Ciudad de México, México
