# Supplementary material for: The Human Plastiphere: A Bioparticulate System Challenging Microplastic Risk Assessment and Governance
Source: Environ Sci Technol. 2025 Oct 1;59(45):24131–50. doi: 10.1021/acs.est.5c05922 (PMC12631990; doi:10.1021/acs.est.5c05922)
Supplement: Supplementary file 1 [file es5c05922_si_001.pdf]

Appendix A

Supplementary Material for

The Human Plastiphere: A Bio-Particulate System Challenging Microplastic Risk Assessment and Governance

V.C. Shruti[a,*], Gurusamy Kutralam-Muniasamy[b,*]

a. Department of Biotechnology and Bioengineering, Centro de Investigación y de Estudios Avanzados del Instituto Politécnico Nacional, Av Instituto Politécnico Nacional 2508, San Pedro Zacatenco, Gustavo A. Madero, 07360, Ciudad de México, México

b. CIITEC - IPN. Centro de Investigación e Innovación Tecnológica. Cda. de Cecati s/n, Santa Catarina, Azcapotzalco, 02250 Ciudad de México, CDMX

*Corresponding Authors

Text S1. Glossary

Text S2. Literature Search Methodology

Text S3. Scientific Literature Underpinning the Plastiphere Framework

Table S1. Microplastics in Human Tissues: Comprehensive Findings

Table S2. Summary of Microplastic Detection in Human Biological Samples Across Various Countries

Table S3. Particle Size Thresholds for Biological Barrier Penetration

Number of Texts: 3

Number of Tables: 3

Number of Pages: 36

Number of Supplementary Materials: 5

Submitted to ACS Environmental Science and Technology

Text S1. Glossary

1. Plastiphere
The systemic infiltration and accumulation of micro- and nanoplastics (MNPs) within living organism tissues, bodily fluids, and organs, along with their biological interactions.

2. Microplastics (MPs)
Synthetic polymer particles ranging from 1 μm to 5 mm in size, originating from plastic degradation or direct release (e.g., microbeads).

3. Nanoplastics (NPs)
Plastic particles <1 μm in size, capable of penetrating cells and crossing biological barriers (e.g., blood-brain, placental).

4. Organotropism
The preferential accumulation of micro- and nanoplastics (MNPs) in specific organs or tissues, as suggested by recurring detection patterns across anatomical sites (e.g., higher MP loads in the brain compared to the liver).

5. Oncotropism
The tendency of MNPs to localize within tumor microenvironments, potentially driven by the enhanced permeability and retention (EPR) effect characteristic of cancerous tissues.

6. Enhanced Permeability and Retention (EPR) Effect
A phenomenon where leaky blood vessels in tumors trap and retain particles (e.g., MPs, drug carriers) more efficiently than healthy tissues.

7. Biodistribution
The pattern of how MPs/NPs spread and deposit in different organs after entering the body (e.g., via ingestion, inhalation).

8. Fiber-Fragment Paradox
The discrepancy between environmental MP composition (dominated by fibers) and human tissue findings (dominated by fragments), raising questions about biological filtration or analytical biases.

9. Dynamic Equilibrium Model
A theory proposing that MPs in the body are in a steady state, with continuous input (exposure) balanced by excretion (renal, hepatic).

10. Progressive Sequestration Model
A competing theory suggesting MPs irreversibly accumulate in tissues (e.g., plaques, fat), leading to long-term biological impacts.

11. Transplacental Passage

The ability of MPs/NPs to cross the placental barrier, potentially exposing fetuses to synthetic particles.

12. Mucociliary Clearance
The respiratory system's mechanism to trap and remove inhaled particles (e.g., MPs) via mucus and cilia.

13. Pyrolysis-GC/MS
An analytical technique that heats samples to identify polymers by their thermal decomposition products.

14. Raman Microscopy
A spectroscopy method used to detect and characterize MPs based on their molecular vibrations.

15. Bioaccumulation
The gradual buildup of substances (e.g., MPs, toxins) in an organism over time, often exceeding environmental concentrations.

16. NOAEL (No-Observed-Adverse-Effect Level)
The highest exposure level at which no harmful effects are detected—currently undefined for most MPs.

17. Particulate Matter (PM) Co-Exposure
The simultaneous inhalation of MPs with other airborne pollutants (e.g., PM2.5), complicating health effect studies.

18. Tight Junction Proteins
Proteins that form barriers between cells (e.g., in intestines, blood-brain barrier); MP exposure may disrupt them.

19. Creeping Fat (in Crohn's Disease)
Inflamed fat tissue wrapping around intestines, found to contain 6× higher MPs than adjacent gut tissue.

20. Phantom Chemical Dilemma
The challenge of attributing detected additives (e.g., phthalates) to plastic particles versus other sources (food, cosmetics).

21. Bio-Particulate System: A network of non-biological particles that exhibits systemic integration into living organisms.

Text S2. Literature Search Methodology

A systematic literature search was conducted on February 26, 2025 across PubMed, Web of Science, Scopus, and EMBASE to identify human studies on micro- and nanoplastic (MNP) exposure. The search used the terms: ("microplastic*" OR "nanoplastic*") AND ("human*" OR "tissue*" OR "organ*" OR "blood" OR "placenta*" OR "urine" OR "fluid*" OR "tumor*" OR "cancerous"), limited to original research published between 2016-2025 (Until February). After initial retrieval, titles and abstracts were screened independently by two authors (GKM and VCS) to identify potentially relevant studies. Full texts of selected papers were then obtained and thoroughly evaluated by both researchers. We ultimately included 89 studies that reported MNP detection in human biological samples (blood, urine, placental fluid, tumor tissues, etc.) using validated analytical methods (Py-GC/MS, Raman microscopy, μFTIR). Exclusion criteria removed animal/cell studies, environmental-only analyses, review articles, and investigations with high contamination risk or unverified protocols. The final selection focused on direct evidence of MNP bioaccumulation across various tissue types, including comparative analyses of cancerous versus non-cancerous tissues, to comprehensively assess the human plastiphere phenomenon.

Text S3. Scientific Literature Underpinning the Plastiphere Framework

Colon tumoral and colon

1. Cetin, M.; Demirkaya Miloglu, F.; Kilic Baygutalp, N.; Ceylan, O.; Yildirim, S.; Eser, G.; Gul, H. İ. Higher Number of Microplastics in Tumoral Colon Tissues from Patients with Colorectal Adenocarcinoma. *Environ. Chem. Lett.* **2023**, *21*(2), 639–646.
2. Ibrahim, Y. S.; Tuan Anuar, S.; Azmi, A. A.; Wan Mohd Khalik, W. M. A.; Lehata, S.; Hamzah, S. R.; Ismail, D.; Ma, Z. F.; Dzulkarnaen, A.; Zakaria, Z.; Mustaffa, N. Detection of Microplastics in Human Colectomy Specimens. *JGH Open* **2021**, *5*(1), 116–121.

Stomach

3. Özsoy, S.; Gündogdu, S.; Sezigen, S.; Tasalp, E.; Ikiz, D. A.; Kideys, A. E. Presence of Microplastics in Human Stomachs. *Forensic Sci. Int.* **2024**, *364*, 112246.

Illeum and mesentric fat

4. Wu, F.; Wu, F.; Liu, X.; Xie, W.; Liang, Y.; Ye, Y.; Xiao, X.; Sun, K.; Bai, L.; Liu, S.; Liu, Z. Microplastics Accumulation in Fibrotic Intestinal Tissue of Crohn's Disease Patients. *Environ. Res.* **2025**, *232*, 121077.

Gastric tumor

5. Zhao, J.; Zhang, H.; Shi, L.; Jia, Y.; Sheng, H. Detection and Quantification of Microplastics in Various Types of Human Tumor Tissues. *Ecotoxicol. Environ. Saf.* **2024**, *283*, 116818.

Feces

6. Hartmann, C.; Lomako, I.; Schachner, C.; El Said, E.; Abert, J.; Satrapa, V.; Kaiser, A. M.; Walch, H.; Köppel, S. Assessment of Microplastics in Human Stool: A Pilot Study Investigating the Potential Impact of Diet-Associated Scenarios on Oral Microplastics Exposure. *Sci. Total Environ.* **2024**, *951*, 175825.
7. Schwenger, K. J.; Ghorbani, Y.; Bharatselvam, S.; Chen, L.; Chomiak, K. M.; Tyler, A. C.; Eddingsaas, N. C.; Fischer, S. E.; Jackson, T. D.; Okrainec, A.; Allard, J. P. Links between Fecal Microplastics and Parameters Related to Metabolic Dysfunction-Associated Steatotic Liver Disease (MASLD) in Humans: An Exploratory Study. *Sci. Total Environ.* **2024**, *953*, 176153.
8. Yan, Z.; Liu, Y.; Zhang, T.; Zhang, F.; Ren, H.; Zhang, Y. Analysis of Microplastics in Human Feces Reveals a Correlation between Fecal Microplastics and Inflammatory Bowel Disease Status. *Environ. Sci. Technol.* **2021**, *56* (1), 414–421.
9. Zhu, L.; Wu, Z.; Dong, J.; Zhao, S.; Zhu, J.; Wang, W.; Ma, F.; An, L. Unveiling Small-Sized Plastic Particles Hidden Behind Large-Sized Ones in Human Excretion and Their Potential Sources. *Environ. Sci. Technol.* **2024**, *58*(27), 11901–11911.
10. Song, Y.; Zhang, J.; Shen, X.; Yang, L.; Jia, Y.; Song, F.; Huang, Y.; Han, B.; Zhou, S.; Zhang, N.; Ma, G. Microplastics in Stools and Their Influencing Factors Among Young Adults from Three Cities in China: A Multicenter Cross-Sectional Study. *Environ. Pollut.* **2025**, *364*, 125168.
11. Ke, D.; Zheng, J.; Liu, X.; Xu, X.; Zhao, L.; Gu, Y.; Yang, R.; Liu, S.; Yang, S.; Du, J.; Chen, B. Occurrence of Microplastics and Disturbance of Gut Microbiota: A Pilot Study of Preschool Children in Xiamen, China. *EBioMedicine* **2023**, *97*, –.

12. Gao, B.; Chen, L.; Wu, L.; Zhang, S.; Zhao, S.; Mo, Z.; Chen, Z.; Tu, P. Association Between Microplastics and the Functionalities of Human Gut Microbiome. *Ecotoxicol. Environ. Saf.* **2025**, *290*, 117497.
13. Zhang, N.; Li, Y. B.; He, H. R.; Zhang, J. F.; Ma, G. S. You Are What You Eat: Microplastics in the Feces of Young Men Living in Beijing. *Sci. Total Environ.* **2021**, *767*, 144345.
14. Liu, S.; Guo, J.; Liu, X.; Yang, R.; Wang, H.; Sun, Y.; Chen, B.; Dong, R. Detection of Various Microplastics in Placentas, Meconium, Infant Feces, Breastmilk and Infant Formula: A Pilot Prospective Study. *Sci. Total Environ.* **2023**, *854*, 158699.
15. Braun, T.; Ehrlich, L.; Henrich, W.; Koeppel, S.; Lomako, I.; Schwabl, P.; Liebmann, B. Detection of Microplastic in Human Placenta and Meconium in a Clinical Setting. *Pharmaceutics* **2021**, *13*(7), 921.
16. Hasanah, U.; Amqam, H.; Septami, A. E.; Chalid, M.; Aris, A. Z. Plasticizing Pregnancy: Microplastics Identified in Expectant Mothers' Feces. *Environ. Health Insights* **2024**, *18*, 11786302241235810.
17. Luqman, A.; Nugrahapraja, H.; Wahyuono, R. A.; Islami, I.; Haekal, M. H.; Fardiansyah, Y.; Putri, B. Q.; Amalludin, F. I.; Rofiqa, E. A.; Götz, F.; Wibowo, A. T. Microplastic Contamination in Human Stools, Foods, and Drinking Water Associated with Indonesian Coastal Population. *Environments* **2021**, *8*(12), 138.
18. Wibowo, A. T.; Nugrahapraja, H.; Wahyuono, R. A.; Islami, I.; Haekal, M. H.; Fardiansyah, Y.; Sugiyo, P. W. W.; Putro, Y. K.; Fauzia, F. N.; Santoso, H.; Götz, F. Microplastic Contamination in the Human Gastrointestinal Tract and Daily Consumables Associated with an Indonesian Farming Community. *Sustainability* **2021**, *13*(22), 12840.
19. Schwabl, P.; Köppel, S.; Königshofer, P.; Bucsics, T.; Trauner, M.; Reiberger, T.; Liebmann, B. Detection of Various Microplastics in Human Stool: A Prospective Case Series. *Ann. Intern. Med.* **2019**, *171*(7), 453–457.

Lung tumor

20. Zhao, J.; Zhang, H.; Shi, L.; Jia, Y.; Sheng, H. Detection and Quantification of Microplastics in Various Types of Human Tumor Tissues. *Ecotoxicol. Environ. Saf.* **2024**, *283*, 116818.

Lung healthy

21. Amato-Lourenço, L. F.; Carvalho-Oliveira, R.; Júnior, G. R.; dos Santos Galvão, L.; Ando, R. A.; Mauad, T. Presence of Airborne Microplastics in Human Lung Tissue. *J. Hazard. Mater.* **2021**, *416*, 126124.
22. Chen, Q.; Gao, J.; Yu, H.; Su, H.; Yang, Y.; Cao, Y.; Zhang, Q.; Ren, Y.; Hollert, H.; Shi, H.; et al. An Emerging Role of Microplastics in the Etiology of Lung Ground Glass Nodules: A Preliminary Study. Environ. Sci. Technol. Lett. 2023, *10* (3), 280-286.
23. Jenner, L. C.; Rotchell, J. M.; Bennett, R. T.; Cowen, M.; Tentzeris, V.; Sadofsky, L. R. Detection of Microplastics in Human Lung Tissue Using μFTIR Spectroscopy. *Sci. Total Environ.* **2022**, *831*, 154907.
24. Wang, S.; Lu, W.; Cao, Q.; Tu, C.; Zhong, C.; Qiu, L.; Li, S.; Zhang, H.; Lan, M.; Qiu, L.; Li, X. Microplastics in the Lung Tissues Associated with Blood Test Index. *Toxics* **2023**, *11*(9), 759.

Sputum

25. Huang, S.; Huang, X.; Bi, R.; Guo, Q.; Yu, X.; Zeng, Q.; Huang, Z.; Liu, T.; Wu, H.; Chen, Y.; Xu, J. Detection and Analysis of Microplastics in Human Sputum. *Environ. Sci. Technol.* **2022**, *56* (4), 2476–2486.
26. Jiang, Y.; Han, J.; Na, J.; Fang, J.; Qi, C.; Lu, J.; Liu, X.; Zhou, C.; Feng, J.; Zhu, W.; Liu, L. Exposure to Microplastics in the Upper Respiratory Tract of Indoor and Outdoor Workers. *Chemosphere* **2022**, *307*, 136067.
27. Jahedi, F.; Fard, N. J. H.; Moghadam, M. A.; Takdastan, A.; Shoushtari, M. H.; Dehbandi, R.; Turner, A. Microplastics in Urine, Sputum and Lung Lavage Fluid from Patients with Respiratory Illnesses. *Environ. Res.* **2025**, 121278.
28. Momeni, M. K.; Taghipour, H.; Ghayebzadeh, M.; Mohammadi, M.; Keikhaee, R. Isolation and Characterization of Microplastics from the Human Respiratory System: Sputum, Broncho-Alveolar Lavage Fluid, and Pleural Fluid Simultaneously. *Environ. Pollut.* **2025**, *365*, 125389.

Balf

29. Jahedi, F.; Fard, N. J. H.; Moghadam, M. A.; Takdastan, A.; Shoushtari, M. H.; Dehbandi, R.; Turner, A. Microplastics in Urine, Sputum and Lung Lavage Fluid from Patients with Respiratory Illnesses. *Environ. Res.* **2025**, 121278.
30. Momeni, M. K.; Taghipour, H.; Ghayebzadeh, M.; Mohammadi, M.; Keikhaee, R. Isolation and Characterization of Microplastics from the Human Respiratory System: Sputum, Broncho-Alveolar Lavage Fluid, and Pleural Fluid Simultaneously. *Environ. Pollut.* **2025**, *365*, 125389.
31. Chen, C.; Liu, F.; Quan, S.; º x L.; Shen, A.; Jiao, A.; Qi, H.; Yu, G. Microplastics in the Bronchoalveolar Lavage Fluid of Chinese Children: Associations with Age, City Development, and Disease Features. *Environ. Sci. Technol.* **2023**, *57* (34), 12594–12601.
32. Lu, W.; Li, X.; Wang, S.; Tu, C.; Qiu, L.; Zhang, H.; Zhong, C.; Li, S.; Liu, Y.; Liu, J.; Zhou, Y. New Evidence of Microplastics in the Lower Respiratory Tract: Inhalation through Smoking. *Environ. Sci. Technol.* **2023**, *57* (23), 8496–8505.
33. Qiu, L.; Lu, W.; Tu, C.; Li, X.; Zhang, H.; Wang, S.; Chen, M.; Zheng, X.; Wang, Z.; Lin, M.; Zhang, Y. Evidence of Microplastics in Bronchoalveolar Lavage Fluid among Never-Smokers: A Prospective Case Series. *Environ. Sci. Technol.* **2023**, *57* (6), 2435–2444.
34. Uogintė, I.; Vailionytė, A.; Skapas, M.; Bolanos, D.; Bagurskienė, E.; Gruslys, V.; Aldonytė, R.; Byčenkienė, S. New Evidence of the Presence of Micro- and Nanoplastic Particles in Bronchioalveolar Lavage Samples of Clinical Trial Subjects. *Heliyon* **2023**, *9*(9), –.
35. Baeza-Martínez, C.; Olmos, S.; González-Pleiter, M.; López-Castellanos, J.; García-Pachón, E.; Masiá-Canuto, M.; Hernández-Blasco, L.; Bayo, J. First Evidence of Microplastics Isolated in European Citizens' Lower Airway. *J. Hazard. Mater.* **2022**, *438*, 129439.
36. Alpaydin, A. Ö.; Uçan, E. S.; Köktürk, M.; Atamanalp, M.; Kalyoncu, Ç.; Yiğit, S.; Uçar, A.; Şimşek, G. Ö.; Tertemiz, K. C.; Karaçam, V.; Ulukuş, E. Ç. Microplastics, as a Risk Factor in the Development of Interstitial Lung Disease—A Preliminary Study. *Environ. Pollut.* **2024**, *363*, 125054.

Nasal cavity

37. Jiang, Y.; Han, J.; Na, J.; Fang, J.; Qi, C.; Lu, J.; Liu, X.; Zhou, C.; Feng, J.; Zhu, W.; Liu, L. Exposure to Microplastics in the Upper Respiratory Tract of Indoor and Outdoor Workers. *Chemosphere* **2022**, *307*, 136067.
38. Zhang, M.; Liu, T.; Zhang, L.; Hua, Z.; Guo, Z.; Dong, J.; Tan, Q.; Xie, Y.; Yin, X.; Yan, L.; Pan, G. Assessment of Microplastic Exposure in Nasal Lavage Fluid and the Influence of Face Masks. *J. Hazard. Mater.* **2024**, *480*, 136069.
39. Min, H. J.; Kim, K. S.; Kim, H.; Gong, J.; Jeong, J. Identification and Characterization of Microplastics in Human Nasal Samples. *Int. Forum Allergy Rhinol.* **2024**, *14*(12), 1943.

Pleural fluid

40. Momeni, M. K.; Taghipour, H.; Ghayebzadeh, M.; Mohammadi, M.; Keikhaee, R. Isolation and Characterization of Microplastics from the Human Respiratory System: Sputum, Broncho-Alveolar Lavage Fluid, and Pleural Fluid Simultaneously. *Environ. Pollut.* **2025**, *365*, 125389.

Nasopharyngeal fluid

41. Min, H. J.; Kim, K. S.; Kim, H.; Gong, J.; Jeong, J. Identification and Characterization of Microplastics in Human Nasal Samples. *Int. Forum Allergy Rhinol.* **2024**, *14*(12), 1943.

Cervical cancerous and paracancerous tissue

42. Xu, H.; Dong, C.; Yu, Z.; Ozaki, Y.; Hu, Z.; Zhang, B.; Yao, W.; Yu, J.; Xie, Y. Detection and Analysis of Microplastics in Tissues and Blood of Human Cervical Cancer Patients. *Environ. Res.* **2024**, *259*, 119498.
43. Zhao, J.; Zhang, H.; Shi, L.; Jia, Y.; Sheng, H. Detection and Quantification of Microplastics in Various Types of Human Tumor Tissues. *Ecotoxicol. Environ. Saf.* **2024**, *283*, 116818.

Prostate tumors

44. Demirelli, E., Tepe, Y., Oğuz, U., Aydın, H., Kodat, M., Tok, D.S., Sönmez, M.G. and Öğreden, E., 2024. The first reported values of microplastics in prostate. *BMC urology*, *24*(1), p.106.
45. Deng, C.; Zhu, J.; Fang, Z.; Yang, Y.; Zhao, Q.; Zhang, Z.; Jin, Z.; Jiang, H. Identification and Analysis of Microplastics in Para-Tumor and Tumor of Human Prostate. *EBioMedicine* **2024**, *108*, 104429.

Penis

46. Codrington, J.; Varnum, A. A.; Hildebrandt, L.; Pröfrock, D.; Bidhan, J.; Khodamoradi, K.; Höhme, A. L.; Held, M.; Evans, A.; Velasquez, D.; Yarborough, C. C. Detection of Microplastics in the Human Penis. *Int. J. Impot. Res.* **2024**.

Penile tumor

47. Wang, M., Liu, Q., Zhang, X., Jiang, H. and Zhang, X., 2025. Identification and analysis of microplastics in human penile cancer tissues. *Science of The Total Environment*, *969*, p.178815.

Pancreatic tumor

48. Zhao, J.; Zhang, H.; Shi, L.; Jia, Y.; Sheng, H. Detection and Quantification of Microplastics in Various Types of Human Tumor Tissues. *Ecotoxicol. Environ. Saf.* **2024**, *283*, 116818.

Uterine fibroids

49. Xu, H.; Dong, C.; Yu, Z.; Hu, Z.; Yu, J.; Ma, D.; Yao, W.; Qi, X.; Ozaki, Y.; Xie, Y. First Identification of Microplastics in Human Uterine Fibroids and Myometrium. *Environ. Pollut.* **2024**, *360*, 124632.

Semen

50. Zhang, C.; Zhang, G.; Sun, K.; Ren, J.; Zhou, J.; Liu, X.; Lin, F.; Yang, H.; Cao, J.; Nie, L.; Zhang, P. Association of Mixed Exposure to Microplastics with Sperm Dysfunction: A Multi-Site Study in China. *EBioMedicine* **2024**, *108*, 104481.
51. Montano, L.; Giorgini, E.; Notarstefano, V.; Notari, T.; Ricciardi, M.; Piscopo, M.; Motta, O. Raman Microspectroscopy Evidence of Microplastics in Human Semen. *Sci. Total Environ.* **2023**, *901*, 165922.
52. Li, N.; Yang, H.; Dong, Y.; Wei, B.; Liang, L.; Yun, X.; Tian, J.; Zheng, Y.; Duan, S.; Zhang, L. Prevalence and Implications of Microplastic Contaminants in General Human Seminal Fluid: A Raman Spectroscopic Study. *Sci. Total Environ.* **2024**, *937*, 173522.
53. Chen, Y.; Cheng, C.; Xu, W.; Cui, Y.; Tian, Y.; Jiang, Y.; Yuan, Y.; Qian, R.; Wang, Y.; Zheng, L.; Chen, H. Occurrence, Toxicity and Removal of Polystyrene Microplastics and Nanoplastics in Human Sperm. *Environ. Chem. Lett.* **2024**, *22* (5), 2159–2165.
54. Zhao, Q.; Zhu, L.; Weng, J.; Jin, Z.; Cao, Y.; Jiang, H.; Zhang, Z. Detection and Characterization of Microplastics in the Human Testis and Semen. *Sci. Total Environ.* **2023**, *877*, 162713.

Testis

55. Zhao, Q.; Zhu, L.; Weng, J.; Jin, Z.; Cao, Y.; Jiang, H.; Zhang, Z. Detection and Characterization of Microplastics in the Human Testis and Semen. *Sci. Total Environ.* **2023**, *877*, 162713.
56. Hu, C. J.; Garcia, M. A.; Nihart, A.; Liu, R.; Yin, L.; Adolphi, N.; Gallego, D. F.; Kang, H.; Campen, M. J.; Yu, X. Microplastic Presence in Dog and Human Testis and Its Potential Association with Sperm Count and Weights of Testis and Epididymis. *Toxicol. Sci.* **2024**, *200* (2), 235–240.

Placenta

57. Zurub, R. E.; Bainbridge, S.; Rahman, L.; Halappanavar, S.; El-Chaâr, D.; Wade, M. G. Particulate Contamination of Human Placenta: Plastic and Non-Plastic. Environ. Adv. 2024, 17, 100555.
58. Ragusa, A.; Svelato, A.; Santacroce, C.; Catalano, P.; Notarstefano, V.; Carnevali, O.; Papa, F.; Rongioletti, M. C. A.; Baiocco, F.; Draghi, S.; D'Amore, E. Plasticenta: First Evidence of Microplastics in Human Placenta. *Environ. Int.* **2021**, *146*, 106274.
59. Liu, S.; Guo, J.; Liu, X.; Yang, R.; Wang, H.; Sun, Y.; Chen, B.; Dong, R. Detection of Various Microplastics in Placentas, Meconium, Infant Feces, Breastmilk and Infant Formula: A Pilot Prospective Study. *Sci. Total Environ.* **2023**, *854*, 158699.
60. Amereh, F.; Amjadi, N.; Mohseni-Bandpei, A.; Isazadeh, S.; Mehrabi, Y.; Eslami, A.; Naeiji, Z.; Rafiee, M. Placental Plastics in Young Women from General Population Correlate with Reduced Foetal Growth in IUGR Pregnancies. *Environ. Pollut.* **2022**, *314*, 120174.
61. Garcia, M. A.; Liu, R.; Nihart, A.; El Hayek, E.; Castillo, E.; Barrozo, E. R.; Suter, M. A.; Bleske, B.; Scott, J.; Forsythe, K.; Gonzalez-Estrella, J. Quantitation and Identification of Microplastics Accumulation in Human Placental Specimens Using Pyrolysis Gas Chromatography Mass Spectrometry. *Toxicol. Sci.* **2024**, *199*(1), 81–88.
62. Braun, T.; Ehrlich, L.; Henrich, W.; Koeppel, S.; Lomako, I.; Schwabl, P.; Liebmann, B. Detection of Microplastic in Human Placenta and Meconium in a Clinical Setting. *Pharmaceutics* **2021**, *13*(7), 921.

63. Halfar, J.; Čabanová, K.; Vávra, K.; Delongová, P.; Motyka, O.; Špaček, R.; Kukutschová, J.; Šimetka, O.; Heviánková, S. Microplastics and Additives in Patients with Preterm Birth: The First Evidence of Their Presence in Both Human Amniotic Fluid and Placenta. *Chemosphere* **2023**, *343*, 140301.
64. Liu, S., Liu, X., Guo, J., Yang, R., Wang, H., Sun, Y., Chen, B. and Dong, R., 2022. The association between microplastics and microbiota in placentas and meconium: the first evidence in humans. *Environmental science & technology*, *57*(46), pp.17774-17785.

Myometrium

65. Xu, H.; Dong, C.; Yu, Z.; Hu, Z.; Yu, J.; Ma, D.; Yao, W.; Qi, X.; Ozaki, Y.; Xie, Y. First Identification of Microplastics in Human Uterine Fibroids and Myometrium. *Environ. Pollut.* **2024**, *360*, 124632.

Amniotic fluid

66. Halfar, J.; Čabanová, K.; Vávra, K.; Delongová, P.; Motyka, O.; Špaček, R.; Kukutschová, J.; Šimetka, O.; Heviánková, S. Microplastics and Additives in Patients with Preterm Birth: The First Evidence of Their Presence in Both Human Amniotic Fluid and Placenta. *Chemosphere* **2023**, *343*, 140301.
67. Sun, H.; Su, X.; Mao, J.; Liu, Y.; Li, G.; Du, Q. Microplastics in Maternal Blood, Fetal Appendages, and Umbilical Vein Blood. *Ecotoxicol. Environ. Saf.* **2024**, *287*, 117300.
68. Xue, J.; Xu, Z.; Hu, X.; Lu, Y.; Zhao, Y.; Zhang, H. Microplastics in Maternal Amniotic Fluid and Their Associations with Gestational Age. *Sci. Total Environ.* **2024**, *920*, 171044.

Ovarian follicular fluid

69. Montano, L.; Raimondo, S.; Piscopo, M.; Ricciardi, M.; Guglielmino, A.; Chamayou, S.; Gentile, R.; Gentile, M.; Rapisarda, P.; Conti, G. O.; Ferrante, M. First Evidence of Microplastics in Human Ovarian Follicular Fluid: An Emerging Threat to Female Fertility. *Ecotoxicol. Environ. Saf.* **2025**, *291*, 117868.
70. Weina, L.; Yena, H.; Liang, H.; Huan, Z.; Gang, L.; Ge, L. P-762 Discovery and Quantification of Microplastics in Human Cumulus Granulosa Cells. *Hum. Reprod.* **2024**, *39*(Suppl_1), deae108–1081.

Meconium

71. Liu, S.; Guo, J.; Liu, X.; Yang, R.; Wang, H.; Sun, Y.; Chen, B.; Dong, R. Detection of Various Microplastics in Placentas, Meconium, Infant Feces, Breastmilk and Infant Formula: A Pilot Prospective Study. *Sci. Total Environ.* **2023**, *854*, 158699.
72. Braun, T.; Ehrlich, L.; Henrich, W.; Koeppel, S.; Lomako, I.; Schwabl, P.; Liebmann, B. Detection of Microplastic in Human Placenta and Meconium in a Clinical Setting. *Pharmaceutics* **2021**, *13*(7), 921.
73. Li, Z.; Wang, J.; Gao, X.; Du, J.; Sui, H.; Wu, J.; Zhong, Y.; Liang, B.; Huang, Y.; Ye, R.; Deng, Y. Investigation of Microplastics (≥10 μm) in Meconium by Fourier Transform Infrared Microspectroscopy. *Toxics* **2023**, *11*(4), 310.

Cervicovaginal lavage fluids

74. Shim, Y. and Min, H., 2025. Identification and Characterization of Microplastics in Human Cervicovaginal Lavage Fluids Using Raman Spectroscopy: A Preliminary Study. *Life*, *15*(3), p.357.

Fetal appendages

75. Sun, H.; Su, X.; Mao, J.; Liu, Y.; Li, G.; Du, Q. Microplastics in Maternal Blood, Fetal Appendages, and Umbilical Vein Blood. *Ecotoxicol. Environ. Saf.* **2024**, *287*, 117300.

Kidney

76. Exacoustos, O.; Artini, C.; Massardo, S.; Caboni, C.; Pastorino, A.; Chiarenza, S.; Zaza, G.; Stallone, G.; Ghiggeri, G. M.; Angeletti, A.; Lugani, F. #6111 First Identification and Characterization of Microplastics in Human Kidney and Urine. *Nephrol. Dial. Transplant.* **2023**, *38*(Suppl. 1), gfad063a_6111.
77. Horvatits, T.; Tamminga, M.; Liu, B.; Sebode, M.; Carambia, A.; Fischer, L.; Püschel, K.; Huber, S.; Fischer, E. K. Microplastics Detected in Cirrhotic Liver Tissue. *EBioMedicine* **2022**, *82*, 104119.
78. Nihart, A. J.; Garcia, M. A.; El Hayek, E.; Liu, R.; Olewine, M.; Kingston, J. D.; Castillo, E. F.; Gullapalli, R. R.; Howard, T.; Bleske, B.; Scott, J. Bioaccumulation of Microplastics in Decedent Human Brains. *Nat. Med.* **2025**, https://doi.org/10.1038/s41591-025-XXXXX.
79. Massardo, S.; Verzola, D.; Alberti, S.; Caboni, C.; Santostefano, M.; Verrina, E. E.; Angeletti, A.; Lugani, F.; Ghiggeri, G. M.; Bruschi, M.; Candiano, G. MicroRaman Spectroscopy Detects the Presence of Microplastics in Human Urine and Kidney Tissue. *Environ. Int.* **2024**, *184*, 108444.

Urine

80. Massardo, S.; Verzola, D.; Alberti, S.; Caboni, C.; Santostefano, M.; Verrina, E. E.; Angeletti, A.; Lugani, F.; Ghiggeri, G. M.; Bruschi, M.; Candiano, G. MicroRaman Spectroscopy Detects the Presence of Microplastics in Human Urine and Kidney Tissue. *Environ. Int.* **2024**, *184*, 108444.
81. Exacoustos, O.; Artini, C.; Massardo, S.; Caboni, C.; Pastorino, A.; Chiarenza, S.; Zaza, G.; Stallone, G.; Ghiggeri, G. M.; Angeletti, A.; Lugani, F. #6111 First Identification and Characterization of Microplastics in Human Kidney and Urine. *Nephrol. Dial. Transplant.* **2023**, *38*(Suppl. 1), gfad063a_6111.
82. Pironti, C.; Notarstefano, V.; Ricciardi, M.; Motta, O.; Giorgini, E.; Montano, L. First Evidence of Microplastics in Human Urine, a Preliminary Study of Intake in the Human Body. *Toxics* **2022**, *11* (1), 40.
83. Song, X.; Chen, T.; Chen, Z.; Du, L.; Qiu, X.; Zhang, Y.; Li, Y.; Zhu, Y.; Tan, Z.; Mo, Y.; Feng, X. Micro (Nano) Plastics in Human Urine: A Surprising Contrast Between Chongqing's Urban and Rural Regions. *Sci. Total Environ.* **2024**, *917*, 170455.
84. Jahedi, F.; Fard, N. J. H.; Moghadam, M. A.; Takdastan, A.; Shoushtari, M. H.; Dehbandi, R.; Turner, A. Microplastics in Urine, Sputum and Lung Lavage Fluid from Patients with Respiratory Illnesses. *Environ. Res.* **2025**, 121278.
85. Zhu, L.; Wu, Z.; Dong, J.; Zhao, S.; Zhu, J.; Wang, W.; Ma, F.; An, L. Unveiling Small-Sized Plastic Particles Hidden Behind Large-Sized Ones in Human Excretion and Their Potential Sources. *Environ. Sci. Technol.* **2024**, *58*(27), 11901–11911.
86. Zhang, C.; Zhang, G.; Sun, K.; Ren, J.; Zhou, J.; Liu, X.; Lin, F.; Yang, H.; Cao, J.; Nie, L.; Zhang, P. Association of Mixed Exposure to Microplastics with Sperm Dysfunction: A Multi-Site Study in China. *EBioMedicine* **2024**, *108*, 104481.
87. Rotchell, J. M.; Austin, C.; Chapman, E.; Atherall, C. A.; Liddle, C. R.; Dunstan, T. S.; Blackburn, B.; Mead, A.; Filart, K.; Beeby, E.; Cunningham, K. Microplastics in Human Urine: Characterisation Using μFTIR and Sampling Challenges Using Healthy Donors and Endometriosis Participants. *Ecotoxicol. Environ. Saf.* **2024**, *274*, 116208.

Blood

88. Gao, B.; Chen, L.; Wu, L.; Zhang, S.; Zhao, S.; Mo, Z.; Chen, Z.; Tu, P. Association Between Microplastics and the Functionalities of Human Gut Microbiome. *Ecotoxicol. Environ. Saf.* **2025**, *290*, 117497.
89. Xu, H.; Dong, C.; Yu, Z.; Ozaki, Y.; Hu, Z.; Zhang, B.; Yao, W.; Yu, J.; Xie, Y. Detection and Analysis of Microplastics in Tissues and Blood of Human Cervical Cancer Patients. *Environ. Res.* **2024**, *259*, 119498.
90. Brits, M., Van Velzen, M.J., Sefiloglu, F.Ö., Scibetta, L., Groenewoud, Q., Garcia-Vallejo, J.J., Vethaak, A.D., Brandsma, S.H. and Lamoree, M.H., 2024. Quantitation of micro and nanoplastics in human blood by pyrolysis-gas chromatography–mass spectrometry. *Microplastics and Nanoplastics*, *4*(1), p.12.
91. Leslie, H. A.; Van Velzen, M. J.; Brandsma, S. H.; Vethaak, A. D.; Garcia-Vallejo, J. J.; Lamoree, M. H. Discovery and Quantification of Plastic Particle Pollution in Human Blood. *Environ. Int.* **2022**, *163*, 107199.
92. Leonard, S. V.; Liddle, C. R.; Atherall, C. A.; Chapman, E.; Watkins, M.; Calaminus, S. D.; Rotchell, J. M. Microplastics in Human Blood: Polymer Types, Concentrations and Characterisation Using μFTIR. *Environ. Int.* **2024**, *188*, 108751.
93. Lee, D. W.; Jung, J.; Park, S. A.; Lee, Y.; Kim, J.; Han, C.; Kim, H. C.; Lee, J. H.; Hong, Y. C. Microplastic Particles in Human Blood and Their Association with Coagulation Markers. *Sci. Rep.* **2024**, *14* (1), 1–10.
94. Alpaydin, A. Ö.; Uçan, E. S.; Köktürk, M.; Atamanalp, M.; Kalyoncu, Ç.; Yiğit, S.; Uçar, A.; Şimşek, G. Ö.; Tertemiz, K. C.; Karaçam, V.; Ulukuş, E. Ç. Microplastics, as a Risk Factor in the Development of Interstitial Lung Disease—A Preliminary Study. *Environ. Pollut.* **2024**, *363*, 125054.

Maternal Blood

95. Sun, H.; Su, X.; Mao, J.; Liu, Y.; Li, G.; Du, Q. Microplastics in Maternal Blood, Fetal Appendages, and Umbilical Vein Blood. *Ecotoxicol. Environ. Saf.* **2024**, *287*, 117300.

Umbilical Vein Blood

96. Sun, H.; Su, X.; Mao, J.; Liu, Y.; Li, G.; Du, Q. Microplastics in Maternal Blood, Fetal Appendages, and Umbilical Vein Blood. *Ecotoxicol. Environ. Saf.* **2024**, *287*, 117300. Arterial Plaques

Arterial Plaques

97. Marfella, R.; Prattichizzo, F.; Sardu, C.; Fulgenzi, G.; Graciotti, L.; Spadoni, T.; D'Onofrio, N.; Scisciola, L.; La Grotta, R.; Frigé, C.; Pellegrini, V. Microplastics and Nanoplastics in Atheromas and Cardiovascular Events. *N. Engl. J. Med.* **2024**, *390* (10), 900–910.
98. Liu, S.; Wang, C.; Yang, Y.; Du, Z.; Li, L.; Zhang, M.; Ni, S.; Yue, Z.; Yang, K.; Wang, Y.; Li, X. Microplastics in Three Types of Human Arteries Detected by Pyrolysis-Gas Chromatography/Mass Spectrometry (Py-GC/MS). *J. Hazard. Mater.* **2024**, *469*, 133855.

Thrombus

99. Wu, D.; Feng, Y.; Wang, R.; Jiang, J.; Guan, Q.; Yang, X.; Wei, H.; Xia, Y.; Luo, Y. Pigment Microparticles and Microplastics Found in Human Thrombi Based on Raman Spectral Evidence. *J. Adv. Res.* **2023**, *49*, 141–150.
100. Wang, T.; Yi, Z.; Liu, X.; Cai, Y.; Huang, X.; Fang, J.; Shen, R.; Lu, W.; Xiao, Y.; Zhuang, W.; Guo, S. Multimodal Detection and Analysis of Microplastics in Human Thrombi from Multiple Anatomically Distinct Sites. *EBioMedicine* **2024**, *103*, 104510.

Aorta/coronary artery/cartoid artery

101. Liu, S.; Wang, C.; Yang, Y.; Du, Z.; Li, L.; Zhang, M.; Ni, S.; Yue, Z.; Yang, K.; Wang, Y.; Li, X. Microplastics in Three Types of Human Arteries Detected by Pyrolysis-Gas Chromatography/Mass Spectrometry (Py-GC/MS). *J. Hazard. Mater.* **2024**, *469*, 133855.

Brain

102. Nihart, A. J.; Garcia, M. A.; El Hayek, E.; Liu, R.; Olewine, M.; Kingston, J. D.; Castillo, E. F.; Gullapalli, R. R.; Howard, T.; Bleske, B.; Scott, J. Bioaccumulation of Microplastics in Decedent Human Brains. *Nat. Med.* **2025**, https://doi.org/10.1038/s41591-025-XXXXX.

103. Amato-Lourenço, L. F.; Dantas, K. C.; Júnior, G. R.; Paes, V. R.; Ando, R. A.; de Oliveira Freitas, R.; da Costa, O. M. M. M.; Rabelo, R. S.; Bispo, K. C. S.; Carvalho-Oliveira, R.; Mauad, T. Microplastics in the Olfactory Bulb of the Human Brain. *JAMA Netw. Open* **2024**, *7* (9), e2440018.

Cerebrospinal fluid

104. Xie, J.; Sun, Y.; Ma, Y.; Wu, D.; Zhang, Z. Blood–Brain Barrier Damage Accelerates the Accumulation of Micro- and Nanoplastics in the Human Central Nervous System. *J. Hazard. Mater.* **2024**, *480*, 136028.

Liver

105. Horvatits, T.; Tamminga, M.; Liu, B.; Sebode, M.; Carambia, A.; Fischer, L.; Püschel, K.; Huber, S.; Fischer, E. K. Microplastics Detected in Cirrhotic Liver Tissue. *EBioMedicine* **2022**, *82*, 104119.

106. Nihart, A. J.; Garcia, M. A.; El Hayek, E.; Liu, R.; Olewine, M.; Kingston, J. D.; Castillo, E. F.; Gullapalli, R. R.; Howard, T.; Bleske, B.; Scott, J. Bioaccumulation of Microplastics in Decedent Human Brains. *Nat. Med.* **2025**, https://doi.org/10.1038/s41591-025-XXXXX.

Spleen

107. Horvatits, T.; Tamminga, M.; Liu, B.; Sebode, M.; Carambia, A.; Fischer, L.; Püschel, K.; Huber, S.; Fischer, E. K. Microplastics Detected in Cirrhotic Liver Tissue. *EBioMedicine* **2022**, *82*, 104119.

Gallstone

108. Zhang, D.; Wu, C.; Liu, Y.; Li, W.; Li, S.; Peng, L.; Kang, L.; Ullah, S.; Gong, Z.; Li, Z.; Ding, D. Microplastics Are Detected in Human Gallstones and Have the Ability to Form Large Cholesterol-Microplastic Heteroaggregates. *J. Hazard. Mater.* **2024**, *467*, 133631.

Bone, Cartilage, Intervertebral disc

109. Yang, Q.; Peng, Y.; Wu, X.; Cao, X.; Zhang, P.; Liang, Z.; Zhang, J.; Zhang, Y.; Gao, P.; Fu, Y.; Liu, P. Microplastics in Human Skeletal Tissues: Presence, Distribution, and Health Implications. *Environ. Int.* **2025**, *183*, 109316.

Bone marrow

110. Guo, X., Wang, L., Wang, X., Li, D., Wang, H., Xu, H., Liu, Y., Kang, R., Chen, Q., Zheng, L. and Wu, S., 2024. Discovery and analysis of microplastics in human bone marrow. *Journal of Hazardous Materials*, *477*, p.135266.

Synovial fluids

111. Niessink, T.; Schoenmakers, J. W.; Janssen, M.; Wouthuyzen-Bakker, M.; Piersma, S.; van Oosten, M.; van Dijl, J. M.; Otto, C.; Jansen, T. L. Raman Spectroscopy

Reveals Microparticles in Synovial Fluids of Patients with Suspected Implant-Related Complications. *J. Raman Spectrosc.* **2025**, *56*(2), 127–134.

Synovial tissue

112. Li, Z.; Zheng, Y.; Maimaiti, Z.; Fu, J.; Yang, F.; Li, Z. Y.; Shi, Y.; Hao, L. B.; Chen, J. Y.; Xu, C. Identification and Analysis of Microplastics in Human Lower Limb Joints. *J. Hazard. Mater.* **2024**, *461*, 132640.

Saphaneous vein tissue

113. Rotchell, J.M., Jenner, L.C., Chapman, E., Bennett, R.T., Bolanle, I.O., Loubani, M., Sadofsky, L., Hobkirk, J. and Palmer, T.M., 2023. Detection of microplastics in human saphenous vein tissue using μFTIR: A pilot study. *PLoS One*, *18*(2), p.e0280594.

Skin, Hair (head), Nasal Hair, Saliva

114. Shahsavaripour, M.; Abbasi, S.; Mirzaee, M.; Amiri, H. Human Occupational Exposure to Microplastics: A Cross-Sectional Study in a Plastic Products Manufacturing Plant. *Sci. Total Environ.* **2023**, *882*, 163576.

115. Abbasi, S.; Turner, A. Human Exposure to Microplastics: A Study in Iran. *J. Hazard. Mater.* **2021**, *403*, 123799.

Aqueous humor, Vitreous humor

116. Zhang, K.; Yu, L.; Qu, L.; Hui, N.; Chen, L.; Wang, J.; Yan, H. Identifying and Analyzing the Microplastics in Human Aqueous Humor by Pyrolysis-Gas Chromatography/Mass Spectrometry. *iScience* **2025**.

117. Zhong, Y.; Yang, Y.; Zhang, L.; Ma, D.; Wen, K.; Cai, J.; Cai, Z.; Wang, C.; Chai, X.; Zhong, J.; Liang, B.; Huang, Y.; Xian, H.; Li, Z.; Yang, X.; Chen, D.; Zhang, G.; Huang, Z. Revealing New Insights: Two-Center Evidence of Microplastics in Human Vitreous Humor and Their Implications for Ocular Health. *Sci. Total Environ.* **2024**, *854*, 158710.

Tear fluid/meibum

118. Wang, J.; Kang, H.; Huang, X.; Liu, Y.; He, Y.; Jie, Y. Identification of Microplastics in Human Tear Fluid and Meibum: Implications for Dry Eye Disease Pathogenesis. *J. Hazard. Mater.* **2025**, *489*, 137635.

Breast Milk

119. Saraluck, A.; Techarang, T.; Bunyapipat, P.; Boonchuwong, K.; Pullaput, Y.; Mordmuang, A. Detection of Microplastics in Human Breast Milk and Its Association with Changes in Human Milk Bacterial Microbiota. *J. Clin. Med.* **2024**, *13* (14), 4029.

120. Liu, S.; Guo, J.; Liu, X.; Yang, R.; Wang, H.; Sun, Y.; Chen, B.; Dong, R. Detection of Various Microplastics in Placentas, Meconium, Infant Feces, Breastmilk and Infant Formula: A Pilot Prospective Study. *Sci. Total Environ.* **2023**, *854*, 158699.

Human cumulus granulosa cells

121. Weina, L.; Yena, H.; Liang, H.; Huan, Z.; Gang, L.; Ge, L. P-762 Discovery and Quantification of Microplastics in Human Cumulus Granulosa Cells. *Hum. Reprod.* **2024**, *39*(Suppl_1), deae108–1081.

Endometrium

122. Sun, J.; Sui, M.; Wang, T.; Teng, X.; Sun, J.; Chen, M. Detection and Quantification of Various Microplastics in Human Endometrium Based on Laser Direct Infrared Spectroscopy. *Sci. Total Environ.* **2024**, *906*, 167760.

Pericardi, Epicardial adipose tissue, Pericardial adipose tissue, Myocardia, Left atrial appendage Pre/postoperative venous blood

123. Yang, Y., Xie, E., Du, Z., Peng, Z., Han, Z., Li, L., Zhao, R., Qin, Y., Xue, M., Li, F. and Hua, K., 2023. Detection of various microplastics in patients undergoing cardiac surgery. *Environmental Science & Technology*, *57*(30), pp.10911-10918.

Table S1. Microplastics in Human Tissues: Comprehensive Findings

Organ	Polymers Identified	Sample Type	Size Range (μm)	Concentration	Key Health Risks	Patient Demographics	Occupational & Habitat Factors	MP-Biomarker Correlation	Reference
Stomach	Polyethylene (PE), Polypropylene (PP), Polyethylene Terephthalate (PET)	Stomach content	Not specified	9.4 ± 10.4 per individual	Uncertain, but potential for digestive inflammation or disruption	26 cadavers, age range not specified			Özsoy et al. (2024)
Bone	Polypropylene (PP), Ethylene Vinyl Acetate (EVA), Polystyrene (PS)	Tissue	25.44–407.39; fragments	22.9 ± 15.7 particles/g	Elevated inflammatory markers (TNF-α), increased bone turnover indicators (PINP, TRACP-5b)	Age: 45-78 years, 60% male, 40% female		MPs correlated with increased TNF-α (inflammation), PINP (bone formation), TRACP-5b (bone resorption)	Yang et al. (2025)
Cartilage	PP, EVA, PS	Tissue	25.44–407.39	26.4 ± 17.6 particles/g	Elevated inflammatory markers (TNF-α), increased bone turnover indicators (PINP, TRACP-5b)	Age: 45-78 years, 60% male, 40% female			
Intervertebral Disc	PP, EVA, PS	Tissue	25.44–407.39	61.1 ± 44.2 particles/g	Elevated inflammatory markers (TNF-α), increased bone turnover indicators (PINP, TRACP-5b)	Age: 45-78 years, 60% male, 40% female			
Skin (Hands, Face)	Polyester, Nylon	Surface Swabs	100–5,000	High, especially on hands; 76.7 ± 15.7 - 165.77 ± 35.9 (mean)	Potential dermal irritation or transdermal absorption	19 workers, mixed gender	Plastic manufacturing plant	Not assessed	Shahsavaripour et al. (2023)
Saliva	Polyester, Nylon	Saliva samples	100–5,000	Lowest MP concentration	Potential ingestion and respiratory exposure	19 workers, mixed gender	Plastic manufacturing plant	Not assessed	
Hair	Polyester, Nylon	Hair samples	100–5,000	Highest MP concentration	Surface adherence, potential inhalation risk	19 workers, mixed gender	Plastic manufacturing plant	Not assessed	
Lung Tumors	Polystyrene (PS), Polyvinyl Chloride (PVC), Polyethylene (PE)	Tumor Tissue	Not specified	7.1–545.9 ng/g; detection rate: 80%	Potential alteration of tumor immune microenvironment (TIME); reduced CD8⁺ T cells, natural killer cells, dendritic cells; increased neutrophil infiltration	Not specified	Not specified	MPs associated with immunosuppressive TIME features, potentially affecting tumor progression and response to immunotherapy	Zhao et al., 2024
Pancreatic Tumors	PS, PVC, PE	Tumor Tissue	Not specified	18.4–427.1 ng/g; detection rate: 70%	Similar potential risks as lung tumors	patients aged between 18 and 75 years and diagnosed pathologically with one of the following malignant tumors: lung cancer, cervical cancer, gastric	Not specified	MPs associated with immunosuppressive TIME features, potentially affecting tumor progression and response to immunotherapy	

						cancer, colorectal cancer, esophageal cancer, or pancreatic cancer			
Gastric Tumors	PS, PVC, PE	Tumor Tissue	Not specified	7.1–545.9 ng/g; detection rate: 40%	Not specifically detailed in the study		Not specified	Not specifically detailed in the study	
Colorectal Tumors	PS, PVC, PE	Tumor Tissue	Not specified	7.1–545.9 ng/g; detection rate: 50%	Not specifically detailed in the study		Not specified	Not specifically detailed in the study	
Cervical Tumors	PS, PVC, PE	Tumor Tissue	Not specified	7.1–545.9 ng/g; detection rate: 17%	Not specifically detailed in the study		Not specified	Not specifically detailed in the study	
Esophageal Tumors	None detected	Tumor Tissue	Not applicable	Not applicable	No MPs detected in these samples		Not specified	Not applicable	
Cervix (Cancerous Tissue)	Polyethylene (PE), Polypropylene (PP), Polyethylene-co-Polypropylene (PE-co-PP), Cotton, polyethylene vinyl acetate, polyester,	Tumor Tissue	Length: 6.42–63.57 µm<br><br>Width: 4.04–64.29 µm	Higher than paracancerous tissues; 1.87 ± 1.02 MP particles per g	Potential reproductive health implications; possible influence on tumor microenvironment	15 patients with cervical cancer, between the ages of 37 and 69	Not specified	Positive correlation between BMI and MP abundance in cancerous tissues	Xu et al., 2024
Cervix (Paracancerous Tissue)	PE, PP, PE-co-PP, Cotton	Adjacent Non-Tumor Tissue		Lower than cancerous tissues; 0.87 ± 0.72 MP particles per g	Potential reproductive health implications; possible influence on tumor microenvironment		Not specified		
Blood	PE, PP, PE-co-PP, Cotton	Blood Samples	Length: 20.03 µm and the width was 15.93 µm	Higher diversity and abundance than tissues	Systemic exposure concerns; potential for distribution to various organs		Not specified	Positive correlation between age and MP abundance in blood; increased MP levels associated with frequent bottled beverage consumption	
Prostate	Polyamide, PET, PVC, Polystyrene (only in tumor tissue)	Tumor Tissue	20–100	290.3 particles/g	Not specified	22 patients undergoing RARP at Peking University First Hospital (Jan 2023–Jul 2024)	Take-out food consumption positively correlated with polystyrene presence in tumor tissues	Polystyrene showed significantly higher abundance in tumor tissues	Deng et al., 2024
Prostate	Polyamide, PET, PVC, PP, PE	Para-tumor Tissue	20–100	181.0 particles/g	Not specified	22 patients undergoing RARP at Peking University First Hospital (Jan 2023–Jul 2024)	Not specified	MPs detected in all 20 paired prostate tissue samples	
Penis	PE, PP, PVC (most prevalent) + 6 other MPs	Cancerous Tissue (CT)	20–50	6.42 particles/g; 85.3 % of the samples analyzed	Not specified	17 patients diagnosed with penile cancer	Not specified	MPs were more abundant and diverse in CT compared to ANT	Wang et al., 2025
Penis	PE, PP, PVC (most prevalent) + 6 other MPs	Adjacent Normal Tissue (ANT)	20–50	6.42	Not specified	17 patients diagnosed with penile cancer	Not specified	MPs in ANT had comparable size distributions but lower abundance	
Feces	Polyethylene Terephthalate (PET), Polyamide/Nylon,	Stool	0.2–4.9 mm	4 to 21 per 25g	Not specified	30 healthy pregnant women from	Significant variation in MP	Not specified	Hasanah et al., 2024

	Polyethylene Chlorinated, High-Density Polyethylene (HDPE), Ethylene Propylene					Makassar City, Indonesia	amounts among groups with different levels of seafood consumption		
Ileal Segments & Mesenteric Adipose Tissue (MAT)	Chlorinated polyethylene (CPE), Acrylate copolymer (ACR), Fluororubber, Polyurethane (PU), Polyethylene (PE)	Fibrotic ileal segments, creeping fat (CF), uninvolved ileal segments, adjacent mesenteric adipose tissue (CD-MAT)	20–50	Higher in fibrotic intestines and CF; ratio of PU & AUR increased, CPE & Fluororubber decreased in fibrotic regions<br><br>MPs were detected in all samples with average abundance of 2.44 particles/g in non-stenosis intestines, 8.59 particles/g in adjacent mesenteric fat, 1.41 particles/g in stenosis intestines, and 8.05 particles/g in the CF group, respectively	Fibrosis progression, increased MPs in high-risk areas, potential role in creeping fat development	10 Crohn's disease (CD) patients	Frequent gastrointestinal tract procedures exacerbated MPs accumulation	Positive correlation with fibrosis severity; MPs penetrate epithelial barriers and accumulate in fibrotic intestines and CF	Wu et al., 2025
Urinary System	Polyethylene (PE), Polypropylene (PP), Polyethylene terephthalate (PET), Polystyrene (PS)	Urine samples	4–15	Female: 0 - 2 particles/mL<br>Male: 0 – 3 particles/mL<br>Fragment	Potential renal stress, urinary tract inflammation, systemic exposure via excretion	6 adult volunteers (preliminary study)	Environmental and dietary exposure suspected	Not assessed in this study	Pironti et al., 2022
Kidney Tissue	Polyethylene (PE), Polystyrene (PS)	Healthy kidney tissue sections	1–29	26 microparticles identified across 10 kidney samples	First evidence of MP deposition in human kidney tissues.	Patients undergoing nephrectomies	Not specified	Not assessed	Massardo et al., 2024
Urine	Polyethylene (PE), Polystyrene (PS)	Urine samples from healthy donors	3–13	26 microparticles identified across 10 urine samples	Confirms presence of MPs in human urine, indicating renal excretion as a potential elimination route.	Healthy adult donors	Not specified	Not assessed	
Liver (Cirrhotic)<br><br>Kidney<br><br>Spleen	Six different MP polymers	Liver tissue from cirrhotic patients	4–30	1.2 particles per gram tissue<br><br>0.0 particle per gram	MPs detected in cirrhotic liver tissues; no MPs found in liver, kidney, and spleen samples from non-cirrhotic individuals.	6 patients with liver cirrhosis	Not specified	MP concentrations in cirrhotic liver tissues tested positive and showed significantly higher concentrations compared to liver samples of individuals without underlying liver disease.	Horvatits et al., 2022
Reproductive System	Polystyrene (PS), Polypropylene (PP), Polyethylene (PE), Polytetrafluoroethylene (PTFE), Polyethylene	Semen and Urine	Not specified	Not specified	Decreased sperm count, sperm concentration, and progressive motility associated with PTFE exposure. Each	113 male participants from three regions in China	Not specified	Significant association between PTFE exposure and reduced semen quality (total sperm count, sperm concentration, progressive	Zhang et al. (2024)

	Terephthalate (PET), Acrylonitrile Butadiene Styrene (ABS)				additional type of microplastic exposure was significantly associated with a decrease in sperm quality.			motility). Each additional type of microplastic exposure negatively correlated with semen quality (β = −15.4, −7.2, −8.3 for sperm count, concentration, and motility, respectively).	
Reproductive System	Polyethylene (PE), Polyvinyl Chloride (PVC), Polyethylene Terephthalate (PET), and 9 other types	Testicular Tissue	Not specified	Dogs: 122.63 µg/g; Humans: 328.44 µg/g	Negative correlation between PVC and PET levels and testis weight observed.	47 dogs and 23 humans; specific demographics not detailed	Not specified	Significant presence of microplastics in testicular tissue; higher PVC levels associated with lower sperm count in dogs.	Hu et al. (2024)
Reproductive System	Polypropylene (PP), Polyethylene (PE), Polyethylene Terephthalate (PET), Polystyrene (PS), Polyvinylchloride (PVC), Polycarbonate (PC), Polyoxymethylene (POM), Acrylic	Semen	2–6	16 microplastic fragments found in 6 out of 10 samples	Potential alterations in reproductive function due to microplastic presence in semen	Men residing in a polluted area of the Campania Region, Southern Italy	Not specified	Detection of microplastics in human semen suggests possible exposure through ingestion and/or inhalation. Proposed mechanism indicates MPs may enter semen via the epididymis and seminal vesicles, which are susceptible to inflammation.	Montano et al. (2023)
Male Reproductive System	Polystyrene (PS)	Semen	0.025–10	3.57 ± 0.32 µg/mL	Reduced sperm motility and viability upon exposure to 25-nm PS nanoplastics at 5 and 50 µg/mL concentrations	Not specified	Not specified	Exposure to 25-nm PS nanoplastics at 5 and 50 µg/mL concentrations reduced sperm motility and viability. Magnetic iron oxide nanoparticles can aggregate and coprecipitate with PS nanoplastics, mitigating their adverse effects.	Chen et al. (2024) (springer.com)
Liver and feces	Not specified	Feces	Not specified	0 to 4.7 particles/g DW, with a mean of 0.9 +/− 0.2 MP particles/g; 0.6 +/− 0.2 fibers/g DW and 0.3 +/− 0.1 fragments/g DW	Association between fecal microplastics (MPs) and immune cell counts, gene expression related to immune response and apoptosis, and intestinal microbiota composition.	6 lean healthy liver donors, 6 obese individuals with normal liver histology, and 11 patients with metabolic dysfunction-associated steatotic liver disease (MASLD).	Not specified	Positive correlation between fecal MP fibers and portal macrophages, total macrophages, and killer T cells; positive correlation between total fecal MPs and natural killer cells. Additionally, 19 genes related to immune system and apoptosis correlated with fecal MPs at baseline. Fecal MP fibers correlated positively with Bifidobacterium and negatively with Lachnospiraceae.	Schwenger et al. (2024)

Amniotic Fluid	Polyethylene (PE), Chlorinated Polyethylene (CPE)	Amniotic Fluid	20 – 100	2.01 ± 4.19 particles/g; 32 out of 40	Potential fetal health risks due to MP exposure	40 pregnant women (mean age 30.18 ± 2.35 years) undergoing acute cesarean sections	Not specified	Positive correlation between MP levels and frequency of seafood consumption (r = 0.781, P < 0.001) and bottled water intake (r = 0.386, P = 0.014). Negative association between MP levels and gestational age (β = -0.44, 95% CI: -0.83, -0.05)	Xue et al. (2024)
Placenta	Polyamide (PA), Polyurethane (PU), PVC, PLA, PTFE	Placenta	20 - > 150	18.0 particles/g (max. 61 particles/g)	Potential fetal exposure to MPs	18 pregnant women (mean age 30.18 ± 2.35 years)	Not specified	Positive correlation between MP levels and frequency of water intake and use of scrub cleanser or toothpaste among pregnant women.	Liu et al. (2023)
Meconium	PA, PU	Meconium	Not specified	51.4 particles/g	Potential infant exposure to MPs	Same cohort as above	Not specified	Positive correlation between MP levels and breastfeeding, use of feeding bottles, and use of plastic toys among infants.	
Infant Feces	PA, PU	Infant Feces	Not specified	26.6 particles/g	Potential infant exposure to MPs	Same cohort as above	Not specified	Positive correlation between MP levels and breastfeeding, use of feeding bottles, and use of plastic toys among infants.	
Breast Milk				20.2 particles/g					
Maternal Blood	Polyamide (PA), Polyurethane (PU)	Blood	20–100	8.176 particles/g	Potential maternal health risks due to MP exposure	12 full-term pregnant women (delivery via cesarean section)	Not specified	Positive correlation between MP abundance in amniotic fluid and maternal age (r = 0.64, p = 0.025) and pre-pregnancy BMI (r = 0.59, p = 0.049). No significant association with lifestyle factors.	Sun et al. (2024) (sciencedirect.com)
Fetal Membrane	PA, PU	Membrane	20–100	6.561 particles/g	Potential fetal health risks due to MP exposure	Same cohort as above	Not specified		
Umbilical Cord	PA, PU	Cord	20–100	10.397 particles/g	Potential fetal health risks due to MP exposure	Same cohort as above	Not specified		
Amniotic Fluid	PA, PU	Fluid	20–100	4.795 particles/g	Potential fetal health risks due to MP exposure	Same cohort as above	Not specified		
Placenta	PA, PU	Tissue	20–100	4.675 particles/g	Potential fetal health risks due to MP exposure	Same cohort as above	Not specified		
Amniotic fluid	Chlorinated Polyethylene (CPE), Calcium zinc PVC Stabilizer	Fluid	10–50	44 (total across samples); 9 out of 10 patients	Potential fetal exposure to microplastics and additives	Pregnant women with preterm prelabour rupture of	Not specified	Not specified	Halfar et al. (2023)

						membranes (PPROM)			
Placenta	Chlorinated Polyethylene (CPE), Calcium zinc PVC Stabilizer	Tissue	10–50	44 (total across samples)	Potential fetal exposure to microplastics and additives	Pregnant women with preterm prelabour rupture of membranes (PPROM)	Not specified	Not specified	Halfar et al. (2023)
Placenta	Polyethylene (PE), Polystyrene (PS), among others	Human Placenta	<5	2–38 particles per placenta (IUGR cases); below limit of detection in normal pregnancies, except for three subjects	Potential impact on fetal growth parameters, including birth weight, length, head circumference, and Apgar score	43 pregnant women from the general population, including 13 with IUGR	Not specified	Significant inverse correlations observed between MP exposure and birth outcomes in IUGR cases	Amereh et al. (2022)
Placenta	Polypropylene (PP) and various pigments	Human Placenta	5–10	12 microplastic fragments found across 6 placentas; 5 in fetal side, 4 in maternal side, 3 in chorioamniotic membranes	Potential endocrine disruption due to associated pigments	6 consenting women with physiological pregnancies	Not specified	No direct correlation assessed	Ragusa et al. (2021)
Carotid Artery Plaque	Polyethylene (PE), Polyvinyl Chloride (PVC)	Carotid Plaque Tissue	Not specified	PE: Mean 21.7 ± 24.5 µg/mg of plaque; PVC: Mean 5.2 ± 2.4 µg/mg of plaque	Increased risk of myocardial infarction, stroke, and all-cause mortality	304 patients undergoing carotid endarterectomy for asymptomatic carotid artery disease; mean follow-up of 33.7 ± 6.9 months	Not specified	Significant association between MNP presence and higher risk of cardiovascular events	Marfella et al. (2024)
Brain	Not specified	Brain Tissue	Not specified	2016: 3345 µg g−1 (25–75%: 1,267–5,213); 2024: 4917 µg g^{-1} (25–75%: 4,026–5,608)	Potential neurological effects, association with dementia	52 deceased individuals; samples from 2016 and 2024 autopsy specimens	Not specified	Higher MNP concentrations in dementia cases and recent samples	Nihart et al. (2025)
Liver	Not specified	Liver Tissue	Not specified	2024: 433 µg/g (median)	Potential liver damage and metabolic disruptions	Autopsy samples from 2016 and 2024	Not specified	Higher concentrations in 2024 samples	
Kidney	Not specified	Kidney Tissue	Not specified	2024: 404 µg/g (median)	Potential kidney damage, endocrine disruption	Autopsy samples from 2016 and 2024	Not specified	Higher concentrations in 2024 samples	
Olfactory Bulb	Polypropylene (43.8%)	Olfactory Bulb Tissue	5.5–26.4	1 to 4 MPs per OB; 12 males, 3 females; 75% being particles and 25% being fibers; 8 out of the 15 deceased individuals	Potential neurotoxicity, implications for brain health	15 deceased individuals; median age 69.5 years; age range 33–100 years; 12 males, 3 females	Not specified	Detection in 8 out of 15 samples	Amato-Lourenço et al. (2024)
Human Feces	Polyethylene Terephthalate (PET), Polyamide (PA), and others (15 total types)	Fecal Samples	Not specified, likely ranging from a few µm to larger sizes	IBD patients: 41.8 items/g dry matter (dm); Healthy people: 28.0 items/g dm	Inflammatory Bowel Disease (IBD), potential exacerbation of IBD	Patients with Inflammatory Bowel Disease (IBD), Healthy individuals for comparison	Plastic packaging of food and drinking water, dust exposure	Positive correlation between fecal MPs and IBD severity, suggesting MPs may be related to the disease process or worsen IBD	Yan et al. (2021)

Human Lung Tissue	Polyethylene (PE), Polypropylene (PP), others (Polymeric particles and fibers)	Lung Tissue Samples	Polymeric particles: < 5.5 µm; Fibers: 8.12 - 16.8 µm	33 polymeric particles and 4 fibers detected in 13 of 20 samples	Respiratory inflammation, potential lung damage, chronic respiratory diseases	Individuals exposed to airborne pollutants, urban populations	Inhalation of airborne microplastics from urban pollution, industrial emissions, traffic	Presence of MPs in lung tissue suggests potential adverse respiratory effects, further studies needed	Amato-Lourenço et al. (2021)
Lung	Polypropylene (PP), Polyethylene Terephthalate (PET), Resin, among others	Peripheral human lung tissue samples from upper, middle (left lingula), or lower lobes following surgical resection for cancer or lung volume reduction surgery	MPs ≥ 3 µm (µFTIR spectroscopy detects MPs ≥ 3 µm); 18 fragment; 18 fibre; 1 film	Average: 1.42 ± 1.50 MP/g of tissue; After background subtraction: 0.69 ± 0.84 MP/g	Inhalation-related respiratory health risks	Patients undergoing surgery for cancer or lung volume reduction surgery	Not specified	Not specified	Jenner et al. (2022)
Lung	Microplastics (various types), including microfibers	Lung tissue samples from individuals with ground glass nodules (GGNs) and normal tissue	Microplastics > 20 µm	65 microfibers (including 24 microplastics) in 100 samples; higher detection in tumor tissues	Inhalation-related risks, potential contribution to GGN formation	Patients with lung ground glass nodules (GGNs)	Increased microfiber exposure linked to higher detection rates	Increased surface roughness of fibers in lung tissues suggests a role in disease progression	Chen et al. (2022)
Respiratory Tract (Sputum)	Polyurethane (PU), Polyester, Chlorinated Polyethylene, Alkyd Varnish (78.36% of total microplastics)	Human sputum samples from healthy and diseased individuals	20 to 500 µm (median: 75.43 µm)	Microplastics detected in all sputum samples, with varying concentrations based on individual factors (e.g., smoking); 120.136 particles/10mL	Inhalation of microplastics may cause respiratory issues	Healthy and diseased individuals, including smokers	Smoking and invasive medical procedures were linked to higher microplastic presence	Not specified	Huang et al. (2022)
Respiratory System (Sputum, BALF, Pleural Fluid)	Polyester (62–72%) and 21 other types of MPs	13 sputum samples, 11 BALF samples, 10 pleural fluid samples from 34 patients	<100 µm particles were most abundant (sputum 75.9%, BALF 50%, pleural fluid 94.4%)	Sputum: 156–468 particles/100 mL, BALF: 28–133 particles/100 mL, Pleural fluid: 21–210 particles/100 mL	Respiratory issues related to microplastic inhalation, deposition in the lungs	34 patients, including smokers and those with various diseases	Occupational exposure, smoking, exposure to cigarette smoke, and mask-wearing affected MPs abundance	Shape and size of MPs affect their deposition in the respiratory system ($P < 0.05$)	Momeni et al. (2025)
Respiratory System (BAL)	Polyamide (PA), Polyester (PET), Polyvinyl Chloride (PVC), Polyurethane (PU)	Bronchoalveolar lavage (BAL) samples	4.19 to 792.00	10 out of 18 patients; not specified	Potential respiratory inflammation and fibrosis	Patients suspected of ILD (18 total)	Not specified	Not specified	Alpaydin et al. (2024)
Ovarian Follicular Fluid	Not specified	Follicular fluid samples	<10	Average: 2191 (range: 0–7181) particles/mL; 14 out of 18 samples	Potential impact on female fertility	18 women undergoing assisted reproductive treatment	Not specified	Significant correlation with FSH levels ($p < 0.05$)	Montano et al., 2025
Semen (Seminal Fluid)	Polystyrene (31%), Polyethylene (14%), Polyvinyl Chloride (14%), Acrylonitrile-Butadiene-Styrene (11%), Polycarbonate (11%), Polyethylene Terephthalate (8%), Polypropylene (6%),	Semen samples from 40 individuals undergoing premarital health assessments	0.72 to 7.02	2 (range: 0–7) per sample	Potential effects on sperm motility and morphology	40 individuals (age not specified) from Jinan, China	No occupational exposure to microplastics	Exposure to polystyrene associated with higher sperm progressive motility compared to polyvinyl chloride exposure	Li et al. (2024)

	Polytetrafluoroethylene (6%)								
Tear Fluid & Meibum	PE, PVC, PS, PP, PC	Tear fluid and meibum	Not specified	Not specified	Potential impact on dry eye disease (DED), including tear film instability, reduced tear secretion	21 males, 24 females, age range 25–78 years	Not specified	Tear Fluid:<br>- Age vs. PE: $r^2 = 0.741$, $P = 0.003$<br>- Age vs. PVC: $r^2 = 0.452$, $P = 0.047$<br>- PE/PVC vs. tear secretion volume: $r = -0.728$, $P = 0.026$ (PE), $r = -0.723$, $P = 0.028$ (PVC)<br>- PE/PVC vs. FBUT: $r = -0.737$, $P = 0.023$ (PE), $r = -0.696$, $P = 0.037$ (PVC)<br><br>Meibum:<br>- PE vs. residual meibomian gland area: $r = 0.884$, $P = 0.002$<br>- PVC vs. residual meibomian gland area: $r = 0.705$, $P = 0.034$<br>- PE vs. lipid layer thickness: $r = -0.761$, $P = 0.017$<br>- PVC vs. partial blink rate: $r = 0.716$, $P = 0.030$	Wang et al. (2025)
Urine	PE, PP, PS	Urine	20–100 µm	9 MPs (small fibres) out of 8 patients	Potential ingestion route for MPs, risk of contamination from environmental sources	30 adult patients with respiratory conditions, Iran	Not specified	MPs detected: small (20–100 µm), green and red fibres; predominantly PE, PP, and PS.	Jahedi et al. (2025)
Sputum	Polyurethane (dominant), other polymers	Sputum	100–500 µm, fibres, fragments, spherules	358 MPs; 5 to 22 particles per participant	Risk of respiratory tract irritation, chronic lung disease risks with inhaled MPs	30 adult patients with respiratory conditions, Iran	Not specified	MPs detected: Dominated by small, white, transparent fibres, including larger fragments and spherules. Broad polymer variety, with polyurethane most common.	
BALF	Similar to urine samples (PE, PP, PS, etc.)	Bronchoalveolar Lavage Fluid (BALF)	Larger fibres, fragments, spherules	123 MPs; 1 to 15 particles per participant	Potential acute or chronic respiratory issues from inhaled MPs	30 adult patients with respiratory conditions, Iran	Not specified	MPs detected: Larger fibres, fragments, and spherules with colour similar to sputum. Polymer distribution more like urine samples.	
Feces	Polypropylene (PP), others	Fecal Samples	20–800 µm	1–36 particles/gram	Potential gastrointestinal and systemic health effects, environmental contamination	26 young male students, aged 18–25 years, from Beijing	Dietary habits, beverage consumption (water in plastic bottles)	Moderate positive correlation between packaged water/bottle beverage consumption and fecal MP abundance ($r = 0.445$, $P = 0.029$)	Zhang et al. (2021)

Lung (BALF)	Polypropylene (41.9%), Polyethylene (19.4%), Polyester (13.6%), others	BALF Samples	Predominantly <20 µm	Average: 4.31 ± 2.77 particles/10 mL	Potential respiratory issues; higher exposure linked to urban living and younger age	Children with respiratory diseases	Urban vs. suburban/rural residence	Negative correlation between age and MP levels; urban residents had higher MP exposure; severe pneumonia cases had higher MP contamination.	Chen et al. (2023)
Synovium (hip/knee)	PET (27.1%), PE (21.9%), Rayon (RA) (12.0%), Polyester (PES) (11.1%), Polypropylene (PP) (9.3%), Polyamide (PA) (8.5%), PVC (4.7%), Polystyrene (PS) (4.4%), Polycarbonate (PC) (2.0%)	Synovial Tissue	11.00 to 151.00 µm	Mean: 5.24 ± 2.07; Range: 1.16–10.77 particles/g	Inflammatory responses, potential joint dysfunction	45 patients undergoing hip or knee arthroplasty	Not specified	A three-fold increase in MP abundance linked to enhanced heat shock protein responses. Common exposure pathways for knee and hip joints.	Li et al. (2024)
Penis	PET (47.8%), PP (34.7%), others (17.5%)	Penile Tissue	2–500	80% of samples detected MPs; not specified	Potential impact on sexual health, including erectile dysfunction	6 patients undergoing IPP surgery	Not specified	Not assessed	Codrington et al. (2024)
Respiratory tract (BALF)	Polyethylene (PE; 86.1%), Poly(ethylene terephthalate) (PET; 7.5%), Polypropylene (PP; 1.9%), others (4.5%)	Bronchoalveolar lavage fluid	Not specified	median [IQR] of BALF: 0.38 [8.05] N/g), PET (0.26 [0.54] N/g), polyurethane (0.16 [0.24] N/g), PP (0.16 [0.11] N/g), and total MPs (0.91 [6.58] N/g)	Potential respiratory inflammation or other pulmonary issues	18 never-smokers, aged 32–74 years	Residents of Zhuhai, China	Not specified	Qiu et al. (2023)
Respiratory tract (BALF)	Polyurethane (PU), Silicone, others	Bronchoalveolar lavage fluid	Not specified	Smokers: Total MPs 25.86 particles/g; PU 11.34 particles/g; Silicone 1.15 particles/g	Potential respiratory inflammation or other pulmonary issues	17 smokers and 15 non-smokers from Zhuhai City, China	Not specified	Not specified	Lu et al. (2023)
Blood (thrombi)	Phthalocyanine pigments, Hostasol-Green pigment, low-density polyethylene microplastics	Thrombus	2.1–26.0	1–15 (median = 5) per thrombus; 16 out 26 positive	Potential contribution to thrombus formation and related cardiovascular issues	26 patients undergoing cardiovascular surgery	Not specified	Positive correlation between microparticle count and blood platelet levels ($P < 0.01$)	Wu et al. (2023)
Cerebral arteries	PA66, PVC, PE	Thrombus	Not specified	61.8 µg/g (median)	Association with ischemic stroke severity	10 patients with ischemic stroke	Not specified	Positive correlation between MP concentration and disease severity	Wang et al., 2024
Coronary arteries	PA66, PVC, PE	Thrombus	Not specified	141.8 µg/g (median)	Association with myocardial infarction severity	10 patients with myocardial infarction	Not specified	Positive correlation between MP concentration and disease severity	
Deep veins	PA66, PVC, PE	Thrombus	Not specified	69.6 µg/g (median)	Association with deep vein thrombosis severity	10 patients with deep vein thrombosis	Not specified	Positive correlation between MP concentration and disease severity	
Blood	Polystyrene, Polypropylene	Whole blood	Not specified	4.2 (mean) particles/mL(88% of participants tested positive)	Association with altered coagulation markers	36 healthy adults	Frequent use of plastic food containers	Positive correlation between MP load ≥3 MPs/mL and increased aPTT, CRP, and fibrinogen levels	Lee et al., 2024
Nasal Lavage Fluid	PE, PP, PS	Nasal Lavage Fluid	1–200 µm	12.5 ± 3.2 MPs/mL	Inhalation of microplastics may	50 participants (Shenyang, China)	Participants divided based on	Significant reduction in MP concentration for	Zhang et al. (2024)

					cause respiratory irritation and long-term lung damage		face mask use (non-users, occasional, regular users)	regular face mask users	
Breast Milk	Polypropylene (PP), Polyethylene (PE), Polystyrene (PS), Polyvinyl Chloride (PVC)	Postpartum breast milk	Not specified	38.98% of breast milk samples (23 of 59); 1 – 4 MPs per sample (10 – 15g)	Potential impact on infant health due to MP ingestion; alteration of breast milk microbiota	59 postpartum women (mean age 28.13 years)	Not specified	Significant differences in bacterial microbiota composition between MP-detected and non-detected groups; dominance of Staphylococcus and Streptococcus in MP-detected group	Saraluck et al., 2024
Placenta, Meconium	Polyamide (PA), Polyurethane (PU), Polystyrene (PS), Polyethylene (PE), and 12 other types	Human tissue, Meconium	20–50 µm, 50–100 µm	Not specified	Potential disruption of microbiota, fetal development, immune response	Pregnant women, infants (mother-infant pairs)	Environmental exposure (Shanghai, China)	Inverse correlation with Chao index in meconium microbiota; inverse correlation with genera in placenta microbiota	Liu et al., 2022
Aqueous Humor	Polyethylene (PE), Polyvinyl chloride (PVC), Polypropylene (PP), Polyamide 66 (PA66), Polystyrene (PS)	Aqueous humor (eye)	Not specified	Children: 7–20 µg/gram; Adults (Myopia): 5.5–22.3 µg/gram; Elderly (ARC): 3.7–13.38 µg/gram	Potential intraocular health risks, eye inflammation, and long-term ocular diseases	Children, Adults (with myopia), Elderly (with age-related cataract)	No specific occupational exposure mentioned	First evidence of MPs in aqueous humor, potential link to intraocular health risks	Zhang et al., 2025
Upper Respiratory Tract	Polyethylene (PE), Polypropylene (PP), Polystyrene (PS)	Nasal swabs, Throat samples, sputum	2 µm to 50 µm; mainly fibers	61.8 – 728.6 particles/g	Inflammation, respiratory distress, potential long-term respiratory diseases	50 workers (25 indoor, 25 outdoor)	Indoor and outdoor work environments (construction, manufacturing, office work)	Not specified, but likely linked to respiratory health	Jiang et al., 2022
Gallstones	PS, PE, PP, PET, EVA	Gallstones from 16 patients post-cholecystectomy	0.5, 5, and 50 µm	LDIR (per g) (PS: 5.25 ± 4.88, PE: 4.75 ± 6.22, PP: 0.56 ± 0.73, PET: 0.94 ± 1.73, EVA: 1.19 ± 2.32) Py-GCMS (mg/kg) (PS: 10.69 ± 10.56, PE: 3.56 ± 3.21, PP: 0.09 ± 0.14, PET: 0.18 ± 0.38, EVA: 0.04 ± 0.08)	Aggravation of cholelithiasis, potential increase in cholesterol-microplastic heteroaggregates, alteration of gut microbiota	Significantly higher microplastic content in patients <50 years of age	Not specified in the study	Higher affinity between microplastics and cholesterol than bilirubin	Zhang et al. (2024)
Vitreous Humor	Nylon 66, Polyvinyl Chloride (PVC), Polystyrene (PS)	49 vitreous samples from patients with ocular diseases	Predominantly <50 µm; largest particle size – 450 µm<br>Macular hole cases:<br>12.32 mg/kg PA66,<br>0.70 mg/kg PS, and 0.24 mg/kg PVC	LD-IR: 8543 particles scanned; 1745 identified as plastic particles	Intraocular pressure changes, aqueous humor opacities, retinopathy-related health risks	49 patients with ocular diseases (macular hole, macular epiretinal membrane, retinopathy, and rhegmatogenous retinal detachment)	Not specified, but ocular disease history noted	Correlation between MP levels, intraocular pressure, aqueous humor opacities, and retinopathy severity	Zhong et al. (2024)

			macular epiretinal membrane cases: 14.76 mg/kg PA66, 1.38 mg/kg PS, and 0.67 mg/kg PVC<br><br>rhegmatogenous retinal detachment cases: 11.63 mg/kg PA66, 0.83 mg/kg PS, and 0 mg/kg PVC<br><br>retinopathy cases: 9.14 mg/kg PA66, 0.81 mg/kg PS, and 0 mg/kg PVC						
Uterine Fibroids (UFs) and Myometrium	PE, PP, PE-co-PP, Cotton, PEVA, PA, ABS	16 tissue samples (7 UFs, 8 myometrium, 1 blank)	4.86–40.15 µm	UFs: 2.13 ± 1.17 particles/g tissue; Myometrium: 0.88 ± 0.78 particles/g tissue; Blank correction: UFs: 2.13 ± 1.17, Myometrium: 0.75 ± 0.83	Potential hormonal disruptions, risk for uterine fibroid development	40 patients with UFs (specific age range not provided)	Not specified	UFs had significantly higher MP levels than myometrium ($p < 0.05$). MPs correlated with fibroid size. MPs more abundant in diseased tissues ($p < 0.01$)	Xu et al. (2024)
				Diseased tissue: 2.63 ± 1.77 MP particles/g tissue; Normal tissue: 1.08 ± 0.93 MP particles/g tissue ($p < 0.01$)	Increased MP presence in UFs may indicate a connection to fibroid development	40 tissue samples (diseased and normal from each patient)	Higher detection rate in diseased tissue (75% vs. 65%)	MPs in UFs tissues statistically higher than in normal myometrium, MPs correlate with increased fibroid size in diseased tissues.	
				Average length of MPs: 15.14 µm (range: 4.86–40.15 µm), Average width: 10.26 µm (range: 2.79–22.79 µm)	Risk of fibroid growth, potential long-term impact of chronic MP exposure	Diverse range of UFs (macroscopic fibroids)	Exposure may vary due to diet, environmental pollution	MPs in diseased tissue statistically higher compared to normal tissue ($p < 0.01$). Fibers and fragments most common forms of MPs	
Bone Marrow	PE, PS, PVC, PA66, PP	Bone Marrow	<100 µm	15.37–92.05 µg/g (avg. 51.29 µg/g)	Potential disruption to hematopoietic system	16 bone marrow samples	Not specified	LD-IR identified 25 polymer types, 89.82% <100 µm, average 19.72 particles/g	Guo et al. (2024)
Nasal Cavity	PE, Polyester, Acrylic Polymer, PP	NH, IT, MT, NF, MNCF	Width: 29.43 ± 15.49 µm, Length: 57.14 ± 51.98 µm	390 MPs from 10 nasal samples (Average size: 29.43 ± 15.49 µm in width, 57.14 ± 51.98 µm in	Not specified	Not specified	Not specified	Not specified	Min et al. (2024)

				length)					
Cumulus Granulosa Cells	Polyethylene Terephthalate (PET)	Cumulus Cells	Not specified	313.14 ± 246.69 mg/kg	Not specified	Not specified	Not specified	Not specified	Weina et al. (2024)
Follicular Fluid	Polyethylene Terephthalate (PET)	Follicular Fluid	Not specified	12.88 ± 15.54 mg/kg	Not specified	Not specified	Not specified	Not specified	
Testis	Polystyrene (PS)	21.76–286.71	11.60 ± 15.52 particles/g	Fragment (dominant), fiber, film	Not specified	Potential reproductive health risks	Not specified	Not specified	Zhao et al. (2024)
Semen	Polyethylene (PE), Polyvinyl Chloride (PVC)	Not specified	0.23 ± 0.45 particles/mL	Fiber, Film, Fragment	Not specified	Potential reproductive health risks	Not specified	Not specified	
Cervicovaginal area	Polypropylene, Polystyrene	Human cervicovaginal lavage fluid	<50 µm (majority)	9.10 ± 14.96 per 10 g sample	Potential reproductive health impact	10 participants, aged 27–49	Use of menstrual cups by some patients	Not specified	Shim, Y., & Min, H. (2025).
Lower airway (lung)	Polyacrylic (fiber)	Bronchoalveolar lavage fluid	1.73 ± 0.15 mm (longest)	9.18 ± 2.45 items per 100 mL of BALF (microfibers)	Potential respiratory and health impact	44 European adults	Not specified	Not specified	Baeza-Martínez et al. (2022)
Urinary system	Polyethylene (PE), Polystyrene (PS), Resin, Polypropylene (PP), Polytetrafluoroethylene (PTFE)	Human urine	Not specified	Healthy donors: 2,589 ± 2,931 MPs/L, Endometriosis: 4,724 ± 9,710 MPs/L	Potential health impact from microplastics in the urinary system	29 healthy donors, 19 endometriosis participants	Not specified	Not specified	Rotchell et al. (2024)
Meconium	Polyethylene (PE), Polypropylene (PP), Polystyrene (PS), Polyurethane (PU)	Placental tissue, Meconium	>50 µm	Not specified	Potential impact on fetal development, contamination risks	Collected during breech cesarean section	Operating room airborne fallout of polyurethane detected	Not specified	Braun et al. (2021)
Placenta	Rayon, Polystyrene (PS), Polyethylene (PE), Polyvinyl chloride (PVC), Nylon, and other 9 polymers	Placental tissue	>1 micron	6.5 to 685 µg NMPs per gram of placental tissue, averaging 126.8 ± 147.5 µg/g	Potential impact on maternal and fetal health, possible links to adverse pregnancy outcomes	62 placenta samples analyzed	Not specified	Not specified	Garcia et al. (2024)
Endometrium	Ethylene-acrylic acid copolymer, Polyethylene (PE), and others	Endometrial tissue	20–500 µm	0 to 117 particles per 100 mg of tissue, median 21 particles per 100 mg	Potential links to reproductive health concerns, impact on endometrial function	13 samples analyzed	Drinking habits, chewing gum consumption associated with higher MP exposure	Not specified	Sun et al. (2024)
Stools	Polyethylene (PE), Polyvinyl chloride (PVC), Polystyrene (PS), Polypropylene (PP), Polyethylene terephthalate (PET), Polyamide 66 (PA66)	Stool samples	Not specified	Total mass concentration: 54.7 (10.1–102.7) µg/g	Potential long-term health risks from ingestion and exposure	78 young college students from Changsha, Shanghai, and Changchun, China	Frequent takeaway food consumption, reheated food, bottled water intake significantly associated with higher MP concentrations	Not specified	Song et al. (2025)
Stool	Polyethylene (PE) and others	Stool samples	5–5000 µm	Up to 3.5 particles/g stool in the 50–500 µm size fraction	Potential digestive and long-term health risks from microplastic exposure	15 volunteers, pilot study	Use of plastics for food packaging and preparation, highly processed food consumption	Not specified	Hartmann et al. (2024)

							significantly linked to higher microplastic content		
Bronchioalveolar Lavage Fluid (BALF)	Not specified (but identified as micro- and nanoplastic particles)	BALF samples (patients undergoing diagnostic bronchoscopy)	Nano-sized and micro-sized particles, fragmented form	0.14–12.8 particles per 100 ml of BALF	Potential respiratory health risks, unknown effects on other organs	Clinical trial subjects undergoing bronchoscopy	Residents of Northern Europe, assumed exposure to airborne MP/NPs	Not specified	Uogintė et al. (2023)
Lung tissue	Polypropylene (PP), Polyethylene Terephthalate (PET), Polystyrene (PS), 9 other types	Lung tissue samples	20–100 µm (88.89% of MPs)	Median concentration of 2.19 particles/g	Potential respiratory health risks, inflammation, and oxidative stress	12 nonsmoking patients	Females and individuals living near major roads (<300m) had higher levels of MPs in lung tissue	Positive correlation with platelet (PLT), thrombocytocrit, fibrinogen (FIB); Negative correlation with direct bilirubin (DB)	Wang et al. (2024)
Cardia tissues (pericardium, epicardial adipose tissue, myocardium, left atrial appendage)	Poly(methyl methacrylate) (PMMA), other unidentified types	Tissue samples from patients undergoing cardiac surgery	Largest particle: 469 µm	Not all tissues contained MPs;	Potential health risks from internal exposure to MPs, inflammation, and other adverse effects	15 patients undergoing cardiac surgery	Surgical procedure-related exposure to MPs	Not specified	Yang et al. (2023)
Synovial Fluid	Polyethylene (PE), Polypropylene (PP), Polystyrene (PS), Titanium Dioxide (TiO_2)	Synovial fluid samples	Not specified (microparticles detected)	8 out of 10; Not specified in terms of concentration	Joint inflammation, potential implant-related complications	10 patients with suspected implant-related complications	Suspected implant-related complications	Not specified	Niessink et al. (2025)
Meconium	None identified	Meconium samples	≥10 µm	No detectable microplastics	Potential fetal exposure to microplastics (no detectable microplastics)	37 newborns (meconium samples)	No specific habitat or occupational factors mentioned	No correlation found	Li et al. (2023)
Blood	Polyethylene (32%), Ethylene propylene diene (14%), Ethylene-vinyl acetate/alcohol (12%), 21 other polymers identified	Whole blood samples	7–3000 µm in length, 5–800 µm in width	2466 ± 4174 MPs/L (mean ± SD), concentrations from 1.84 to 4.65 µg/mL	Vascular inflammation, organ buildup, immune response changes, thrombosis risks, endocrine disruption (phthalates identified)	20 healthy volunteers	Not specified	Not specified	Leonard et al. (2024)
Blood	Polyethylene Terephthalate (PET), Polyethylene (PE), Polystyrene (PS), Poly(methyl methacrylate) (PMMA)	Whole blood samples	≥700 nm (nanoplastic particles)	1.6 µg/mL (mean concentration)	Potential bioaccumulation, systemic exposure, and health impacts (vascular inflammation, immune response)	22 healthy volunteers	Not specified	Not specified	Leslie et al. (2022)
Human Blood	Polyvinyl chloride (PVC), Polyethylene (PE), Polypropylene (PP), Polystyrene (PS), Polyamide 66 (PA66)	Blood samples from 39 adults	Not specified (Polymer size range)	PE: 0–96.2 µg/mL, PVC: 0–62.4 µg/mL, PS: 0–4.2 µg/mL, PP: 0–7.4 µg/mL, PA66: 0–39.4 µg/mL	Potential immune system activation, microbiome dysbiosis, gastrointestinal disorders, systemic inflammation	39 adults, with observed differences between sexes for PA66 concentrations (higher in females)	Environmental and dietary factors likely contributors to exposure	Positive correlation with Enterobacteriaceae and Escherichia coli; Negative correlation with Faecalibacterium prausnitzii and Faecalibacterium genus. No significant difference in alpha and beta diversity between sexes or exposure	Gao et al. (2025)

								groups.	
Feces	Polypropylene (PP), Polyethylene (PE), Polyvinyl chloride (PVC), Polystyrene (PS), Polyamide (PA66)	Human stool, Daily consumables (table salt, toothpaste)	Varies: < 100 µm	Stool: 0–96.2 µg/mL (PE), 0–62.4 µg/mL (PVC), 0–4.2 µg/mL (PS), 0–7.4 µg/mL (PP), 0–39.4 µg/mL (PA66)	Potential gastrointestinal disruptions, impacts on gut microbiota, carriers for toxic substances	Adults from an Indonesian farming community	Farming, dietary consumption habits	Positive correlation with unclassified Enterobacteriaceae and E. coli; negative correlation with Faecalibacterium prausnitzii	Wibowo et al., 2021,
Feces, Urine	PE, PVC, PET, PP	Feces, Urine	0.22 µm to >20 µm	Feces: 345.58 µg/g, Urine: 6.49 µg/mL	Small-sized microplastics were more abundant than large-sized particles. Feces was the primary excretion route.	Not provided	Not provided	Not provided	Zhu et al., 2024
Stool	PVC, PET, PE, PA6	Stool samples	Not specified	PVC: 317.4 (152.0, 491.9) µg/g dw, PET: 299.0 (196.1, 619.9) µg/g dw, PE: 206.2 (154.1, 240.3) µg/g dw, PA6: 17.9 (13.4, 18.6) µg/g dw	Disturbance in gut microbiota, potential effects on probiotics (Parabacteroides, Alistipes)	Preschool children (Xiamen, China)	Dairy intake, usage of feeding bottles, meal duration	Inverse association with alpha diversity indices, possibly affects probiotics	Ke et al. (2023)
Kidney, Urine	Polystyrene, Styrene-Isoprene, Polyethylene, Hematite, Cu-phthalocyanine blue, Cerulean Blue	Kidney tissue, Urine samples	Not specified	1.7 ± 2.11 fragments/sample (kidney), 1.28 ± 0.49 fragments/sample (urine)	Potential kidney damage, urinary system impact	Healthy individuals, nephrectomy patients with renal cancer	Not specified	Significant presence of microplastics compared to controls, potential link to kidney and urinary system exposure	Exacoustos et al. (2023)
Urine	Polyethylene (PE), Polyvinyl chloride (PVC), Polyamide 66 (PA66), Acrylates (ACR), Polymethylmethacrylate (PMMA), Polyurethane (PU), Polypropylene (PP), Polyethylene terephthalate (PET)	Urine samples	100 nm - 5 mm (MNPs), <100 nm (nanoplastics)	Py-GC/MS: Average abundance of 1.50 (2.31) mg/kg, found in 9 samples. LDIR: Average abundance of 15.17 (23.13) particles/kg, found in 7 samples.	Potential urinary system impact, hormonal disruption from additives (such as plasticizers)	Healthy volunteers from urban and rural regions of Chongqing, China	Urban residents had significantly higher levels and a greater variety of MNPs; regular contact with plastic toys and use of personal care products were associated with MNP presence	Not specified	Song et al. (2024)
Central Nervous System (CNS)	Polystyrene (PS), Polyethylene (PE), Polypropylene (PP), Polyvinyl Chloride (PVC)	Cerebrospinal fluid (CSF)	Not specified; < 100 micron – fiber and fragment	PP and PE: Positively correlated with CSF albumin index	Potential neuroinflammation, BBB impairment	28 patients (with or without CNS infection)	Environmental exposure to plastic pollution	Positive correlation of PP and PE with CSF albumin index. No significant association with IL-6 or IL-8 levels.	Xie et al. (2024)
**Colon**	Polycarbonate, Polyamide, Polypropylene	Colectomy specimens	Not specified	Average of 331 particles per specimen (28.1 ± 15.4 particles/g tissue)	Potential inflammatory responses, gut microbiota disruption	11 adults (mean age 45.7, six males), residents of Northeastern Peninsular Malaysia	Not specified	Not specified	Ibrahim et al. (2021)
**Colon**	Polyethylene, Poly(methyl methacrylate), Nylon (Polyamide)	Tumoral colon tissues	1–613	702.68 ± 504.26 particles per 1g tissue	Potential association with colorectal cancer development	Patients diagnosed with colorectal adenocarcinoma	Not specified	Not specified	Cetin et al. (2023)
**Colon**	Polyethylene, Poly(methyl methacrylate), Nylon	Non-tumoral colon tissues	1–743	207.78 ± 154.12 particles per 1g tissue	Potential association with colorectal cancer	Patients diagnosed with colorectal	Not specified	Not specified	

	(Polyamide)				development	adenocarcinoma			
**Colon**	Polyethylene, Poly(methyl methacrylate), Nylon (Polyamide)	Control tissues	1–1299	218.28 ± 213.05 particles per 1g tissue	Not specified	Control subjects without colorectal cancer	Not specified	Not specified	
**Saphenous vein**	Alkyd resin, Poly(vinyl propionate/acetate), Nylon-ethylene-vinyl acetate (tie layer)	Vein tissue samples	1–613	Average of 29.28 ± 34.88 particles/g tissue (14.99 ± 17.18 after background subtraction)	Potential inflammatory responses; unknown clinical implications	5 samples analyzed; demographics not specified	Not specified	Not specified	Rotchell et al. (2023)
**Prostate**	Polyamide (Nylon 6), Polypropylene, Polyacrylic Acid, Poly(dimethylsiloxane)	Prostate tissue samples	<26; pellet, fibers and fragments	Detected in 6 out of 12 samples; not specified in terms of concentration	Potential implications for prostate health	12 male patients undergoing TUR-P surgery; age range not specified	Not specified	Not assesed	Demirelli et al. (2024)
**Coronary Artery**	PET, PA-66, PVC, PE	Arterial samples with atherosclerotic plaques	Not specified	156.50 ± 42.14 µg/g	Association with atherosclerosis development	Patients with coronary artery disease undergoing bypass surgery	Not specified	Not specified	Liu et al. (2024)
**Carotid Artery**	PET, PA-66, PVC, PE	Arterial samples with atherosclerotic plaques	Not specified	133.37 ± 60.52 µg/g	Association with atherosclerosis development	Patients with carotid atherosclerosis undergoing endarterectomy	Not specified	Not specified	
**Aorta**	PET, PA-66, PVC, PE	Arterial samples without plaques	Not specified	76.26 ± 14.86 µg/g	Potential implications for vascular health	Patients with aortic dissection undergoing replacement surgery	Not specified	Not specified	
**Head Hair**	Polyethylene, polyethylene terephthalate, polypropylene		<100	3.5 per day	Unknown	Adults from various demographics across Iran	Urban vs. remote areas; higher counts in humid environments	Not specified	Abbasi and Turner (2021)
**Saliva**	Polyethylene, polyethylene terephthalate, polypropylene		<100	0.33 per day	Unknown	Adults from various demographics across Iran	Urban vs. remote areas; higher counts in humid environments	Not specified	
**Stool**	Polypropylene, polyethylene terephthalate	Stool	50–500	Median of 20 per 10 g of stool	Unknown	8 healthy adults aged 33–65 from Europe and Asia	No specific factors reported	Not specified	Schwabl et al. (2019)
	High-Density Polyethylene (HDPE) and other microplastics	Stool	Not specified	3.33–13.99 µg/g stool	Unknown	Fishermen community in Surabaya, Indonesia	Coastal dwellers with potential exposure to marine pollutants	Not specified	Luqman et al. (2021)

Table S2. Summary of Microplastic Detection in Human Biological Samples Across Various Countries

Country	Sample Type	Detection Method	Mode of Detection	Limit of Detection Reported	Quality Control Contamination Measures	Reference
Czech Republic	Amniotic fluid (n=10), Placenta (n=10)	Fourier Transform Infrared Spectroscopy – Attenuated Total Reflectance (FTIR-ATR)	Single	Not explicitly stated (smallest analyzed particle: 1 µm)	Non-plastic protocol (glass syringes, metal tools, sterile steel needles). Chemicals filtered (1 µm pore size). Lab ventilation turned off, doors/windows closed during handling. Blank samples (6 Petri dishes; 1 non-fiber contaminant detected). Fibers excluded from analysis.	**Halfar et al., 2023 (Chemosphere 343, 140301)**
USA	Penile tissue (5)	Agilent 8700 Laser Direct Infrared (LDIR) Chemical Imaging System; SEM (Zeiss Merlin)	Single	LDIR: 20–500 µm; SEM: 2 µm	Class 100 clean benches; filtered reagents (0.4 µm PC membranes); cleaned glassware (Milli-Q-water, ethanol, heated at 250°C); procedural blanks; synthetic polystyrene gloves; glass storage (control: polypropylene container)	**Codrington et al. (2024), IJIR: Your Sexual Medicine Journal 37:377–383**
Italy	Human urine (n=10)	MicroRaman spectroscopy	Single	Not explicitly stated	Plastic-free protocol; glassware washed with detergent, rinsed with distilled water and ethanol; procedural blanks analyzed; KOH pellets and deionized water tested for contamination; pristine filters scanned for particles.	**Massardo et al., 2024**
Italy	Human kidney tissue (n=10)	MicroRaman spectroscopy	Single	Not explicitly stated	Plastic-free protocol; glassware washed with detergent, rinsed with distilled water and ethanol; procedural blanks analyzed; KOH pellets and deionized water tested for contamination; pristine filters scanned for particles.	**Massardo et al., 2024**
Iran	Urine (30), Sputum (30), BALF (30)	Stereo optical microscopy, µ-Raman spectroscopy	Single	20 µm (lower), 500 µm (upper)	Controls with distilled water, ethanol, saline; lab coats, gloves; cleaned glassware	**Jahedi et al., 2025**
China	Human thrombi (26)	Raman Spectroscopy	Single	1 µm	Strict QC-PD system, blank/negative controls, contrast agent analysis	**Wu et al., J. Adv. Res. 49 (2023) 141-150, DOI: 10.1016/j.jare.2022.09.004**
Thailand	Human breast milk (59)	Raman micro-spectroscopy	Single	Not specified	All-plastic-free protocol, aseptic collection, 2% chlorhexidine solution for breast cleansing, sterile gloves, first drops discarded, samples stored at -20°C	**Saraluck et al., 2024**
China	Human thrombi (30)	Py-GC/MS, LDIR, SEM	Multi-Modal	Py-GC/MS: LOD/LOQ based on procedural blanks; LDIR: 20–500 µm	Cotton lab coats, polymer-free gloves, filtered reagents, blank controls (catheters, guidewires, containers, instruments), negative controls (sampling, procedural)	**Wang et al., 2024**
Turkey	Tumoral colon tissues (16), Non-tumoral colon tissues (16), Control colon tissues (15)	ATR-FTIR spectroscopy, Raman spectroscopy, optical microscope	Multi-Modal	Not specified	Samples not treated with alcohol, xylene, or paraffin; stored in 10% formaldehyde	**Cetin et al. (2023), Environ Chem Lett**
Turkey	Stomach contents (26)	µ-Raman spectroscopy	Single	0.45 µm	Rigorous cleaning of equipment, use of pre-filtered solvents, dummy samples for contamination control, analysis in laminar flow cabinet	**Özsoy et al., Forensic Science International 364 (2024) 112246**
China	Ileum (10), Mesenteric adipose tissue (10), Uninvolved ileum (6), Uninvolved mesenteric adipose tissue (6)	Laser Direct Infrared (LD-IR) imaging spectrometer	Single	20-500 µm	Filtered solvents, stainless steel membranes, blank controls, plastic-free lab materials	**Wu et al., 2025**
China	Feces (24)	Fourier transform infrared micro-spectroscopy (FTIR)	Single	0.01–5 mm	Quality control sample using ultrapure Milli-Q water; sterile fecal collection; pre-treatment and measurement steps for contamination control	**Zhang et al., 2021, Science of the Total Environment 767, 144345**
Indonesia	Feces of pregnant women (30)	Fourier Transform Infrared (FTIR) microspectroscopy, microscopy	Single	0.2 mm (minimum detectable size)	Use of glass bottles with aluminum covers, sample incubation, blank controls, equipment washed with distilled water	**Hasanah et al., 2024, Environmental Health Insights**
Japan, Russia, Netherlands, United Kingdom, Italy,	Human stool (8)	Fourier-transform infrared (FT-IR) microspectroscopy	Single	50 µm (particles <50 µm not investigated)	Procedural blank sample; metal spoons; glass containers; predefined sampling instructions	**Schwabl et al. (2019), Annals of Internal Medicine, DOI: 10.7326/M19-0618**

Poland, Finland, Austria						
Indonesia	Human stools (11)	Raman spectroscopy	Single	Not specified	Glass/steel utensils during collection/preparation, Fenton solution digestion, HNO3 digestion	**Luqman et al., 2021**
Austria	Human stool (15)	FTIR Spectroscopy	Single	0.046–3.0 particles/g (size-dependent)	Cleanroom, laminar flow bench, procedural blanks, field blanks, chemical resistance testing	**Hartmann et al., 2024**
China	Stools (78)	Pyrolysis gas chromatography-mass spectrometry (Py-GCMS)	Single	Provided in supplementary material 5	Three blank samples, procedural blanks, spiked recovery test, non-plastic principle during sampling	**Song et al., 2025**
Indonesia	Human stool (11), Drinking water (3), Staple foods (12), Table salts (2), Toothpaste (2)	Raman spectroscopy (785 nm)	Single	Not specified	Glass/steel utensils during collection and processing, Fenton's reagent digestion, HNO_3 digestion	**Wibowo et al., 2021, Sustainability**
China	Preschoolers' stools (69)	Pyrolysis-gas chromatography coupled with mass spectrometry (Py-GC/MS)	Single	LOD and LOQ were three and ten times the baseline noise, respectively (Supplementary Table S5)	Researchers wore cotton lab coats and polymer-free nitrile gloves; reagents filtered with PTFE membrane; glass utensils washed with filtered alcohol; sampling and procedural blanks used	**Ke et al., 2023**
China	Human tumor tissues (61)	Pyrolysis-gas chromatography-mass spectrometry (Py-GCMS)	Single	PS: 0.02 ng/g, PVC: 0.02 ng/g, PE: 0.5 ng/g	Glass and metal instruments rinsed with filtered deionized water, cotton lab coats, latex gloves, laminar flow hood, blank controls	**Zhao et al. (2024), Ecotoxicology and Environmental Safety 283, 116818**
Malaysia	Human colectomy specimens (11)	Chemical digestion, filtration, stereo- and FTIR microscopy	Single	Not specified	Cotton lab gowns, latex gloves, filtered reagents, cleaned apparatus, contamination chambers, systematic blanks	**Ibrahim et al. (2020), JGH Open**
China	Human lung tissues (100)	μ-FTIR, LDIR, Raman	Multi-Modal	>20 μm (μ-FTIR), <20 μm (Raman)	Procedural blanks, glassware cleaning, controlled air exposure	**Chen et al. (2022)**
United Kingdom	Human lung tissue (13)	μFTIR spectroscopy	Multi-Modal	3 μm	Procedural blanks, laboratory blanks, filtered reagents, glassware cleaning, cotton lab coats, nitrile gloves, low-activity conditions	**Jenner et al., 2022, Science of the Total Environment 831, 154907**
China	Lung tissue (12)	Agilent 8700 laser infrared imaging spectrometer, SEM	Single	20–500 μm	Metal instruments, glassware, procedural blanks, high matching degree (≥0.80)	**Wang et al., 2023**
Brazil	Human lung tissue (20)	Raman spectroscopy	Single	0.5 μm	Plastic-free protocol, procedural blanks, stillborn controls	**Amato-Lourenço et al. (2021), Journal of Hazardous Materials 416, 126124**
Lithuania	Bronchoalveolar lavage fluid (BALF) (10)	Optical microscopy, TEM-EDX	Single	Not specified	Plastic-free laboratory protocols, blank measurements, glassware use, filtered reagents	**Uoginté et al., Heliyon 9 (2023) e19665**
China	Sputum (22)	Agilent 8700 laser infrared imaging spectrometer, Fourier-transform infrared microspectroscopy (MIR)	Multi-Modal	20–500 μm	Reagents filtered with 0.45 μm silver membrane; procedural blanks; glassware rinsed with deionized water	**Huang et al., 2022, Environ. Sci. Technol.**
Iran	Sputum (13), BALF (11), Pleural Fluid (10)	Digestion, density separation, Micro-Raman, SEM/EDS	Single	Not specified	Blank tests, procedural blanks, recovery tests, plastic-free lab protocols	**Momeni et al., 2025**
Spain	Bronchoalveolar lavage fluid (44)	μ-FTIR, SEM-EDS	Single	20 μm	Procedural blanks, solvent blanks, airborne blanks, bi-distilled water blanks, natural fabric clothing, autoclaved glassware	**Baeza-Martínez et al., 2022**
China	Nasal lavage fluid (113)	Polarized light microscopy, Laser Direct Infrared (LDIR)	Single	Not specified	Cleaned glassware, filtered ethanol, aluminum foil covers, procedural blanks	**Zhang et al., 2024**
Turkey	BAL (18), Blood (18)	μ-Raman spectroscopy	Single	Not specified	Glass/metal apparatus, ethanol cleaning, filtered water, cotton gowns, control samples for contamination	**Alpaydin et al., 2024**
China	Sputum (16), Nasal lavage fluid (16)	Polarized light microscopy, Laser direct infrared (LDIR) chemical imaging system	Single	Not specified	Blank control established during experiment	**Jiang et al., 2022**
Republic of Korea	Nasal hair (86), Inferior turbinate (93), Middle turbinate (51), Nasopharyngeal fluid (129), Middle nasal cavity fluid (31)	μ-FTIR (Thermo Scientific Nicolet iN10 microscopy)	Single	Not specified	Procedural blank (6, n=3), Laboratory blank (1, n=3)	**Min et al., 2024, DOI: 10.1002/air.23427**
China	Semen (113), Urine (113)	Raman microscopy	Single	1.2 μm	Glass containers heated at 500°C for 8h, sterile conditions, P2-level laboratory, blank controls	**Zhang et al., 2024, eBioMedicine**
Italy	Human semen (10)	Raman Microspectroscopy	Single	1.2 μm pore-size filtration	Sterilized glassware, cotton lab coats, steel tools, single-use latex gloves, environmental and procedural	**Montano et al., 2023**

					blanks	
China	Semen (40)	Confocal Raman microscope	Single	0.25 μm	ISO Class 5 cleanroom, glassware pre-cleaned at 450°C, cotton lab wear, blank controls	**Li et al., 2024**
China	Human semen (3)	Pyrolysis-gas chromatography/mass spectrometry (Py-GCMS)	Single	Not specified	Quality assurance and quality control described in Text S1	**Chen et al. (2024), Environmental Chemistry Letters**
USA	Canine testis (47), Human testis (23)	Pyrolysis-gas chromatography/mass spectrometry (Py-GC/MS)	Single	Not specified	Tissue digestion, ultracentrifugation, anonymized samples, consented donations	**Hu et al., 2024, Toxicological Sciences**
United Kingdom	Human urine (38)	μFTIR spectroscopy, SEM-EDX	Single	5 μm	Procedural blanks (n=15), pre-filtered reagents, glassware cleaning, metal catheter samples	**Rotchell et al., 2024**
China	Testis (6), Semen (30)	Py-GC/MS, LD-IR	Single	1 μm	Plastic-free principle, glass containers, Milli-Q water blanks, fume hood, flame-cauterized glass devices, PTFE membranes	**Zhao et al., 2023, Science of the Total Environment 877, 162713**
Italy	Human placenta (6)	Raman Microspectroscopy	Single	~5 μm	Plastic-free protocol, cotton gloves, metal tools, procedural blanks	**Ragusa et al., 2021, Environment International 146, 106274**
China	Placentas(18), Meconium(12), Infant Feces(12), Breastmilk(7), Infant Formula(5)	Agilent 8700 laser infrared imaging spectrometer (LDIR)	Single	20–500 μm	Procedural blanks, glass/metal utensils, nitric acid digestion, stainless steel membrane filtration	**Liu et al., 2023**
Iran	Human placenta (43)	Digital microscopy, Raman microspectroscopy	Single	Not specified	Plastic-free protocol, cotton gloves, pre-washed cotton towels, ultrapure water, filtered reagents, QC samples	**Amereh et al., 2022**
USA	Human placental specimens (62)	Pyrolysis gas chromatography mass spectrometry (Py-GC-MS), Fluorescence microscopy, Attenuated total reflectance-Fourier transform infrared spectroscopy (ATR-FTIR)	Multi-Modal	Py-GC-MS: Not explicitly stated; ATR-FTIR: 20 μm; Fluorescence microscopy: 0.5 μm	Polymer standard curves, blank samples, analysis of plastic tubes used in study, ultracentrifugation process confirmed no detectable polymers	**Garcia et al., 2024, Toxicological Sciences, 199(1), 81–88**
Germany, Austria	Placenta (2), Meconium (2), Maternal stool (1)	Fourier-transform infrared (FTIR) microspectroscopy	Single	>50 μm	Cleanroom conditions, negative controls, contamination controls, procedural blanks, air controls	**Braun et al., 2021, Pharmaceutics**
China	Maternal blood (12), fetal appendages (12), umbilical vein blood (12)	Laser Direct Infrared Spectroscopy (LDIR)	Single	20–500 μm	Blank controls (normal saline), no plastic use during sampling, glassware only	**Sun et al., 2024, Ecotoxicology and Environmental Safety 287, 117300**
China	Uterine fibroids (48), Myometrium (48)	Raman spectroscopy	Single	<1 μm	Lab coat, sterile gloves, procedural blanks, pre-filtration, sanitization	**Xu et al., 2024**
China	Amniotic fluid (40)	Laser direct infrared (LD-IR) spectroscopy	Single	Not specified	Plastic-free procedures, nitrile gloves, cotton lab coats, surgical masks, head covers, fume hood, stainless steel membrane filtration, flame sterilization of glassware, blank samples	**Xue et al., 2024**
Italy	Ovarian follicular fluid (18)	SEM-EDX	Single	0.1 μm	Glassware/metal equipment, filtered water/acetone rinsing, dust-free nitrile gloves, cotton lab coats, clean room with laminar flow, reagent blanks	**Montano et al., 2025**
China	Meconium (16)	Fourier Transform Infrared Microspectroscopy (micro-FTIR), Ultra-depth three-dimensional microscope	Single	≥10 μm	Clean room, cotton lab coats, latex gloves, ultra-pure water rinsing, diaper controls, procedure blank controls	**Li et al., 2023, Toxics**
China	Cumulus granulosa cells (16), Follicular fluid (2)	Pyrolysis-gas chromatography/mass spectrometry (Py-GC/MS)	Single	Not specified	Not specified	**L. Weina et al., Abstract citation ID: deae108.1081**
China	Blood (15), Cancerous tissue (15), Paracancerous tissue (15)	Raman spectroscopy, Py-GC/MS	Multi-Modal	Not specified	Cleaned glass/metal tools, procedural blanks, filtered reagents, dust-free suits	**Xu et al., 2024 (Environmental Research 259, 119498)**
Italy	Human kidney (10), Human urine (10)	Light microscope (500x) coupled to Raman spectrometer (Renishaw System 2000)	Single	0.2 μm (filter pore size)	"Plastic-free" protocol, procedural blanks	**Exacoustos et al., 2023**
Germany	Liver (11), Kidney (3), Spleen (3)	Chemical digestion, Nile red staining, fluorescent microscopy, Raman spectroscopy	Single	4-30 μm	Clean working environment, pre-cleaning of devices, pre-filtration of solutions, blank samples analyzed	**Horvatits et al., 2022**
USA	Human brain (28), liver (20), kidney (20)	Pyrolysis gas chromatography–mass spectrometry (Py-GC/MS), attenuated total reflectance–Fourier transform infrared spectroscopy (ATR–FTIR), electron microscopy with energy-dispersive spectroscopy (SEM-EDS)	Multi-Modal	Not explicitly stated	KOH and formal storage control sample blanks, polymer composition analysis of plastic tubes and pipette tips	**Nihart et al. (2025), Nature Medicine, DOI: 10.1038/s41591-024-03453-1**
Italy	Urine (6)	Raman microspectroscopy	Single	1.2 μm	Plastic-free protocol, glass tools,	**Pironti et al., Toxics 2023, 11, 40**

					environmental/procedural blanks, ethanol cleaning	
China	Urine (12)	Py-GC/MS, LDIR	Multi-Modal	Not specified	Plastic-free protocols, blank controls, pre-filtered chemicals, cotton lab coats, nitrile gloves, fume hood use	**Song et al., 2024**
Netherlands	Human whole blood (22)	Pyrolysis-GC/MS	Single	PMMA: 0.10 µg/ml, PP: 0.68 µg/ml, PS: 0.34 µg/ml, PE: 0.61 µg/ml, PET: 0.13 µg/ml	Sampling blanks (n=5), procedural blanks (n=31), recovery experiments, duplicate analyses	**Leslie et al., 2022**
United Kingdom	Human blood (20)	µFTIR spectroscopy	Single	≥70% spectral match	Procedural blanks (n=10), pre-filtered reagents, glassware cleaning, contamination adjustments (LOD and LOQ)	**Leonard et al., 2024**
Republic of Korea	Human whole blood (36)	µ-FTIR, µ-Raman	Multi-Modal	5 µm (µ-FTIR)	Glass/metal tools, laminar flow bench, procedural blanks, recovery testing	**Lee et al., 2024, Scientific Reports**
China	Blood (39), Fecal (34)	Pyrolysis-gas chromatography/mass spectrometry (Py-GC/MS)	Single	Not specified	Blank samples for instrument and method, standard samples for precision, recovery rate (90%-110%)	**Gao et al. (2025), Ecotoxicology and Environmental Safety 290, 117497**
Italy	Carotid artery plaque (257)	Pyrolysis–gas chromatography–mass spectrometry, stable isotope analysis, electron microscopy	Multi-Modal	Not specified	Preventive measures applied (details not specified), residual risk acknowledged	**Marfella et al., N Engl J Med. 2024;390(10):900–910. doi:10.1056/NEJMoa2309822**
China	Coronary artery (4), Carotid artery (7), Aorta (6)	Pyrolysis-gas chromatography/mass spectrometry (Py-GC/MS)	Single	Not specified	Filtered reagents, flame-cleaned glassware, cotton lab coats, nitrile gloves, aluminum foil-covered clean bench, plastic-free environment	**Liu et al., Journal of Hazardous Materials 469 (2024) 133855**
Brazil	Olfactory bulb tissue (15)	Micro-Fourier transform infrared spectroscopy (µFTIR)	Single	Not specified	Plastic-free approach, prefiltered solutions, stainless steel/glassware, cotton lab coats, clean latex gloves, laminar flow cabinet, blank filters, restricted access to analysis rooms	**Amato-Lourenço et al., JAMA Network Open, 2024, doi:10.1001/jamanetworkopen.2024.40018**
China	Cerebrospinal fluid (28)	Pyrolysis-gas chromatography-mass spectrometry (Py-GC/MS), Laser direct infrared (LDIR) imaging spectroscopy	Multi-Modal	Table S2 (specific values not provided in text)	Glass materials used, blank controls, filtered solutions, sealed storage	**Xie et al., Journal of Hazardous Materials 480 (2024) 136028**
China	Gallstones (16)	Laser Direct Infrared (LDIR), Pyrolysis Gas Chromatography–Mass Spectrometry (Py-GCMS)	Multi-Modal	Not specified	Strict precautions to avoid contact with plastic during sample handling; use of glass bottles and stainless-steel tools	**Zhang et al., Journal of Hazardous Materials, 2024**
China	Bone (8), Cartilage (8), Intervertebral Disc (8)	Raman Microspectroscopy	Single	Not specified	Laboratory cotton coats, clean nitrile gloves, filtration devices rinsed and filtered three times	**Yang et al., 2025**
Netherlands	Synovial fluid (10)	Raman spectroscopy, SEM-EDX	Single	Not specified	Measured blank samples for baseline comparison	**Niessink et al., Journal of Raman Spectroscopy, 2025**
China	Human synovium (45)	Micro-Fourier Transform Infrared Spectroscopy (µ-FTIR), Scanning Electron Microscopy (SEM), Raman Microscopy	Single	Not specified	Plastic-free protocols, laminar-flow operating room, cotton lab coats, glassware cleaning, procedural controls, blank controls	**Li et al., Journal of Hazardous Materials 461 (2024) 132640**
Iran	Hair, hand skin, facial skin, saliva, face masks (1140)	Micro-Raman spectroscopy, binocular microscope	Single	100–5000 µm	Clean room, cotton lab coats, ethanol-cleaned surfaces, filtered reagents, blank samples	**Shahsavaripour et al., 2023**
Iran	Head hair (2000), Hand skin (2000), Face skin (2000), Saliva (2000)	Binocular microscopy, Polarized light microscopy, Fluorescence microscopy, µ-Raman spectroscopy	Single	5 µm	Pre-cleaned glassware, filtered reagents, ethanol-wiped surfaces, closed windows/doors, lab coats, gloves, facemasks, control samples	**Abbasi and Turner, Journal of Hazardous Materials 403 (2021) 123799**
China	Aqueous humor (90)	Pyrolysis-gas chromatography/mass spectrometry (Py-GC/MS)	Single	Not explicitly stated	Ultra-pure water controls, PTFE membrane filtration, glassware use, preheated crucibles, blank tests, nitrile gloves, cotton lab coats	**Zhang et al., 2025, iScience 28, 112078**
China	Human vitreous humor (49)	Laser Direct Infrared (LD-IR), Pyrolysis Gas Chromatography/Mass Spectrometry (Py-GC/MS)	Multi-Modal	LD-IR: ≥20 µm, Py-GC/MS: No lower size limit	Pre-filtered reagents, glass utensils, procedural blanks, spike-and-recovery tests	**Zhong et al., Science of the Total Environment 921 (2024) 171109**
China	Human endometrium (20)	Laser Direct Infrared (LDIR) spectroscopy	Single	20–500 µm	Glass containers, nitric acid digestion, procedural blanks, filtered reagents	**Sun et al., Science of the Total Environment 906 (2024) 167760**
China	Paired paratumor and tumor prostate tissues (22)	Laser Direct Infrared Spectroscopy (LDIR), Scanning Electron Microscopy (SEM), PyrolysisGas ChromatographyMass Spectrometry (PyGC/MS)	Multi-Modal	Particle size: 20500 µm (LDIR), matching score >0.65	Nitrile gloves, cotton lab coats, filtered reagents, glassware rinsed with ethanol, blank tests for contamination	**Deng et al. (2024), eBioMedicine**
Canada	Stool (23), Liver biopsies (17)	FTIR spectroscopy, Optical microscopy	Single	Not specified	MP-free reagents, glass/metal tools, cotton lab coats,	**Schwenger et al., 2024**

					purple nitrile gloves, designated enclosure, blanks for contamination assessment	
Republic of Korea	Human cervicovaginal lavage fluids (10)	Raman spectroscopy	Single	5 µm	Glass materials, laminar flow hood, pre-filtered solutions, nitrile gloves, cotton lab coats, blank samples	**Shim & Min, 2025**
Netherlands	Whole blood (68)	Pyrolysis-gas chromatography-mass spectrometry (Py-GC-MS)	Single	31 ng/mL (PMMA) to 825 ng/mL (PVC)	Pre-cleaning of glassware, laminar flow cabinet, procedural blanks, QC samples, avoidance of plastic materials	**Brits et al. (2024)**
China	Bone marrow (16)	Py-GC/MS, LD-IR, SEM	Multi-Modal	Not specified	Cotton lab coats, nitrile gloves, triple-filtered reagents, glass utensils, procedural blanks, recovery experiments	**Guo et al., 2024, Journal of Hazardous Materials 477, 135266**
Turkey	Prostate tissue (12)	Light microscopy, ATR-FTIR spectrophotometry	Single	2.5 µm	Sterile non-latex gloves, filtered solutions, laminar flow cabinet, blank experiments	**Demirelli et al. BMC Urology (2024) 24:106**
Canada	Placenta (10)	Raman Micro-spectroscopy	Single	~2 µm	Plastic-reduced protocol, procedural blanks, hood blanks, filtered water rinses, natural fiber clothing	**Zurub et al., 2024**
China	Feces (102)	Micro-Raman spectroscopy	Single	0.5 µm (space resolution)	Labware washed with ultrapure water, filters tested for MPs, reagents filtered, plastic-free lab clothes, QC sample with ultrapure water	**Yan et al., Environ. Sci. Technol. 2022, 56, 414–421**
China	Bronchoalveolar lavage fluid (77)	Confocal micro-Raman spectrometer	Single	1 µm	Filtered reagents, rinsed glassware, procedural/sample blanks, cotton lab coats/gloves	**Chen et al. (2023)**
China	Bronchoalveolar lavage fluid (32), Cigarette smoke (10), Control (6)	Laser direct infrared spectroscopy (LDIR)	Single	20–500 µm	Glass containers, steel membranes, autoclaved metal bowls, subtraction and LOD/LOQ methods	**Lu et al., 2023, Environ. Sci. Technol. 57, 8496–8505**
China	Bronchoalveolar lavage fluid (18)	Laser direct infrared spectroscopy (LDIR), Scanning electron microscopy (SEM)	Single	20–500 µm	Plastic-minimized method, control samples, glass containers, filtered solutions, reduced air exposure	**Qiu et al., 2023, Environ. Sci. Technol.**
China	Feces (86), Urine (60)	LD-IR, TD-GC/MS	Multi-Modal	0.22 µm	Procedural blanks, ultrapurified water samples, tool cleaning (muffle furnace), glass fiber membrane baking	**Zhu et al., 2024, Environ. Sci. Technol.**
United Kingdom	Human saphenous vein tissue (n=5)	µFTIR spectroscopy	Single	5 µm	Procedural blanks, pre-filtered reagents, glassware cleaning, cotton lab coats, nitrile gloves, controlled environment	**Rotchell et al. (2023), PLOS ONE**
China	Placentas (18), Meconium (12)	Agilent 8700 laser infrared imaging spectrometer (LDIR)	Single	Particles with diameters >20 µm counted (range: 20–500 µm)	Glass utensils, procedural blanks, vacuum-filtered reagents, recovery rates (84–97%)	**Liu et al., 2023**
China	Tear fluid and meibum (45)	LDIR, SEM, Py-GC-MS	Multi-Modal	Not specified	Strict plastic-free protocol, nitrile gloves, cotton lab coats, surgical masks, hairnets, clean workbench, filtered reagents, glassware rinsed with ethanol	**Wang et al., Journal of Hazardous Materials 489 (2025) 137635**
China	Penile cancer tissue (17 patients)	Laser Direct Infrared (LDIR) spectroscopy	Single	20-500 µm	Particle-free nitrile gloves, cotton lab coats, glass containers, Milli-Q water blanks, ethanol-cleaned surfaces	**Wang et al., 2025, Science of the Total Environment 969, 178815**
China	Pericardia (6), Epicardial adipose tissue (6), Pericardial adipose tissue (11), Myocardia (3), Left atrial appendage (5), Pre/postoperative venous blood (7 pairs)	Laser Direct Infrared (LDIR) chemical imaging system, Scanning Electron Microscopy (SEM)	Single	20 µm (particles smaller than 20 µm not identifiable)	Vacuum pump filtration, cotton coats, surgical masks, latex gloves, pre-rinsed glass bottles, blank samples, laminar flow hood	**Yunxiao Yang et al., Environ. Sci. Technol. 2023, 57, 10911–10918**

Table S3. Particle Size Thresholds for Biological Barrier Penetration

Biological Barrier	Approx. Threshold	References
Intestinal Epithelium	~5000 nm (5 µm)	Walczak et al. 2015 (EFSA); Jani et al. 1989; Deng et al. 2017
Alveolar-Capillary Barrier	~1000 nm (1 µm)	Oberdörster et al. 2005; Geiser et al. 2005
Placental Barrier	~200 nm	Wick et al. 2010; Bové et al. 2019; Grafmueller et al. 2015
Blood-Brain Barrier (BBB)	~100 nm	Sharma & Sharma 2012; Kreyling et al. 2017
Skin (Intact Epidermis)	~40 nm	Larese Filon et al. 2013; Bouwstra et al. 2003; Mortensen et al. 2008

- **Walczak AP et al.** (2015). *Bioavailability and biodistribution of differently charged polystyrene nanoparticles upon oral exposure in rats.* Journal of Nanoparticle Research, 17(2), 66.

- **Jani PU et al.** (1989). *Nanoparticle uptake by the rat gastrointestinal mucosa: quantitation and particle size dependency.* Journal of Pharmacy and Pharmacology, 41(12), 821–826.

- **Deng Y et al.** (2017). *Tissue accumulation of microplastics in mice and biomarker responses suggest widespread health risks of exposure.* Environmental Science & Technology, 51(23), 13697–13706.

- **Geiser M, Kreyling WG.** (2010). *Deposition and biokinetics of inhaled nanoparticles.* Particle and Fibre Toxicology, 7(1), 2.

- **Oberdörster G et al.** (2005). *Nanotoxicology: an emerging discipline evolving from studies of ultrafine particles.* Environmental Health Perspectives, 113(7), 823–839.

- **Wick P et al.** (2010). *Barrier capacity of human placenta for nanosized materials.* Environmental Health Perspectives, 118(3), 432–436.

- **Bové H et al.** (2019). *Ambient black carbon particles reach the fetal side of human placenta.* Nature Communications, 10(1), 3866.

- **Grafmueller S et al.** (2015). *Transfer studies of polystyrene nanoparticles in the ex vivo human placental perfusion model: key parameters influencing translocation and accumulation.* Journal of Nanobiotechnology, 13(1), 52.

- **Sharma HS, Sharma A.** (2012). *Nanoparticles aggravate excitotoxic brain injury and blood-brain barrier breakdown.* Progress in Brain Research, 214, 143–171.

- **Kreyling WG et al.** (2017). *In vivo integrity of polymer-coated gold nanoparticles.* Nature Nanotechnology, 12(2), 141–146.

- **Larese Filon F et al.** (2013). *Nanoparticles skin absorption: new aspects for a safety profile evaluation.* Regulatory Toxicology and Pharmacology, 67(2), 218–226.

- **Bouwstra JA et al.** (2003). *Structure of the skin barrier and its modulation by vesicular formulations.* Progress in Lipid Research, 42(1), 1–36.

- **Mortensen LJ et al.** (2008). *Quantification of skin penetration of nanoparticles in a novel 3D tissue-engineered skin model.* Journal of Investigative Dermatology, 128(10), 2505–2515.

****
